# Supplementary material for: Development of clinical immunity to Plasmodium vivax following repeat controlled human malaria infection
Source: Nat Commun. Author manuscript; Available in PMC 2025 Oct 15. (PMC12462441; doi:10.1038/s41467-025-63104-y)
Supplement: Supplementary Appendix [file EMS209255-supplement-Supplementary_Appendix.pdf]

Development of clinical immunity to *Plasmodium vivax* following repeat controlled human malaria infection

Table of Contents

Supplementary Methods....2
VAC069 Study Inclusion and Exclusion Criteria....2
Severity grading of adverse events (AE)....5
Causality Assessment....5
Severity grading criteria for clinically significant laboratory abnormalities....7
Multiplex immunoassay....8
Anti-PvDBPII standardized enzyme-linked immunosorbent assay (ELISA)....11
Blood-stage inoculum preparation and CHMI....12
Malaria parasite quantification by qPCR....13
Thick blood film microscopy....15
Diagnostic algorithms used in VAC069 study....16
Modelling of parasite multiplication rate....16
Supplementary Figures....18
Figure S1. Primary and secondary *P. vivax* CHMI in the VAC079 study.....18
Figure S2. Parasite growth during *P. vivax* CHMI in the VAC069 study....20
Figure S3. Solicited adverse events during *P. vivax* CHMI in the VAC069 study.....21
Figure S4. Parasitaemia and solicited adverse events during heterologous rechallenge are similar to primary *P. falciparum* CHMI....23
Supplementary Tables....25
Table S1: Demographics of study participants.....25
Table S2: Unsolicited adverse events.....26
Table S3: Laboratory adverse events.....28
Table S4: Malaria qPCR data (gc/mL) for each CHMI....30
Table S5: Merozoite antigens tested in multiplex immunoassay....33
Supplementary References....34

Supplementary Methods

VAC069 Study Inclusion and Exclusion Criteria

A medical history and physical examination were conducted at the screening visit, as well as baseline blood tests including a full blood count; urea and electrolytes; liver function tests; and hepatitis B virus (HBV), hepatitis C virus (HCV), human immunodeficiency virus (HIV), Epstein-Barr virus (EBV) and Cytomegalovirus (CMV) serology. Dipstick urinalysis for all volunteers and urinary pregnancy testing for all female volunteers were also conducted at screening. Pregnancy testing (serum Beta Human Chorionic Gonadotropin, Beta hCG), was also carried out prior to and during CHMI. A full list of inclusion and exclusion criteria is shown below for the VAC069 study:

The volunteer must satisfy all the following criteria to be eligible for the study:

- Healthy adult aged 18 to 50 years.
- Red blood cells positive for the Duffy antigen/chemokine receptor (DARC).
- Normal serum levels of Glucose-6-phosphate dehydrogenase (G6PDH).
- Negative haemoglobinopathy screen.
- Able and willing (in the Investigator's opinion) to comply with all study requirements.
- Willing to allow the Investigators to discuss the volunteer's medical history with their General Practitioner.
- Volunteers with the potential to become pregnant only: Must practice continuous effective contraception* for the duration of the clinic visits (first 3 months post-CHMI).
- Agreement to permanently refrain from blood donation.
- Written informed consent to participate in the trial.
- Reachable (24/7) by mobile phone during the period between CHMI and completion of all antimalarial treatment.
- Willing to take a curative anti-malarial regimen following CHMI.
- Willing to reside in Oxford for the duration of the study, until antimalarials have been completed.
- Answer all questions on the informed consent quiz correctly.

* Female volunteers are required to use an effective form of contraception during the course of the study as malaria challenge could pose a serious risk to both maternal health and the unborn foetus. Acceptable forms of contraception for female volunteers include:

- Established use of oral, injected or implanted hormonal methods of contraception (an additional form of contraception will be required when taking the antimalarial medication, as this can interfere with the efficacy of hormonal contraception).
- Placement of an intrauterine device (IUD) or intrauterine system (IUS).
- Total abdominal hysterectomy.
- Barrier methods of contraception (condom or occlusive cap with spermicide).
- Male sterilisation, if the vasectomised partner is the sole partner for the subject.
- True abstinence, when this is in line with the preferred and usual lifestyle of the subject (periodic abstinence and withdrawal are not acceptable methods of contraception).

The volunteer may not enter the study if any of the following apply:

- History of clinical malaria (any species).
- Travel to a clearly malaria endemic locality during the study period or within the preceding six months.
- Use of systemic antibiotics with known antimalarial activity within 30 days of CHMI (e.g. trimethoprim-sulfamethoxazole, doxycycline, tetracycline, clindamycin, erythromycin, fluoroquinolones and azithromycin).
- Haemoglobin <120 g/L for a female volunteer or <130 g/L for a male volunteer prior to primary CHMI. (However, for enrolment into secondary and tertiary CHMIs slightly lower haemoglobin values (≤0.5 g/L) will be permitted at the discretion of the Investigator, to account for the blood volume donated during the previous CHMI).
- Receipt of immunoglobulins within the three months prior to enrolment.
- Receipt of blood transfusion at any time in the past.
- Peripheral venous access unlikely to allow twice daily blood testing (as determined by the Investigator).
- Receipt of an investigational product in the 30 days preceding enrolment, or planned receipt during the study period.
- Prior receipt of an investigational vaccine likely to impact on interpretation of the trial data or the *P. vivax* parasite as assessed by the Investigator.
- Planned receipt of a COVID-19 vaccine between 2 weeks before the day of CHMI until completion of antimalarial treatment

- Any confirmed or suspected immunosuppressive or immunodeficient state, including HIV infection; asplenia; recurrent, severe infections and chronic (more than 14 days) immunosuppressant medication within the past 6 months (inhaled and topical steroids are allowed).
- History of allergic disease or reactions likely to be exacerbated by malaria infection.
- Pregnancy, lactation or intention to become pregnant during the study.
- Use of medications known to cause prolongation of the QT interval ***and*** existing contraindication to the use of Malarone.
- Use of medications known to have a potentially clinically significant interaction with Riamet ***and*** Malarone.
- Any clinical condition known to prolong the QT interval.
- History of cardiac arrhythmia, including clinically relevant bradycardia.
- Disturbances of electrolyte balance, e.g. hypokalaemia or hypomagnesaemia.
- Family history of congenital QT prolongation or sudden death.
- Contraindications to the use of both of the proposed anti-malarial medications Riamet and Malarone.
- History of cancer (except basal cell carcinoma of the skin and cervical carcinoma in situ).
- History of serious psychiatric condition that may affect participation in the study.
- Any other serious chronic illness requiring hospital specialist supervision.
- Suspected or known current alcohol abuse as defined by an alcohol intake of greater than 25 standard UK units every week.
- Suspected or known injecting drug abuse in the 5 years preceding enrolment.
- Hepatitis B surface antigen (HBsAg) detected in serum.
- Seropositive for hepatitis C virus (antibodies to HCV) at screening or at C-7 (***unless*** has taken part in a prior hepatitis C vaccine study with confirmed negative HCV antibodies prior to participation in that study, and negative HCV RNA PCR at screening for this study).
- Positive family history in both 1st AND 2nd degree relatives < 50 years old for cardiac disease.
- Volunteers unable to be closely followed for social, geographic or psychological reasons.
- Any clinically significant abnormal finding on biochemistry or haematology blood tests, urinalysis or clinical examination. In the event of abnormal test results, confirmatory repeat

tests will be requested. Procedures for identifying laboratory values meeting exclusion criteria are shown in Appendix A.

- Any other significant disease, disorder, or finding which may significantly increase the risk to the volunteer because of participation in the study, affect the ability of the volunteer to participate in the study or impair interpretation of the study data.
- Inability of the study team to contact the volunteer's GP to confirm medical history and safety to participate.

Additional exclusion criteria for participants in VAC069C-E:

- Body weight <50 kg, as measured at screening

Severity grading of adverse events (AE)

Participant reported AEs were graded as mild, moderate or severe according to the following criteria:

- **GRADE 0:** None.
- **GRADE 1:** Transient or mild discomfort (< 48 h); no medical intervention/therapy required.
- **GRADE 2:** Mild to moderate limitation in activity – some assistance may be needed; no or minimal medical intervention/therapy required.
- **GRADE 3:** Marked limitation in activity, some assistance usually required; medical intervention/therapy required; hospitalization possible.

Causality Assessment

For each unsolicited AE, an assessment of the relationship of the AE to the study intervention(s) was undertaken. Alternative causes of the AE, such as the natural history of pre-existing medical conditions, concomitant therapy, other risk factors and the temporal relationship of the event to CHMI/ antimalarials were considered. The likely causality of all unsolicited AEs was assessed as per the criteria below:

- **No Relationship:** No temporal relationship to study intervention ***and*** alternate aetiology (clinical state, environmental or other interventions); ***and*** does not follow known pattern of response to study intervention.

- **Unlikely:** Unlikely temporal relationship to study intervention ***and*** alternate aetiology likely (clinical state, environmental or other interventions) ***and*** does not follow known typical or plausible pattern of response to study intervention.
- **Possible:** Reasonable temporal relationship to study intervention; ***or*** event not readily produced by clinical state, environmental or other interventions; ***or*** similar pattern of response to that seen with other similar interventions.
- **Probable:** Reasonable temporal relationship to study intervention; ***and*** event not readily produced by clinical state, environment, or other interventions ***or*** known pattern of response seen with other similar interventions.
- **Definite:** Reasonable temporal relationship to study intervention; ***and*** event not readily produced by clinical state, environment, or other interventions; ***and*** known pattern of response seen with other similar interventions.

Severity grading criteria for clinically significant laboratory abnormalities

Adapted from FDA guidelines using Oxford University Hospitals NHS Foundation Trust laboratory reference ranges.

**(Ox) Routine Haematology**		Unit	**Lab Range**	**Grade 0**	**Grade 1**	**Grade 2**	**Grade 3**
Haemoglobin	**Male**	g/l	130 - 170	126 - 170	115 - 125	100 - 114	<100
	**Female**		120 - 150	114 - 150	105 - 113	90 - 104	<90
White Blood Cells	**Elevated**	x109/l	4.00 - 11.00	3.51 - 11.49	11.50 - 15.00	15.01 - 20.00	>20
	**Low**				2.50 - 3.50	1.50 - 2.49	<1.50
Platelets		x109/l	150 - 400	136 - 400	125 - 135	100 - 124	<100
Neutrophils		x109/l	2.0 - 7.0	1.5 - 7.0	1.00 - 1.49	0.50 - 0.99	<0.50
Lymphocytes		x109/l	1.0 - 4.0	1.0 - 4.0	0.75 - 0.99	0.50 - 0.74	<0.50
Eosinophils		x109/l	0.0 - 0.5	0.0 - 0.64	0.65 - 1.50	1.51 - 5.00	>5.00

**Routine Biochemistry**		Unit	**Lab Range**	**Grade 0**	**Grade 1**	**Grade 2**	**Grade 3**
Sodium	**Elevated**	mmol/l	135 - 145	135 - 146	147 - 148	149 - 150	>150
	**Low**				132 - 134	130 - 131	<130
Potassium	**Elevated**	mmol/l	3.5 - 5.0	3.4 - 5.0	5.1 - 5.2	5.3 - 5.4	>5.4
	**Low**				3.2 - 3.3	3.0 - 3.1	<3.0
Urea		mmol/l	2.5 - 7.4	2.5 - 8.1	8.2 - 8.9	9.0 - 11.0	>11.0
Creatinine		µmol/l	49 - 104	49 - 113	1.1-1.5*ULN<br>114 -156	>1.5-3.0*ULN<br>157 - 312	>3.0*ULN<br>>312
Bilirubin	**Normal LFT**	µmol/l	0 - 21	0 - 26	1.3-1.5*ULN<br>27 - 31	>1.5-2.0*ULN<br>32-42	>2.0*ULN<br>>42
Bilirubin<br>*When accompanied by any increase in LFT*	**Abormal LFT**	µmol/l	0 - 21	0 - 22	1.1-1.25*ULN<br>23 - 26	>1.25-1.5*ULN<br>27 - 31	>1.5-1.75*ULN<br>>31
ALT		IU/l	10 - 45	10 - 55	1.25 - 2.5*ULN<br>*56 - 112*	>2.5 - 5.0*ULN<br>*113 - 225*	>5.0*ULN<br>*>225*
Alk Phosphatase		IU/l	30 - 130	30 - 142	1.1 - 2.0*ULN<br>*143 - 260*	>2.0 - 3.0*ULN<br>*261 - 390*	>3.0*ULN<br>*>390*
Albumin		g/l	32 - 50	32 - 50	28 - 31	25 - 27	<25

**Coagulation Studies**	Unit	**Lab Range**	**Grade 0**	**Grade 1**	**Grade 2**	**Grade 3**
Prothrombin time	s	9.0 - 12.0	9.0 - 13.1	1.10*ULN<br>13.2	>1.10 - 1.20*ULN<br>13.3 - 14.4	>1.2*ULN<br>>14.4
(APTT)<br>Activated Partial Thromboblasin Time	s	20.0 - 30.0	20.0 - 32.9	1.10 - 1.20*ULN<br>33.0 - 36.0	>1.20 - 1.40*ULN<br>36.1 - 42.0	>1.4*ULN<br>>42.0
Fibrinogen	g/l	1.5 - 4.0	1.5 - 4.0	1.25 - 1.49	1.0 - 1.24	<1.0

**Other**	Unit	**Lab Range**	**Grade 0**	**Grade 1**	**Grade 2**	**Grade 3**
AST<br>Aspartate transferase	IU/l	15 - 42	43 - 51	1.25 - 2.5*ULN<br>*52 - 105*	>2.5 - 5.0*ULN<br>*106 -210*	>5.0*ULN<br>*>210*
GGT<br>Gamma-glytamyl transpeptidase	IU/l	15 - 40	15 - 50	1.25 - 2.5*ULN<br>*50 - 100*	>2.5 - 5.0*ULN<br>*101 - 200*	>5.0*ULN<br>*>200*

Multiplex immunoassay

Recombinant protein expression and purification

Recombinant proteins covering the entire extracellular domain of seven *Plasmodium vivax* antigens and CD4 used as a control were expressed (details of constructs in **Table S5**). The PvEBP (KMZ83376.1) construct was generously provided by Prof. Gavin Wright, while Pv12 (PVX_113775), PvMSP1 (PVX_099980), PvGAMA (PVX_088910), and PvDBP (PVX_110810) were previously utilised in a study by Hostetler et al. [1], with plasmids sourced from Addgene and subcloned into the pTT3 expression plasmid, containing a Biolinker peptide and a C-terminal His tag. The extracellular domains of PvAMA1 (PVP01_0934200) and PvTRAg25 (PVP01_0000100) were determined using signal peptide analysis [2] and transmembrane prediction servers [3]. The designed constructs, with codons optimized based on the human genome and all predicted N-glycosylation sites (NXS/T) were mutated by converting the S/T to A in order to prevent inappropriate glycosylation events (*Plasmodium* parasites do not synthesise N-linked glycans). Constructs were synthesised by TWIST Biosciences, UK and cloned into a mammalian expression plasmid with a 5' mouse variable κ light chain signal peptide and an enzymatic biotinylation sequence/hexa-His tag at the C-terminus. PvDBP (PVX_110810) additionally incorporates a CD4 tag at the C-terminus. Plasmids were amplified, purified, and diluted to 1 mg/ml, then mixed with polyethyleneimine (PEI 40 kDa) in a 1:2.5 ratio for transfection in a 50 ml culture. The DNA/PEI mixture was incubated for 8 min before being added to HEK 293E cells cultured in Freestyle 293 media supplemented with Geneticin and 1% fetal bovine serum. Cell maintenance involved shaking at 125 rpm, 37°C, 5% CO_2, and 70% relative humidity.

To purify proteins from the culture supernatant, HEK 293E cells were pelleted at 3000 g for 20 min and subsequently filtered (0.22 µm, Corning). The culture supernatant was adjusted to 5 mM imidazole and 300 mM NaCl before being loaded onto Talon Sepharose, a cobalt-based immobilised metal affinity chromatography (IMAC) resin. The Talon Sepharose resin was initially equilibrated with binding buffer (50 mM sodium phosphate, 300 mM NaCl, pH 7.4), before culture supernatant was passed through the column to allow for protein binding to the beads. The column was washed with 20 ml of wash buffer (50 mM sodium phosphate, 300 mM NaCl, 5 mM imidazole, pH 7.4), before recombinant proteins were eluted with 5 ml of elution buffer (50 mM sodium phosphate, 300 mM NaCl, 150 mM imidazole, pH 7.4). The eluted fraction was subjected to buffer exchange

with PBS using 10 kDa Vivaspin Ultracel concentrators. Final concentrations were determined using a Nanodrop OneC UV–Vis Spectrophotometer (Thermo Fisher), and the purified proteins were run in 12% SDS PAGE to assess purity and stored at 4 °C until coupling.

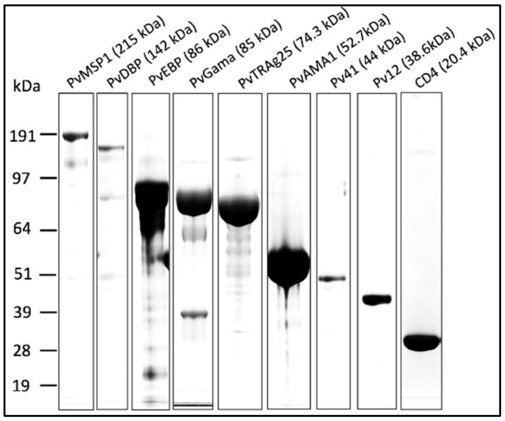

Protein production for multiplex immunoassay. Affinity-purified proteins were analysed by 12% SDS-PAGE to assess purity, with corresponding molecular weights indicated.

Protein conjugation to the Mag-Plex Luminex beads

Bead stocks (2.5x10^{6}/ml, Luminex, MC100XX-YY) were dispersed using a bath sonicator (30 s pulse, 10 s off). Subsequently, 0.4 ml of the bead stock (1x10^{6}/ml) was dispensed into low bind Eppendorf tubes, pelleted, and the supernatant was removed using a bar magnet. The beads underwent a wash with 1 ml sterile water (Sigma, W3500-500ml). A stock solution of EDC (1-ethyl-3(-3-dimethylaminopropyl) carbodiimide hydrochloride) and NHS (N-hydroxysuccinimide) was prepared in activation buffer (0.1 M NaH_2PO_4, pH 6.2) and 50 µl of EDC (Thermo, 22980) (50 mg/ml) and NHS (Thermo, 24500) (50 mg/ml) were added to the beads and vortexed gently. After a 20 min incubation at 25 °C on a rotator at 40 rpm, the supernatant was removed, and the beads were washed twice with 50 mM MES (4-morpholinoethanesulfonic acid) pH 5.0. Ten micrograms of each protein were added to the beads, and the reaction volume was adjusted to 1 ml with MES pH 5.0. The reaction mixture was incubated for 2 hours at 25 °C on a rotator at 40 rpm. Following incubation, the bead-coupled proteins underwent three washes with PBS-TBN (PBS 1X, 0.1% BSA, 0.05% Tween20, 0.05% sodium azide) and were finally resuspended in 1ml of PBS-TBN. Bead concentration was measured in a cell counter, and the beads were stored at 4 °C in the dark until further use. All steps were conducted in the dark, and magnetic base washing facilitated the separation of the beads.

The coupling of the beads was validated using flow cytometry. Approximately 10,000 antigen-coupled beads were aliquoted into an Eppendorf tube, and 5 µl of PE-conjugated Anti-his Ab (R&D system, IC050P) was added to each tube. The reaction mixture was incubated for 2 hours at 4 °C, followed by centrifugation at 12,000 rpm for 1 min and washing with PBS-TBN (this step was repeated twice). The beads were finally resuspended in PBS-TBN and analysed by flow cytometry (Thermo, Attune) with YL1 laser (561nm) for PE (561 585/16, laser power 400).

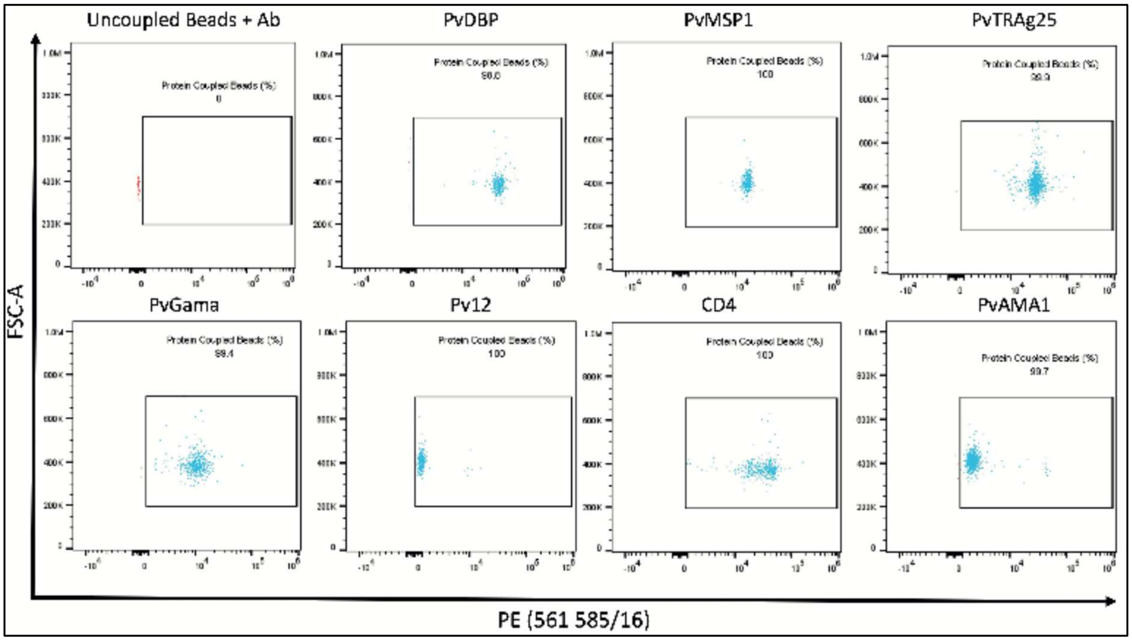

Protein bead conjugation for multiplex immunoassay. Flow cytometry dot plots validating protein bead conjugation. The Y-axis represents forward scatter area, while the X-axis displays the fluorescence signal from the anti-His antibody PE conjugate. Protein-coupled beads are depicted in the gated population (sky blue) in contrast to the uncoupled beads (red).

Luminex Assay

Each bead set underwent sonication and vortexing, and a master mix containing 25 beads of each region/µl was prepared in PBS-TBN. The total volume of the Bead Master mix, amounting to 12 ml, was generated by adding the required quantity of each protein-coupled bead for eight antigens, and the remaining volume was topped up with PBS-TBN. After another round of sonication and vortexing, 50 µl of the master mix was dispensed into each well in duplicate. The bead calculations are detailed in **Table S5**.

Human serum samples were heat-inactivated at 56 °C for 30 minutes, and a master plate was prepared with sera diluted at 1:200 in PBS-TBN. Subsequently, 50 µl of serum samples was added to plates containing 50 µl of beads, mixed five times, covered with aluminium foil, and incubated on a plate shaker at 200 rpm for 1 h at 25 °C. Following incubation, the plate was fixed on a magnetic base and washed five times (5 x 100 µl/well) with PBS-TBN, with each wash lasting 1 min. Fifty microliters of goat anti-human IgG secondary antibody (4 µg/ml) (Thermo, PA1-86078) was added to each well and incubated for 30 min at 25 °C. The plates were washed five times (5 x 100 µl) with PBS-TBN, fixed on a magnetic base, and finally resuspended in 100 µl PBS-TBN. Prior to the run, calibration of the instrument (Bio-Rad, BioPlex 200 Systems) was performed, and the plates were shaken for 5 min. A blank sample well, containing only the bead master mix, was included in the plate to subtract any background fluorescence from the data.

Anti-PvDBPII standardized enzyme-linked immunosorbent assay (ELISA)

ELISAs to quantify circulating PvDBPII-specific total IgG responses were performed using standardised methodology, similar to that previously described [4]. Nunc MaxiSorp ELISA plates (Thermo Fisher) were coated overnight (≥16 h) at 4 °C with 50 µL per well of 2 µg/mL PvDBPII protein. Plates were washed 6x with 0.05 % phosphate-buffered saline/Tween (PBS/T) and tapped dry. Plates were blocked for 1 h with 100 µL per well of Starting Block T20 (Thermo Fisher) at 20 °C. Test samples were diluted in blocking buffer (minimum dilution of 1:100), and 50 µL per well was added to the plate in triplicate. Reference serum (made from a pool of high-titer vaccinated donor serum) was diluted in blocking buffer in a three-fold dilution series to form a ten-point standard curve. Three independent dilutions of the reference serum were made to serve as internal controls. The standard curve and internal controls were added to the plate at 50 µL per well in duplicate. Plates were incubated for 2 h at 20 °C and then washed 6x with PBS/T and tapped dry. Goat anti-human IgG–alkaline phosphatase secondary antibody (Merck) was diluted 1:1000 in blocking buffer and 50 µL per well was added. Plates were incubated for 1 h at 20 °C. Plates were washed 6x with PBS/T and tapped dry. 100 µL per well of p-nitrophenyl phosphate alkaline phosphatase substrate (Thermo Fisher) was added, and plates were incubated for approximately 15 min at 20 °C. Optical density at 405 nm (OD_{405}) was measured using an ELx808 absorbance reader (BioTek) until the internal control reached an OD_{405} of 1.0. The reciprocal of the internal control dilution giving an OD_{405} of 1.0 was used to assign an arbitrary unit (AU) value of the standard. Gen5 ELISA software v3.04 (BioTek) was used to convert the OD_{405} of test samples into AU by

interpolating from the linear range of the standard curve fitted to a four-parameter logistic model. Any test samples with an OD_{405} below the linear range of the standard curve at the minimum dilution tested were assigned a minimum AU value of 5.0. These responses in AU are reported in µg/mL of PvDBPII following generation of a conversion factor by calibration-free concentration analysis (CFCA). In short, CFCA was performed using a Biacore X100 instrument, a Biotin CAP chip and X100 control and evaluation software (Cytiva). Purified mono-biotinylated antigen was produced for use in CFCA and chip was regenerated with manufacturer's supplied regeneration and CAP reagents and fresh antigen prior to each application of antibody. Serum samples, from a previous clinical trial (VAC051), with a range of PvDBP antibody responses were diluted and assessed for antigen-specific antibody binding and initial rates of antigen-specific binding at 5 µL/min and 100 µL/min measured and compared to permit measurement of concentration. The CFCA-measured PvDBP-specific antibody concentrations for each individual were analysed by linear regression with corresponding total IgG ELISA AU data, where slope of the line was used to derive an AU-to-µg/mL conversion factor.

Blood-stage inoculum preparation and CHMI

The *P. vivax* W1 clone blood-stage inoculum was thawed and prepared under strict aseptic conditions as previously described [5]. The required number of vials of the cryopreserved stabilate (each containing approximately 0.5 mL of red blood cells in 1 mL of Glycerolyte 57) were thawed in parallel in an area using solutions licensed for clinical use and single-use disposable consumables. A class II microbiological safety cabinet (MSC) was used to prepare the inoculum, which was fumigated with hydrogen peroxide and decontamination validated prior to use. To prepare the inoculum, 0.2 volume 12 % saline was added dropwise to the contents (about 1.5 mL) of each vial of thawed infected blood. Each sample was left for 5 min, before an additional 10 volumes of 1.6 % saline was added dropwise prior to centrifugation for 4 min at 830 x *g*. Each supernatant was removed and 10 mL of 0.9 % saline was added dropwise. The cell pellets were pooled and washed twice in 0.9 % saline before a final resuspension into one 10 mL sample in 0.9 % saline. This 10 mL suspension was then divided into aliquots, equivalent to one tenth of one original cryovial. Each aliquot was made up to a total volume of 5 mL in 0.9 % saline in a sterile syringe for injection and transported to the clinic. For each challenge, one dose of the 1:10 diluted inoculum was quantified

by quantitative polymerase chain reaction (qPCR) to be equivalent to between 165 to 217 genome copies of *P. vivax*. This will be an overestimate of the number of live viable parasites administered per participant because some parasites will be killed during the inoculum thawing and preparation process.

The reconstituted blood-stage inoculum (5 mL per syringe) was injected intravenously using an indwelling cannula, preceded and followed by a saline flush. The inoculum was administered to all participants within a maximum of 3 h 7 min from thawing of the inoculum. Participants were observed for 1 h following injection of the inoculum before discharge from the clinical facility. Following each CHMI, a leftover sample of the inoculum was cultured and shown to be negative for bacterial contamination.

For *P. falciparum* CHMI, cryopreserved blood-stage *P. falciparum* (3D7 clone) parasites were thawed and prepared under aseptic conditions using the similar methods as for *P. vivax* inoculum preparation and as previously described[6]. The target inoculum dose for *P. falciparum* inoculum was 1000 parasitised erythrocytes per participant, which was based on microscopic estimates of the donor's parasite density prior to freezing of the inoculum. Volunteers received infected red cells in a total volume of 5 mL normal saline, followed by a saline flush.

Malaria parasite quantification by qPCR

Quantitative (q)PCR was used to measure *P. vivax* parasitemia in participants' blood in real-time using an assay that targets the 18S ribosomal RNA (rRNA) gene as previously described[5]. DNA was extracted from 0.4 mL whole EDTA blood using a QIAsymphony SP robot, utilising the Qiagen DSP Blood Midi Kit and the pre-loaded Blood 400 v6 extraction protocol, with a 100 µL elution in ATE buffer selected. Additionally, aliquots of baseline samples taken within 2 days pre-CHMI were spiked with a known concentration of positive control DNA to check there was no presence of PCR inhibitors in participants' blood prior to CHMI.

Following DNA extraction, a standard Taqman absolute quantitation was used against a standard curve to amplify a 183 bp PCR product from the multi-copy, highly conserved 18S ribosomal RNA genes of *Plasmodium spp.* qPCR used the following adapted oligonucleotide primers and probe[7]: 18s forward primer 5'-AGG AAG TTT AAG GCA ACA ACA GGT-3', 18s reverse primer 5'-GCA ATA ATC TAT CCC CAT CAC GA-3' and shortened FAM labelled probe sequence 5'-TGA ACT

AGG CTG CAC GCG-3', was run on an ABI StepOne Plus machine with v2.3 software. Default Universal qPCR (target FAM-NFQ-MGB) and quality control (QC) settings were used apart from the use of 40 cycles and 25 μL reaction volume.

This qPCR detects DNA from pan-*Plasmodium* species, but unlike the synchronous growth of *P. falciparum*, circulating *P. vivax*-infected red blood cells may contain up to 10 to 15 individual genomes (in blood-stage late trophozoites and schizonts) and can also include the presence of gametocytes. The qPCR score is therefore reported in genome copies/mL (gc/mL) as opposed to a quantity of parasites.

The standard curve was generated from dilution of a linearised plasmid encoding part of the *Plasmodium spp.* 18S ribosomal RNA gene and calibrated using known *P. falciparum* (Pf) spiked blood samples initially and then reference DNA extracted from whole blood from *P. vivax*-infected patient samples in Thailand where parasites had been quantified by microscopy (kindly provided by Mahidol University). Based upon earlier results obtained using dilution series of microscopically-counted cultured Pf parasites, a Pf-specific 18S rRNA Taqman qPCR showed a lower limit of quantification (LLQ, defined as % covariance [CV] <20%) of around 20 Pf parasites (p)/mL blood[8]. Counted parasite dilution series results also suggested that the lower limit of probable detection (LLD, that is a probability of >50% of ≥1 positive result among three replicate qPCR reactions) is in the region of 5 p/mL, whereas samples at 1 p/mL are consistently negative (24/24 qPCR reactions). Positive results in this assay (even at very low detection) are thus essentially 100 % specific for genuine parasitemia, with positive results beneath the LLQ likely to signify parasitemia in the range of 2 to 20 p/mL. Similar sensitivity in terms of genome copy detection was observed when using the pan-*Plasmodium* qPCR described above and the diluted *P. vivax*-infected patient blood test samples from Thailand. As noted, these samples had microscopically mixed life stages with varying copies of the 18S rRNA gene and thus the assay readout is reported in terms of gc/mL. Based on this and the above experiments, 20 gc/mL was set as the lower limit of detection to meet positive reporting criteria, but all raw data are shown in the Results.

For quantification of *P. falciparum* parasitemia in VAC069E, a similar qPCR method was used. DNA was extracted from 0.4 mL whole EDTA blood using the QIAamp DSP DNA Blood Mini Kit (Qiagen), with a 100 μL elution in AE buffer. Additionally, aliquots of baseline samples taken

within 2 days pre-CHMI were spiked with a known concentration of positive control DNA to check there was no presence of PCR inhibitors in participants' blood prior to CHMI.

Following DNA extraction, a standard Taqman absolute quantitation was used against a standard curve to amplify a 133 bp PCR product from the multi-copy, highly conserved 18S ribosomal RNA genes of Pf. qPCR used the following adapted oligonucleotide primers and probe[9]: 18s forward primer 5'-GTA ATT GGA ATG ATA GGA ATT TAC AAG GT-3', 18s reverse primer 5'-TCA ACT ACG AAC GTT TTA ACT GCA AC-3', and FAM labelled probe sequence 5'-FAM- AAC AAT TGG AGG GCA AG-NFQ-MGB-3', with TaqMan Universal PCR master mix II (with Amperase UNG), and was run on an QuantStudio3 machine with DA2 software (Applied Biosystems). Default Universal qPCR (target FAM-NFQ-MGB) and quality control (QC) settings were used. The standard curve utilized known quantities of cultured Pf material, as previously described[6].

For QC purposes, qPCR samples were re-tested if replicates included a mixture of positive and negative (in terms of amplification) results with one or more positive results >100 gc/mL or if the % CV of any results were high outliers. All 'passed' data following the quality control steps above, including any 0 values, were used to generate the final mean qPCR result for each time-point.

Thick blood film microscopy

Blood film microscopy was used in phases VAC069A and VAC069B only. Collection of blood, preparation of thick films and slide reading were performed according to Jenner Institute Standard Operating Procedure (SOP) ML009. Slides were prepared using Field's stain A and then Field's stain B. 200 fields at high power (1000x) were read. Visualization of two or more parasites in 200 high power fields constituted a positive result. For internal quality control, all slides were read separately by two microscopists, with a third read if results were discordant (one negative and one positive report).

Diagnostic algorithms used in VAC069 study

Algorithm for diagnosis and initiation of treatment VAC069A and B:

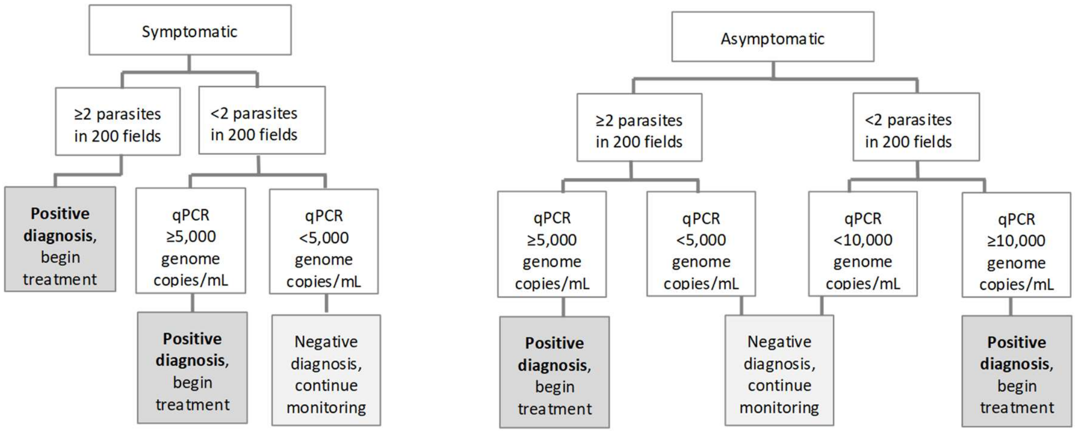

Algorithm for diagnosis and initiation of treatment in VAC069C to E:

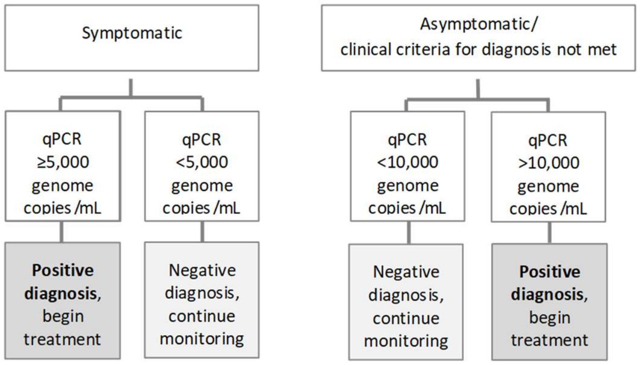

Modelling of parasite multiplication rate

A qPCR-derived parasite multiplication rate (PMR) was modelled based on previously described methodology with modifications [5,8]. The arithmetic mean of three replicate qPCR results obtained for each individual at each time-point was used for model-fitting. Negative individual replicates were assigned a value of 0 gc/mL for the purposes of calculating the arithmetic mean of triplicates (where at least one of the three readings was positive). All qPCR data points which, based upon the mean of the three replicates, were greater than 5 gc/mL were used for modelling. Any values ranging from 1-5 gc/mL and any data point that was negative but preceded a positive data point were replaced with a value of 5 gc/mL; otherwise negative data points occurring after any positive data point but not preceding a positive data point were treated as 0 gc/mL. The time interval

between the morning and evening bleeds used for qPCR monitoring was set as 0.37 days. PMR per 48 h was then calculated using a linear model fitted to log_{10}-transformed qPCR data.

To allow direct comparison across the different cohorts for *P. vivax* CHMI, data from timepoints in CHMIs conducted in September 2019 and May 2021, which would not have been available if using the visit schedule for the *P. vivax* CHMI in October 2021, were removed prior to model-fitting.

Supplementary Figures

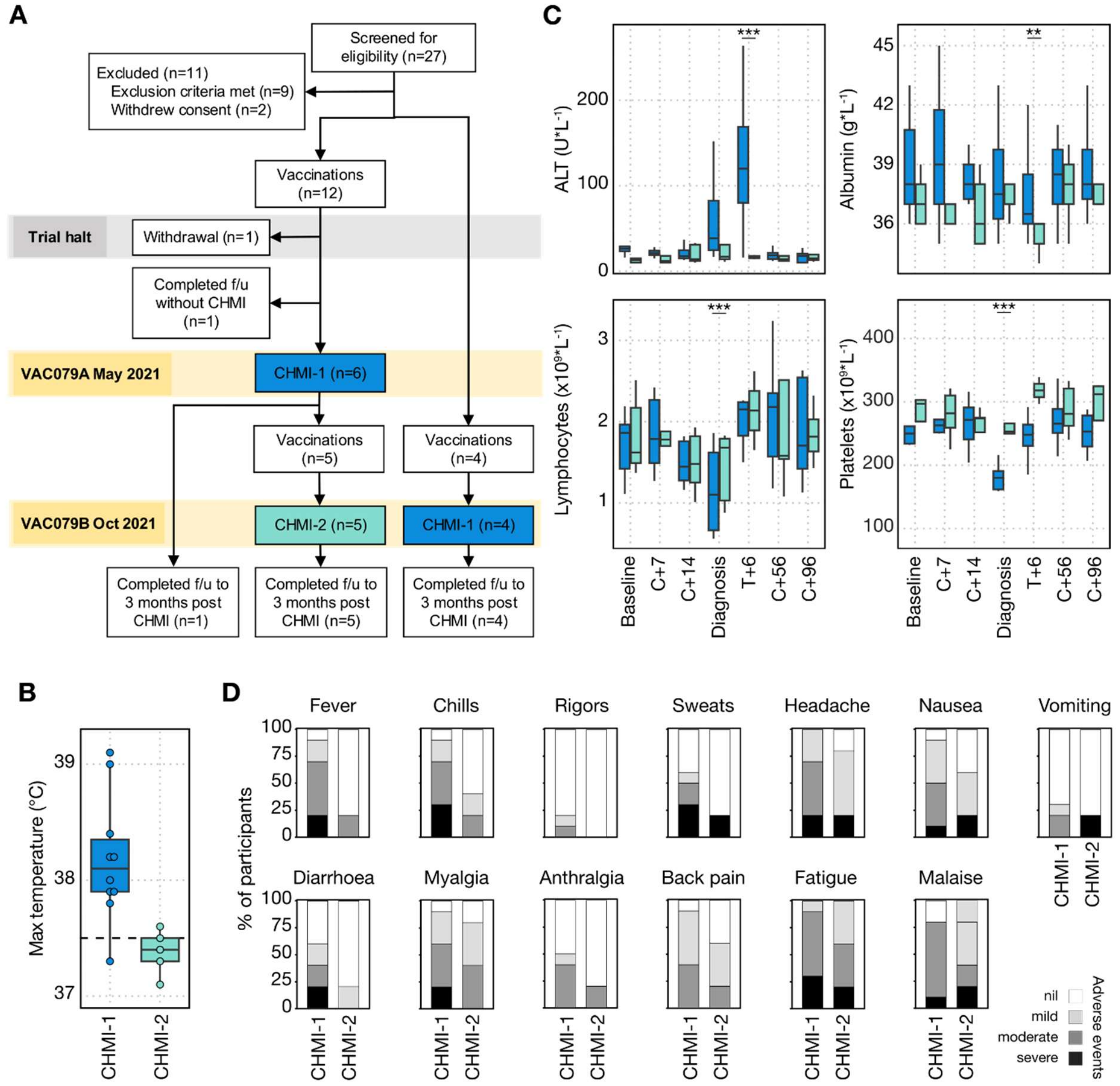

Figure S1. Primary and secondary *P. vivax* CHMI in the VAC079 study.
(A) VAC079 tested the efficacy of a *P. vivax* PvDBPII protein/adjuvant vaccine. 10 participants completed 3 vaccinations prior to undergoing primary *P. vivax* CHMI (CHMI-1) at 2 to 4 weeks after their third vaccination. Five participants received a fourth vaccination, followed by secondary homologous *P. vivax* CHMI (CHMI-2). **(B)** Maximum recorded core temperature during primary and secondary CHMI ($p = 0.1$ by Wilcoxon signed-rank test). **(C)** Alanine aminotransferase (ALT), serum albumin, lymphocyte and platelet counts were measured in peripheral blood during primary and secondary CHMI at baseline (1 or 2 days before challenge); 7 and 14 days after challenge (C+7 and C+14); on the day of diagnosis; 6 days after treatment (T+6); and 56 (C+56) and 96 days (C+96) after challenge. Statistically significant differences between CHMI-1 and CHMI-2 were identified at each time-point by using mixed-effects modelling and

linear regression (* $p < 0.05$ and *** $p < 0.001$). Box and whisker plots show median and IQR (whiskers represent 1.5x IQR). (D) Clinical signs and symptoms of malaria in participants undergoing primary and secondary CHMI in the VAC079 study. Data show the maximum severity of each solicited adverse event (AE), consisting of 13 malaria symptoms, reported by an individual in the 48 hours before and after diagnosis as a proportion of the total number of participants undergoing CHMI. In (B-D) n = 10 (primary *P. vivax* CHMI) and n = 5 (secondary *P. vivax* CHMI).

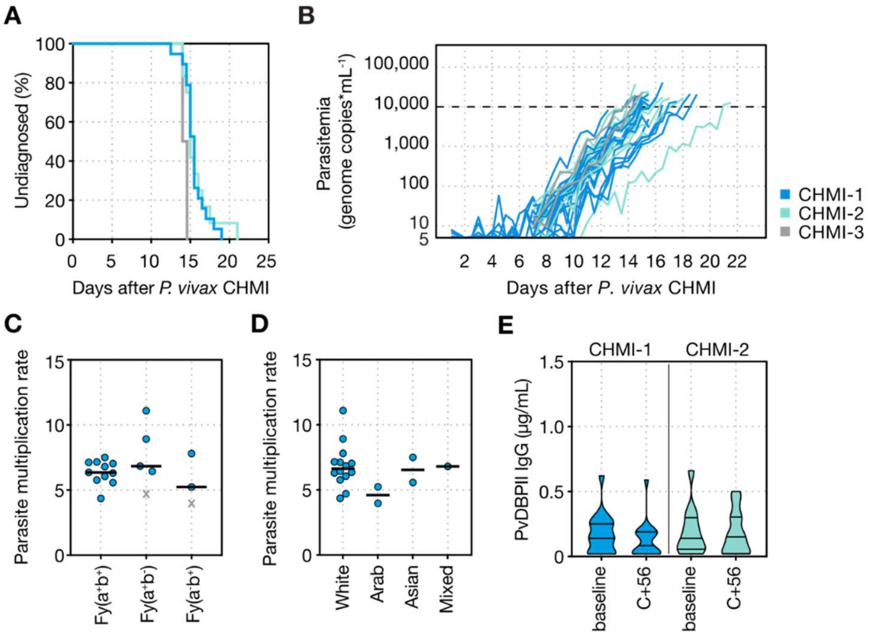

Figure S2. Parasite growth during *P. vivax* CHMI in the VAC069 study.
(A) Kaplan-Meier plot showing the time to malaria diagnosis in the VAC069 study. Median time to diagnosis was 15.5 days during primary (CHMI-1) and 15.0 days during secondary *P. vivax* CHMI (CHMI-2); no significant difference by log-rank test (p = 0.8). Median time to diagnosis was 14.25 days during tertiary CHMI (CHMI-3) **(B)** Parasitaemia was measured up to twice daily by qPCR, note the two participants during tertiary *P. vivax* (CHMI-3, shown in grey). The dashed line indicates the treatment threshold of 10,000 gc ml^{-1}. **(C)** Parasite multiplication rate (fold-change per 48 hours) during primary CHMI is grouped by Duffy phenotype; no significant difference between group medians by Kruskal-Wallis test. The two individuals designated by a cross were heterozygous for the erythroid silent FY*B allele on Duffy genotyping. **(D)** Parasite multiplication rate (fold-change per 48 hours) during primary CHMI is grouped by participant ethnicity. **(E)** Violin plot shows anti-PvDBPII IgG serum concentrations as measured by ELISA at 1 or 2 days before challenge (baseline) and 56 days after challenge (C+56) during primary (CHMI-1) and secondary *P. vivax* CHMI (CHMI-2). All responses are below the limit of quantification (1 µg ml^{-1}). In (A-D) n = 19 (primary *P. vivax* CHMI) and n = 12 (secondary *P. vivax* CHMI).

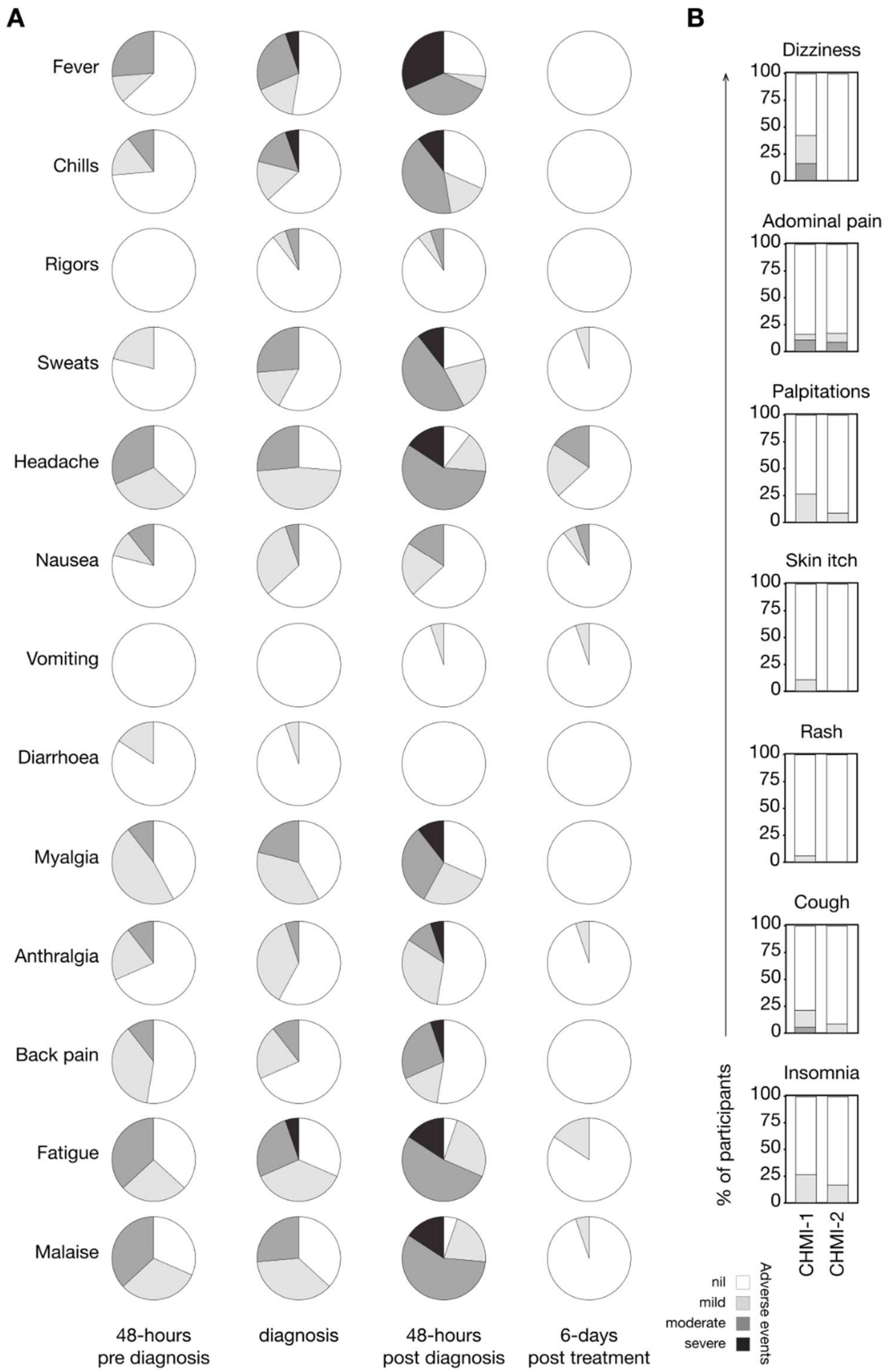

Figure S3. Solicited adverse events during *P. vivax* CHMI in the VAC069 study.
(A) Clinical signs and symptoms of malaria in participants undergoing primary *P. vivax* CHMI in the VAC069 study. Data show the maximum severity of each solicited adverse event (AE), consisting of 13 malaria symptoms, as reported by an individual in the 48 hours before diagnosis; at diagnosis; the 48 hours after diagnosis; and 6 days after starting antimalarials. All data are shown as a proportion of the total number of participants undergoing CHMI (n = 19). **(B)** Solicited

adverse events (AE) associated with antimalarial treatment during primary (CHMI-1, n = 19) and secondary (CHMI-2, n = 12) *P. vivax* CHMI. The maximum severity of each AE is shown as a proportion of the total number of participants undergoing CHMI.

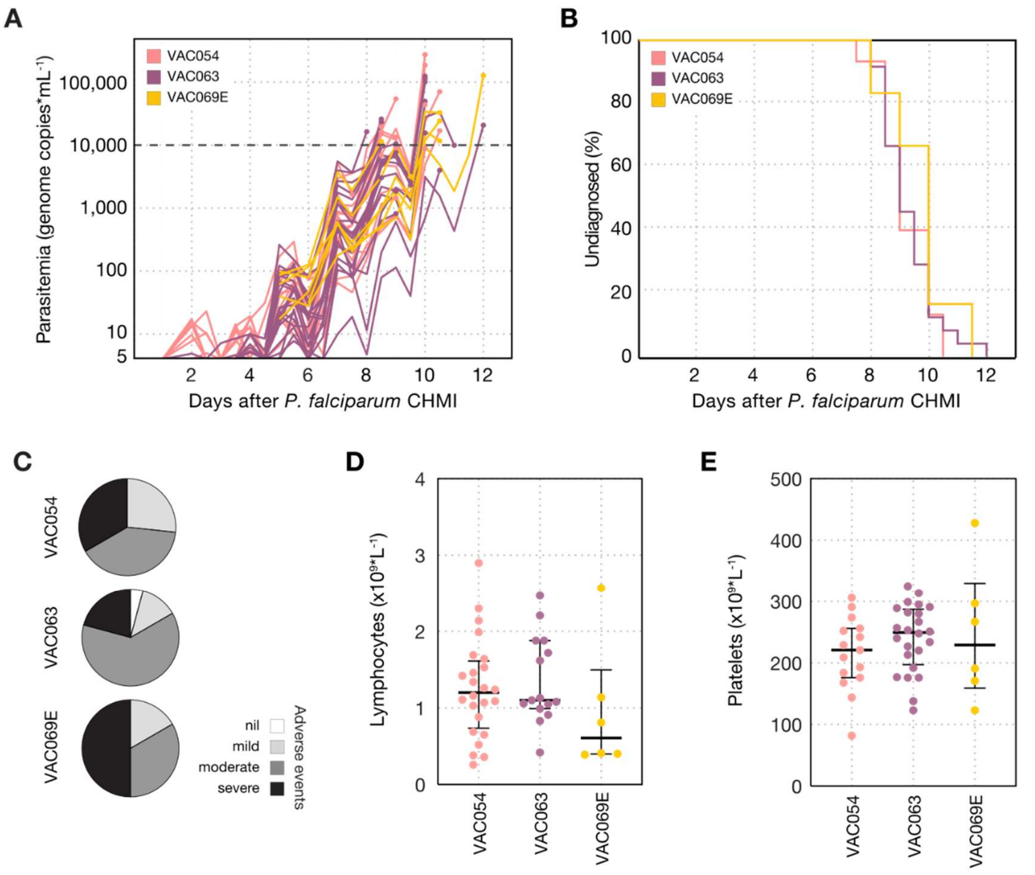

Figure S4. Parasitaemia and solicited adverse events during heterologous rechallenge are similar to primary *P. falciparum* CHMI

(A) Parasitaemia was measured up to twice daily by qPCR during heterologous rechallenge with *P. falciparum* (VAC069E) and compared to primary CHMI in malaria-naïve participants in two of our previous CHMI studies (VAC054 and VAC063), in which participants were inoculated with red bloods cells infected with the same 3D7 clone of *P. falciparum*[21,33,34]. The diagnostic criteria used in VAC054 and the first cohort of VAC063 (VAC063A) combined microscopy and qPCR, whereas in VAC063B-C and VAC069E only qPCR was used. Despite this no significant differences in the parasitaemia at diagnosis were seen across all CHMIs. The dashed line indicates the treatment threshold for VAC069E. (B) Kaplan-Meier plot showing the time to malaria diagnosis in VAC069E compared to primary CHMI in VAC054 and VAC063. Median time to diagnosis was 10 days in VAC069E and 9 days in both VAC054 and VAC063; no significant difference by log-rank test (p = 0.6). (C) Maximum severity of any solicited adverse event (AE), consisting of 13 malaria symptoms, reported by an individual in the 48 hours before and after diagnosis during primary *P. falciparum* CHMI in VAC054, VAC063 compared to VAC069E. Data are shown as a proportion of the total number of participants undergoing each CHMI. (D) Lymphocyte and (E) platelet counts were measured in peripheral blood on the day of diagnosis in VAC069E and compared to primary *P. falciparum* CHMI in VAC054 and VAC063. No significant differences between groups by Kruskal-

Wallis test (lymphocytes $p = 0.3$, platelets $p = 0.5$). Median and IQR shown. In (A-E) n = 15 (VAC054), n = 24 (VAC063), n = 6 (VAC069E).

Supplementary Tables

Table S1: Demographics of study participants.

Pv CHMI-1 indicates participants who underwent primary controlled human malaria infection with *P. vivax*; *Pv* CHMI-2 secondary homologous *P. vivax* CHMI; *Pv* CHMI-3 tertiary homologous *P. vivax* CHMI; *Pf* CHMI repeat heterologous CHMI with *P. falciparum*.

		VAC069				VAC079	
		Pv CHMI-1	*Pv* CHMI-2	*Pv* CHMI-3	*Pf* CHMI	*Pv* CHMI-1	*Pv* CHMI-2
No. of participants		19	12	2	6	10	5
Female Sex, no. (%)		9 (47.4)	6 (50.0)	0	4 (66.7)	8 (80.0)	5 (100.0)
Age (yr)	Median	26	27	30	33	37	42
	Range	21-48	22-48	29-31	22-49	22-45	23-46
Body Mass Index (kg/m^2)	Median	23.6	24.1	28.7	25.1	28.9	27.6
	Range	17.5-33.5	18.1-32.6	24.8-32.6	18.9-33.5	23.5-40.2	23.5-35.9
Ethnicity, no. (%)	White-British	8 (42.1)	5 (41.7)	1 (50.0)	4 (66.7)	9 (90.0)	4 (80.0)
	White-Other	6 (31.6)	4 (33.3)	1 (50.0)	2 (33.3)	1 (10.0)	1 (20.0)
	Asian	2 (10.5)	1 (8.33)	0	0	0	0
	Arab	2 (10.5)	1 (8.33)	0	0	0	0
	Mixed	1 (5.3)	1 (8.33)	0	0	0	0
Duffy Phenotype, no. (%)	Fy(a+b-)	5 (26.3)	4 (33.3)	1 (50.0)	0	1 (10.0)	0
	Fy(a-b+)	3 (10.5)	2 (16.7)	0	1 (16.7)	3 (30.0)	2 (40.0)
	Fy(a+b+)	11 (63.2)	6 (50.0)	1 (50.0)	5 (83.3)	6 (60.0)	3 (60.0)

Table S2: Unsolicited adverse events.

Unsolicited adverse events (AEs) deemed possibly, probably or definitely related to CHMI in each study. Number of episodes of unsolicited AE are listed by MEDDRA System Organ Class and Preferred Term and maximal severity grade.

Table S2A Unsolicited AEs in the VAC069 study during *P. vivax* CHMI

Unsolicited adverse event		Primary CHMI (n=19)		Secondary CHMI (n=12)		Tertiary CHMI (n=2)		Total
System Order Class	Preferred Term	Grade 1	Grade 2	Grade 1	Grade 2	Grade 1	Grade 2	
Blood and lymphatic system disorders	Lymphadenopathy	1						1
Gastrointestinal disorders	Constipation	1						1
	Tooth infection				1			1
	Dyspepsia					1		1
Infections and infestations	Tonsillitis				1			1
Metabolism and nutrition disorders	Decreased appetite	2	1					3
Musculoskeletal and connective tissue disorders	Myalgia	1						1
	Musculoskeletal stiffness	1						1
Nervous system disorders	Paraesthesia	1						1
Psychiatric disorders	Stress	1						1
Respiratory, thoracic and mediastinal disorders	Dry throat	1						1
	Oropharyngeal pain	1						1
Skin and subcutaneous tissue disorders	Pruritus	1						1

Table S2B Unsolicited AEs in the VAC079 study during *P. vivax* CHMI

Unsolicited adverse event		Primary CHMI (n=10)		Secondary CHMI (n=5)		Total
System Order Class	Preferred Term	Grade 1	Grade 2	Grade 1	Grade 2	
Gastrointestinal disorders	Abdominal pain upper				1	1
General disorders and administration site conditions	Chest pain			1		1
Metabolism and nutrition disorders	Decreased appetite	1				1
Musculoskeletal and connective tissue disorders	Musculoskeletal stiffness	1				1
	Back pain	1				1
Nervous system disorders	Paraesthesia	1				1
Renal and urinary disorders	Chromaturia	2				2
Skin and subcutaneous tissue disorders	Rash	1				1

Table S2C Unsolicited AEs in the VAC069 study during *P. falciparum* CHMI

Unsolicited adverse event		*P. falciparum* CHMI (n=6)	Total
System Order Class	Preferred Term	Grade 1	
Infections and infestations	Nasopharyngitis	1	1
Skin and subcutaneous tissue disorders	Rash maculo-papular	1	1

Table S3: Laboratory adverse events.

Laboratory adverse events (AEs) deemed at least possibly related to CHMI in each study. Number of episodes of AE and maximal severity of AE during episode listed.

Table S3A Laboratory AEs in the VAC069 study during *P. vivax* CHMI

Laboratory abnormality		**Primary CHMI** (n=19)			**Secondary CHMI** (n=12)			**Tertiary CHMI** (n=2)			**Total**
		Grade 1	Grade 2	Grade 3	Grade 1	Grade 2	Grade 3	Grade 1	Grade 2	Grade 3	
Haematology	Leucopaenia	9	5		1						15
	Lymphopaenia	5	7	5	1	1		1			20
	Neutropaenia	5	1		1			1			8
	Anaemia	4	2		5	1					12
	Thrombocytopaenia	3	4	2	1						10
Biochemistry	Raised ALT	5	4	3	1				1		14
	Hyperbilirubinaemia	1									1
	Hypoalbuminaemia				1						1
	Raised urea				1						1
	Hypokalaemia	2			1			1			4

Table S3B Laboratory AEs in the VAC079 study during *P. vivax* CHMI

Laboratory abnormality		Primary CHMI (n=10)			Secondary CHMI (n=5)			Total
		Grade 1	Grade 2	Grade 3	Grade 1	Grade 2	Grade 3	
Haematology	Leucoytosis				1			1
	Leucopaenia	1	4					5
	Lymphopaenia		5	2				7
	Neutropaenia	3	1					4
	Anaemia	5			3			8
	Thrombocytopaenia			1				1
Biochemistry	Raised ALT	1	5	1				7
	Raised ALP	2						2

Table S3C Laboratory AEs in the VAC069 study during *P. falciparum* CHMI

Laboratory abnormality		*P. falciparum* CHMI (n=6)			Total
		Grade 1	Grade 2	Grade 3	
Haematology	Leucopaenia	2	1		3
	Lymphopaenia		1	3	4
	Neutropaenia	3			3
	Anaemia	1			1
	Thrombocytopaenia	1	1		2
Biochemistry	Raised creatinine	1			1
	Raised ALT	2		1	3
	Hyperbilirubinaemia	1			1

Table S4: Malaria qPCR data (gc/mL) for each CHMI

Malaria qPCR data used in PMR modelling are shown. The top row represents day (D) of follow-up visit post blood-stage CHMI. DoD indicates the timepoint at which malaria diagnostic criteria were reached. Treatment in some participants was started half a day after reaching malaria diagnostic criteria. qPCR data shown for samples taken prior to starting treatment. qPCR negative values for all three triplicate readings in the assay are indicated by 'N'. Squares highlighted in gray indicate negative or < 20 gc/mL which is below minimum positive reporting criteria and these datapoints were removed for PMR modelling. Datapoints which would not have been taken during CHMI in October 2021 due to changes in protocol were removed for PMR modelling and are not shown. Blacked out boxes indicate the timepoints after a participant commenced antimalarial treatment. In VAC069A, inoculum dose was either 1 vial, 1:5 dilution of 1 vial or 1:20 dilution of 1 vial to determine the best dose to take forward for subsequent CHMIs. In VAC069B to D a 1:10 dilution of 1 vial of inoculum was used.

Table S4A Malaria qPCR during VAC069A in Jan 2019

No of CHMI	DoD	D7	D8	D9	D10	D10.5	D11	D11.5	D12	D12.5	D13	D13.5	D14	D14.5	D15	D15.5	D16	D16.5	D17
1 (1 vial)	15.5	17	17	58	67		301		294		956		2251	417	5035	3779			
1 (1 vial)	12.5	78	60	508	1060	2060	3132	2640	5393	11259									
1 (1:5 vial)	15	5	18	72	239		519		972		2666	2999	4700	1538	17795	14843			
1 (1:5 vial)	15	6	21	33	8		266		459		1331		2632	1056	8687	7112			
1 (1:20 vial)	15.5	N	5	32	57		406		616		1431	1253	3054	1444	9597	8134	15768		
1 (1:20 vial)	16.5	N	5	15	8		53		76		239		717		1479	1438	2593	9668	7894

Table S4B Malaria qPCR during VAC069B in Sept 2019

No of CHMI	DoD	D7	D8	D9	D10	D10.5	D11	D11.5	D12	D12.5	D13	D13.5	D14	D14.5	D15	D15.5	D16	D16.5	D17
1	15.5	N	5	45	104		209		270		1379	1391	2244	3670	8921	9574	16345		
1	14.5	5	9	72		5	265		778		3365	2835	4283	17392	19589				
2	21	N	N	N	N	N	N	5	32		70		119		235		217		995
2	15.5	5	20	41	17		113		351		1371	492	2299	3374	8679	8489	17449		
2	14	11	68	86	145		553		1405	2601	5691	4021	11364	17496					

No of CHMI	DoD	D17.5	D18	D18.5	D19	D19.5	D20	D20.5	D21	D21.5
1	15.5									
1	14.5									
2	21		1180	1033	3119	3026	3950	2713	11194	12522
2	15.5									
2	14									

Table S4C Malaria qPCR during VAC069C in May 2021

No of CHMI	DoD	D7	D8	D9	D10	D11	D11.5	D12	D12.5	D13	D13.5	D14	D14.5	D15	D15.5	D16	D16.5	D17	D17.5	D18	D18.5	D19
1	15.5	N	5	74	220	316		408		1678	1315	3625	6183	8674	9095							
1	18	10	5	8	N	125		88		291		814	1252	1652	1805	2152	6550	7261	6161	12050	20348	
1	19	N	5	26	37	98		62		268		298		1111	1262	1617	2121	6005	6395	4758	9432	19781
1	14	32	35	108	182	1059	748	1121	2572	8025	7805	14766	18983									
1	16	11	5	47	74	311		298		1669	1544	3316	6509	8709	9292	15568	39496					
1	14.5	N	5	50	96	370		754		2641	2335	4847	12160	7238								
1	15	N	5	113	183	485		556		3001	2476	5926	7904	16068	20776							
2	15	5	35	70	93	538		870		3906	3531	7560	9434	22138	24051							
2	15	25	54	82	103	447		450		2626	2679	4126	6192	13673	14529							
3	14.5	16	9	92	122	331		480		2780	2934	6908	13567	22850								

Table S4D Malaria qPCR during VAC069D in Oct 2021

No of CHMI	DoD	D7	D8	D9	D10	D10.5	D11	D11.5	D12	D12.5	D13	D13.5	D14	D14.5	D15	D15.5	D16	D16.5	D17	D17.5	D18
1	15	8	28	66	124		604		806		2823	2487	3759	3551	14373	10759					
1	15	17	17	71	119		1021	804	1617	1727	3405	4145	5742	6828	17155	13910					
1	17	5	9	5	7		73		46		510		501		1735	1792	3600	4258	12302	13366	
1	15.5	5	61	53	73		401		767		1552	1096	2597	2245	5737	3057					
2	16	16	16	92	24		504		393		1536	1595	1702	3079	7646	6541	12156	16931			
2	16.5	9	5	31	84		195		303		998		1180	2409	3554	3209	6561	13796	16362		
2	17.5	5	19	77	58		384		310		565		1056	839	2261	1844	3977	6472	9923	12001	16503
2	14	8	60	200	271		1631	996	2660	5989	8523	8670	12188	36683							
2	15	17	18	86	139		732		861		2866	2681	3215	4970	12803	9949					
2	14.5	5	59	56	205		651		1436	1627	3081	2283	5242	10170	16545						
2	15	9	69	87	138		735		1096	1349	3599	3153	6065	9292	23263	22349					
3	14	5	38	232	213		1447	1516	2393	3266	7133	8062	12560	19858							

Table S4E Malaria qPCR during VAC069E in Oct 2022

No of CHMI	DoD	D5	D6	D7	D7.5	D8	D8.5	D9	D9.5	D10	D10.5	D11	D11.5	D12
3	C + 10	N	N	157	83	171	570	1752	313	16701	11670			
3	C + 9	21	10	518	326	1557	3247	10161						
3	C + 8.5	N	19	965	741	3780	11311							
2	C + 10	4	N	139	39	694	1060	1951	953	11790	24274			
2	C + 10	N	N	164	153	427	1027	3116	1472	33908	32777			
2	C + 12	4	5	30		178		695		9563	4890	1854	7059	127550

Table S5: Merozoite antigens tested in multiplex immunoassay

Gene ID	Addgene ID	Name	Description	Domain Boundary	CD4 tag (20.4kDa) Present	Mol.wt. (kDa) Including Tag	Bead Region	Working stock concentration nml^{-1}
**KMZ83376.1**	-	PvEBP	Plasmodium vivax Erythrocyte Binding Protein	M1-716P	No	95	12	$0.7x10^6$
**PVX_113775**	#68516	Pv12	Plasmodium vivax 6-cysteine protein	F24-A339	No	38.6	14	$0.72x10^6$
**PVP01_0934200**	-	PvAMA1	Plasmodium vivax Apical Merozoite Antigen 1	K25-479E	No	52.7	15	$0.74x10^6$
**PVX_099980**	#68505	PvMSP1	Plasmodium vivax Merozoite Surface Protein 1	E20-P1721	No	215	18	$0.59x10^6$
**PVX_088910**	#68522	PvGAMA	Plasmodium vivax GPI-anchored micronemal antigen, putative	L21-S749	No	85	21	$1.32x10^6$
**PVP01_0000100**	-	PvTRAg25	Plasmodium vivax Tryptophan Rich Antigen 25	K62-L693	No	74.3	22	$0.75x10^6$
**PVX_110810**	#68528	PvDBP	Plasmodium vivax Duffy binding protein	V23-T1008	Yes	142	25	$0.9x10^6$
**Cd4**	-	CD4	Cluster of differentiation 4	S1-183N	-	20.4	20	$0.5x10^6$

Supplementary References

1. Hostetler, J. B. *et al.* A Library of Plasmodium vivax Recombinant Merozoite Proteins Reveals New Vaccine Candidates and Protein-Protein Interactions. *PLoS Negl Trop Dis* **9**, e0004264 (2015).
2. Petersen, T. N., Brunak, S., von Heijne, G. & Nielsen, H. SignalP 4.0: discriminating signal peptides from transmembrane regions. *Nat Methods* **8**, 785–786 (2011).
3. Sonnhammer, E. L., von Heijne, G. & Krogh, A. A hidden Markov model for predicting transmembrane helices in protein sequences. *Proc Int Conf Intell Syst Mol Biol* **6**, 175–182 (1998).
4. Payne, R. O. *et al.* Human vaccination against Plasmodium vivax Duffy-binding protein induces strain-transcending antibodies. *JCI Insight* **2**, e93683 (2017).
5. Minassian, A. M. *et al.* Controlled human malaria infection with a clone of *Plasmodium vivax* with high-quality genome assembly. *JCI Insight* **6**, (2021).
6. Minassian, A. M. *et al.* Reduced blood-stage malaria growth and immune correlates in humans following RH5 vaccination. *Med* **2**, 701-719.e19 (2021).
7. McCarthy, J. S. *et al.* Experimentally Induced Blood-Stage Plasmodium vivax Infection in Healthy Volunteers. *The Journal of Infectious Diseases* **208**, 1688–1694 (2013).
8. Douglas, A. D. *et al.* Comparison of Modeling Methods to Determine Liver-to-blood Inocula and Parasite Multiplication Rates During Controlled Human Malaria Infection. *The Journal of Infectious Diseases* **208**, 340–345 (2013).
9. Hermsen, C. C. *et al.* Detection of *Plasmodium falciparum* malaria parasites in vivo by real-time quantitative PCR. *Molecular and Biochemical Parasitology* **118**, 247–251 (2001).

VAC069: A clinical study to assess the safety and feasibility of controlled blood-stage *Plasmodium vivax* human malaria infection through experimental inoculation of cryopreserved infected erythrocytes in healthy malaria-naïve UK adults

Study Reference: VAC069

Protocol Version: 8.0

Date: 23rd June 2022

Chief Investigator: Angela M. Minassian

Sponsor: University of Oxford

MODIFICATION HISTORY

Version	Date	Author(s)	Modifications
1.0	19 September 2018	Yrene Themistocleous<br>Simon Draper<br>Angela Minassian	Created
1.1	10th December 2018	Yrene Themistocleous	Minor clarification regarding procedure for preparation of inoculum , referencing the standard operating procedure
2.0	12th February 2019	Yrene Themistocleous<br>Thomas Rawlinson<br>Angela Minassian	Substantial amendment.<br>Number of volunteers increased from 6 to 25<br>Number of challenges increased from 3 to 5<br>Amendments to the schedule of clinic visits and blood tests<br>Inclusion of mosquito transmission studies
3.0	10th September 2019	Susanne Hodgson<br>Yrene Themistocleous	Substantial amendment.<br>Addition of testing for blood borne viruses at C+90 visits
4.0	23rd October 2019	Susanne Hodgson<br>Angela Minassian	Substantial amendment<br>Removal of microscopy from diagnostic criteria for VAC069C-E and from study endpoints<br>Updates to schedule of visits and bleeds, simplification of bleed tables and reduction in total blood draw volume for VAC069C-E<br>Change in exclusion criteria regarding prior use of immunoglobulin & GP response<br>Removal of AEs of special interest section<br>Modification to collection of adverse event data<br>Modification to timing of observations on day of challenge procedures<br>Addition of potential for pre-screening via telephone<br>Potential for use of alternative antiemetic to cyclizine.

			Ranges added for Group sizes<br>Local safety monitor takes on role previously taken by local safety committee<br>Consent form only required to be completed once at screening (and not routinely again prior to each challenge)<br>Update of bleed tables to show that 4ml rather than 5mls was collected for HLA and haemoglobinopathy testing for VAC069A&B.
5.0	19th August 2020	Yrene Themistocleous<br>Mimi Hou<br>Angela Minassian	Corrections to blood draw volumes for VAC069C-F<br>Addition of VAC069F and additional secondary and tertiary challenges in phases VAC069E and VAC069F<br>Adjustment of study group sizes for VAC069C-F<br>Extension of time window between primary and subsequent challenges<br>Extension of time window for follow-up study visits<br>Changes to trial procedures to account for possibility of COVID-19 infection during CHMI. Addition of COVID-19 PCR swab test prior to challenge and on day of malaria diagnosis. Guidance on testing for COVID-19 if fever post challenge.<br>Added option of using Malarone as first line anti-malaria treatment<br>Correction of typographical errors. Addition of relevant references. Clarification of study procedures in text: G6PD, DARC & haemoglobinopathy screen are done by NHS labs only; added description of T+3 and T+9 visits within text; added reasoning for selecting 1:10 dose of blood inoculum for all subsequent malaria challenges after VAC069A.
6.0	4th March 2021	Mimi Hou<br>Jee-Sun Cho	Addition of Wellcome Trust as Funder of VAC069C.<br>Addition of exclusion criteria on concomitant COVID-19 vaccination around time of CHMI

			Updated section on Conduct of CHMI in the context of COVID-19 pandemic.<br>Correction of total blood volumes in VAC069C-F schedule of attendance tables.<br>Addition of DARC to schedule of attendance table.<br>Change of C-1 visit to C-2.<br>Increased number of back-up volunteers that can be recruited into each group.<br>Correction in section 6.4 that there is no specific timeframe that observations are taken pre-challenge.
7.0	28th July 2021	Mimi Hou	For phase VAC069D onwards:<br>Post-challenge follow-up visits changed to once a day until parasite count reaches >1000 genome copies/ml, then to continue twice a day visits until diagnosis. Latest day of treatment reduced to C+21.<br>Removal of T+2 post malaria treatment visit. Reduction in number of observed doses of antimalarial medication to two observed doses.<br>Updated compensation table<br>Addition of reticulocyte count to FBC taken at C-2 visit.<br>Removal of need to collect unused medications after completion of challenge. Dose of lumefantrine corrected in section 6.4.4<br>Clarification that only weight is re-measured at C-2 visit.<br>Changed Senior Laboratory Investigator address. Addition of New Biochemistry Building as location for processing of research bloods.
8.0	23rd June 2022	Mimi Hou<br>Angela Minassian	Phase VAC069E changed to CHMI with P. falciparum instead of P. vivax. Addition to section on study rationale, details on P. falciparum inoculum to be used, secondary objective, immunological blood tests and study visits for VAC069E.

			Clarification of immunological tests on blood samples and assays to be used to include both vivax and falciparum CHMIs. Cell phenotyping by mass cytometry added to immunological tests.<br>Addition of red cell alloantibody testing pre and post CHMI on either stored serum or from whole blood.<br>Removal of phase VAC069F<br>Increased time window for re-screening visit for VAC069E.<br>Change to exclusion criteria on day of CHMI – Covid infection now an exclusion criteria if within 7 days.<br>Covid testing pre-CHMI can now be performed by lateral flow test or PCR test. Requirement for volunteers to undergo Covid test on day of malaria diagnosis removed.<br>Samples will now be stored long term under University of Oxford's HTA license at Department of Biochemistry instead of Oxford Vaccine Centre Biobank.<br>Organisation name changes: sponsor name change from Clinical Trials and Research Governance to Research Governance Ethics and Assurance; Public Health England changed to UK Health Security Agency

A clinical study to assess the safety and feasibility of controlled blood-stage *Plasmodium vivax* human malaria infection through experimental inoculation of cryopreserved infected erythrocytes in healthy malaria-naïve UK adults

Study Code: VAC069

Chief Investigator	Dr Angela M Minassian<br>Centre for Clinical Vaccinology and Tropical Medicine,<br>Jenner Institute, University of Oxford,<br>Churchill Hospital, Old Road, Headington,<br>Oxford, OX3 7LE<br>Email: angela.minassian@ndm.ox.ac.uk
Principal Investigator	Prof Simon J Draper<br>New Biochemistry Building,<br>Department of Biochemistry,<br>South Parks Road<br>Oxford, OX1 3QU<br>Email: simon.draper@bioch.ox.ac.uk
Trial sites	Jenner Institute, University of Oxford<br>Comprising:<br>Centre for Clinical Vaccinology & Tropical Medicine<br>Churchill Hospital, Old Road, Headington,<br>Oxford, OX3 7LE<br>And<br>Old Road Campus Research Building,<br>Roosevelt Drive, Headington,<br>Oxford, OX3 7DQ<br>PI at Site: Dr Angela M Minassian
Sponsoring Institution	University of Oxford

Research Governance Ethics and Assurance,
Research Services
University of Oxford
Joint Research Office
Boundary Brook House
Churchill Drive
Headington
OX3 7GB
Tel: 01865 616480
Email: ctrg@admin.ox.ac.uk

External Monitor	Research Governance, Ethics and Assurance (RGEA)<br>Research Services<br>University of Oxford<br>Joint Research Office<br>Boundary Brook House<br>Churchill Drive<br>Headington<br>OX3 7GB<br>Tel: 01865 616480
Local Safety Monitor	Prof Brian Angus<br>Centre for Clinical Vaccinology and Tropical Medicine,<br>Churchill Hospital, Old Road, Headington,<br>Oxford, OX3 7LE<br>Tel: 01865 553 20289<br>Email: brian.angus@ndm.ox.ac.uk

Confidentiality Statement

This document contains confidential information that must not be disclosed to anyone other than the Sponsor, the Investigator Team, and members of the Independent Ethics Committee. This information cannot be used for any purpose other than the evaluation or conduct of the clinical investigation without the prior written consent of Dr Angela M. Minassian.

Investigator Agreement

"I have read this protocol and agree to abide by all provisions set forth therein.

I agree to comply with the principles of the International Conference on Harmonisation Tripartite Guideline on Good Clinical Practice."

Dr Angela M. Minassian		23/06/2022
Chief Investigator	Investigator Signature	Date

Conflict of Interest

1. "According to the Declaration of Helsinki, 2008, I have read this protocol, and declare no/~~the following~~ (delete as appropriate) conflict of interest"

Dr Angela M. Minassian		23/06/2022
Chief Investigator	Investigator Signature	Date

Details:____

2. "According to the Declaration of Helsinki, 2008, I have read this protocol, and declare no/~~the following~~ (delete as appropriate) conflict of interest"

Prof Simon J. Draper		23/06/2022
Principal Investigator	Investigator Signature	Date

Details:____

TABLE OF CONTENTS

Modification History 2

1 Synopsis 15

2 Abbreviations 17

Background & Rationale 19

3.1 Impact of *P. vivax* malaria and the need for a vaccine 19

3.2 Lifecycle of the *Plasmodium vivax* malaria parasite 20

3.3. Challenges and directions in *P. vivax* vaccine development 21

3.4 Rationale for the development of a blood-stage challenge model 22

3.5 Study rationale 25

3.6 Rationale for re-challenge with *P. falciparum* 25

4 Objectives 27

5 Controlled human malaria infection studies 28

5.1 Microbial challenge studies of human volunteers 28

5.2 Controlled human malaria infection with *Plasmodium falciparum* 28

5.3 Controlled human malaria infection with *Plasmodium vivax* 28

5.4 *P. vivax* sporozoite challenge in the modern era 29

5.5 Modern blood-stage controlled human *P. vivax* infection 33

5.6 Re-challenge in controlled human malaria infection 34

5.6.1 Safety of re-challenge in controlled human malaria infection 34

5.7 Source and preparation of the *P. vivax* cryopreserved inoculum 35

5.7.1 Collection of *P. vivax* infected erythrocytes 35

5.7.2 Cryopreservation and storage of blood bank 36

5.7.3 Infectivity and characterisation of parasite stock 36

5.7.4 Testing of source patient and blood donors for blood-borne and mosquito-borne infections 37

5.7.5 Sterility and screening for blood-borne infections of cryopreserved blood bank 38

5.8 Source and preparation of the *P. falciparum* cryopreserved inoculum 38

5.8.1 Collection and cryopreservation of inoculum 38

5.8.2 Previous Studies 39

5.9 Conduct of CHMI trials 40

5.9.1 Clinical presentation post-CHMI 41

5.9.2 Ethical considerations of CHMI trials 42

5.9.3 Oxford's experience conducting CHMI trials 42
5.9.4 Conducting CHMI in the context of the COVID-19 pandemic 43
6 Description and justification of study design 45
6.1 Overview 45
6.2 Study groups 45
6.2.1 VAC069A Initial challenge and dose-finding: Groups 1 (n=2), 2 (n=2) and 3 (n=2). 46
6.2.2 VAC069B Secondary re-challenge with new primary controls: Groups 4 (n=6) and 6 (n=2-3). 47
6.2.3 VAC069C Tertiary (n=6) and secondary (n=2) re-challenges with new primary challenge controls (n=4-8): Groups 5, 7 and 9. 47
6.2.4 VAC069D Tertiary (n=2) and secondary (n=4-8) re-challenges with new primary challenge controls (n=4-8): Groups 8, 10 and 12. 48
6.2.5 VAC069E Re-challenge with *P. falciparum*: Group 13. 48
6.3 Duration of volunteer participation 50
6.3.1 Definition of the start and end of the trial 50
6.4 Potential risks for volunteers 50
6.4.1 Phlebotomy 50
6.4.2 Risk of Infection with Blood Borne Organisms 51
6.4.3 *Plasmodium vivax* and *falciparum* infection 53
6.4.4 Medications dispensed to volunteers in course of trial 53
6.4.5 Risk of reaction to the blood sample 54
6.4.6 Mosquito bites (for volunteers partaking in direct feeding assays only) 54
6.5 Potential benefits for volunteers 55
7 Recruitment and withdrawal for trial volunteers 56
7.1 Informed consent 56
7.1.1 Informed consent 56
7.2 Inclusion and exclusion criteria 57
7.2.1 Inclusion criteria 57
7.2.2 Exclusion criteria 58
7.2.3 Prevention of 'Over Volunteering' 59
7.2.4 Exclusion criteria on day of CHMI 59
7.3 Withdrawal of volunteers 60
7.4 Pregnancy 60
8 Controlled Blood-Stage Malaria Infection Inoculum 62

8.1 Preparation of the inoculum 62
8.2 Administration of the inoculum 62
9 Treatment of trial volunteers 63
9.1 Trial sites 63
9.2 Study procedures 63
9.2.1 Observations 63
9.2.2 Blood tests 63
9.2.3 Urinalysis 64
9.2.4 Electrocardiogram 65
9.2.5 SARS-COV-2 testing 65
9.3 Clinical reviews 65
9.3.1 Screening visits 65
9.3.2 Re-screening visits for participants not yet enrolled in the study 66
9.3.3 Re-screening visits prior to 2nd and 3rd Challenges 66
9.3.3 Two days before CHMI (C-2) 66
9.3.4 Day of CHMI (C0) 66
9.3.5 Days 1-28 post-CHMI (C+1 – C+28) 67
9.3.6 Direct feeding of mosquitos 69
9.4 Algorithm for initiation of treatment 69
9.4.1 Algorithm for initiation of treatment in Groups VAC069A&B 69
9.4.2 Algorithm for initiation of treatment in Groups VAC069C-E 72
9.4.3 Participants with fever post malaria challenge and COVID-19 testing 75
9.5 Malaria management 76
9.5.1 Malaria management – Riamet 76
9.5.1 Malaria management – Malarone 76
9.5.2 Malaria management – supportive medications 76
9.5.3 Follow-up after commencing malaria treatment 77
9.5.3 Criteria for inpatient transfer to the NHS 77
9.6 Safety measures for conduct of CHMI 77
9.6.1 Measures to be taken if a volunteer goes missing post-CHMI 78
9.7 Follow-up post-treatment 79
9.7.1 Six days after initiation of treatment (T+6) 79
9.7.2 Nine days after initiation of treatment (for VAC069C-D) (T+9 (+2)) 79

9.7.3 Ten days after initiation of treatment (for VAC069E) (T+10 (±2)) 79
9.7.5 Days 56 and 96 post CHMI (for VAC069B-E) (C+56 (±14) and C+96 (±14)) 79
9.8 Secondary and tertiary blood-stage CHMI 79
10 Assessment of scientific objectives 104
10.1 Primary endpoints 104
10.2 Secondary endpoints 104
11 Assessment of safety 105
11.1 Definitions 105
11.2 Causality assessment 106
11.3 Reporting procedures for all AEs (see SOP VC027) 107
11.3.1 Severity grading for laboratory AEs 108
11.3.2 Reporting procedures for serious AEs 108
11.3.4 Reporting procedures for SUSARs 108
11.3.5 Procedures to be followed in the event of abnormal findings 108
11.4 Foreseeable medical occurrences 108
11.5 Safety profile review of CHMI and antimalarial treatment 109
11.6 Local safety monitor 109
12 Statistics 110
12.1 Sample size 110
13 Quality control and quality assurance procedures 111
13.1 Investigator procedures 111
13.2 Monitoring 111
13.3 Modification to protocol 111
13.4 Protocol deviation 111
13.5 Audit & inspection 111
13.6 Serious breaches 111
13.7 Trial progress 112
13.8 Publication policy 112
13.9 Intellectual Property 112
14 Ethics 113
14.1 Declaration of Helsinki 113
14.2 ICH guidelines for good clinical practice 113
14.3 Approvals 113

14.5 Volunteer confidentiality 113
15 Data handling and record keeping 114
15.1 Data handling 114
15.2 Record keeping 114
15.3 Source data and electronic case report forms (eCRFs) 114
15.4 Data protection 114
16 Financing and insurance 115
16.1 Financing 115
16.2 Insurance 115
16.3 Compensation 115
17 Appendices 116
18 References 117

1 SYNOPSIS

Title	A clinical study to assess the safety and feasibility of controlled blood-stage *Plasmodium vivax* human malaria infection through experimental inoculation of cryopreserved infected erythrocytes in healthy malaria-naïve UK adults
Trial Centre	Centre for Clinical Vaccinology & Tropical Medicine, Jenner Institute, University of Oxford, Churchill Hospital, Old Road, Headington, Oxford, OX3 7LE
Trial Identifier	VAC069
Clinical Phase	N/A
Design	Controlled human malaria infection (CHMI) study
Population	Healthy adults aged 18 – 50 years
Sample Size	Total: 20 - 24 healthy malaria-naïve volunteers<br>Group 1: 2 volunteers, undiluted inoculum<br>Group 2: 2 volunteers, 1:5 diluted inoculum<br>Group 3: 2 volunteers, 1:20 diluted inoculum<br>Group 4: secondary challenge of all 6 volunteers from Groups 1-3 above<br>Group 5: tertiary challenge of all 6 volunteers from Group 4<br>Group 6: 2-3 volunteers primary challenge<br>Group 7: secondary challenge of all 2 volunteers from Group 6<br>Group 8: tertiary challenge of all 2 volunteers from Group 7<br>Group 9: primary challenge of 4-8 volunteers<br>Group 10: secondary challenge of all 4-8 volunteers from Group 9<br>Group 12: primary challenge of 4-8 volunteers<br>Group 13: repeat challenge with *P. falciparum* of volunteers from Groups 10 and 12
Follow-up duration	Groups 1-8: Approximately 2.5 years from the time of primary challenge<br>Groups 9-12: Approximately 18 months<br>Group 13: Approximately 3 months

	These estimates may vary in accordance with the specified time windows for each attendance.
Planned Trial Period	The start date of the trial will be in January 2019. The end date of the trial will be within approximately 4 years after primary CHMI of groups 1-3.
Primary Objectives	To assess the safety and feasibility of blood-stage controlled human *P. vivax* malaria infection in healthy human volunteers, through experimental injection of cryopreserved *P. vivax* infected erythrocytes at different doses<br>To choose the optimal inoculation dose to take forward to future *P. vivax* CHMI studies.
Secondary Objectives	To assess safety and feasibility of a secondary and tertiary homologous controlled blood-stage human *P. vivax* malaria infection<br>To assess the immune response to primary, secondary and tertiary homologous and heterologous *Plasmodium* infection, through experimental injection of *P. vivax* and *P. falciparum*-infected erythrocytes<br>To assess gametocytaemia following primary, secondary and tertiary *P. vivax* infection through experimental injection of *P. vivax* infected erythrocytes.<br>To assess transmission of gametocytes from infected volunteers to *Anopheles* mosquitoes.
Investigational products	None

2 ABBREVIATIONS

ACT	Artemisinin-based combination therapies
AE	Adverse event
AR	Adverse reaction
CBF	Clinical Bio-Manufacturing Facility
CCVTM	Centre for Clinical Vaccinology and Tropical Medicine
CHMI	Controlled human malaria infection
CI	Chief Investigator
CMV	Cytomegalovirus
CRF	Case Report Form or Clinical Research Facility
CYP2D6	Enzyme predicting Primaquine metabolism – Cytochrome P450 2D6
DARC	Duffy Antigen Receptor for Chemokines
DFA	Direct Feeding Assay
DSUR	Development Safety Update Report
EBV	Epstein-Barr Virus
ELISA	Enzyme linked immunosorbent assay
ELISPOT	Enzyme linked immunospot assay
EPI	Expanded Programme of Immunisation
FDA	United States Food and Drug Administration
GCP	Good Clinical Practice
GMP	Good Manufacturing Practice
HBsAg	Hepatitis B surface antigen
HCV	Hepatitis C virus
HIV	Human Immunodeficiency virus
HTLV	Human T cell Lymphotrophic virus
HLA	Human Leukocyte Antigen
ICH	International Conference on Harmonisation
IFN-γ	Interferon-gamma
IM	Intramuscular
IMP	Investigational Medicinal Product
LPS	Lipopolysaccharide
LSM	Local Safety Monitor
MFA	Membrane Feeding Assay
MHRA	Medicines and Healthcare products Regulatory Agency
MRC	Medical Research Council

µg	Microgram
NIHR	National Institute for Health Research
pIMD	potential Immune-Mediated Diseases
PCR	Polymerase Chain Reaction
PIS	Participant Information Sheet
QP	Qualified Person
RUNMC	Radboud University Nijmegen Medical Center
REC	Research Ethics Commitee
RGEA	Research Governance, Ethics and Assurance Team
SAE	Serious Adverse Event
SAR	Serious Adverse Reaction
SOP	Standard Operating Procedure
SUSAR	Suspected Unexpected Serious Adverse Reaction
TMF	Trial Master File
UKHSA	UK Health Security Agency
UOXF	University of Oxford
USMMVP	United States Military Malaria Vaccine Program
WHO	World Health Organisation
WTCRF	Wellcome Trust Clinical Research Facility

BACKGROUND & RATIONALE

3.1 Impact of *P. vivax* malaria and the need for a vaccine

Plasmodium vivax (*P. vivax*) is one of the five *Plasmodium* species to cause human malaria and accounts for the most cases of non-*P. falciparum* malaria worldwide. Geographically, it is the most widespread human malarial infection and approximately 2.5 billion people are at risk of contracting the infection in endemic areas [1]. It is estimated over one third of malaria cases outside of the African continent are due to *P. vivax* and that it accounts for 64% of malaria cases in the Americas, 30% in South-East Asia and 40% in the Eastern Mediterranean WHO regions. The burden is highest in Afghanistan, Ethiopia, India, Indonesia and Pakistan, which together account for 85% of *P. vivax* cases [2].

P. vivax has long been considered, and termed, 'benign' malaria, but more recently, large case series demonstrate that *P. vivax* infection is associated with significant morbidity and mortality. Complications reported include severe anaemia, respiratory and hepatic dysfunction, severe thrombocytopenia and coagulopathies, such as disseminated intravascular coagulation (DIC) [3]. *P. vivax* infection has also been associated with important sequelae for maternal and foetal health, correlating with increased rates of low birthweight, preterm delivery, and reduced foetal growth, as well as maternal and foetal deaths [4].

The socio-economic impact of this infection is not known, but in terms of overall global cost to the individual due to lost productivity this is estimated at US$ 1.4 to 4.0 billion per year [5]. Those affected by *P. vivax* malaria are typically poor with inadequate access to affordable healthcare and with little financial reserve, perpetuating the cycle of poverty [6].

Clinical cases are not only due to primary infection but relapses, which occur weeks to years after primary infection. Relapses are due to the ability of the *P. vivax* parasite to remain dormant in the liver in the hypnozoite stage, a lifecycle stage not seen with *P. falciparum* malaria [5]. Standard schizonticidal regimes are not effective against the hypnozoite stage. Radical cure of the hypnozoite stage requires therapy with the 8-aminoquinolone primaquine, currently the only licensed hypnozoitcidal anti-malarial, although tafenoquine, a new drug of the same class, given as a single dose, has recently been FDA-approved in the United States in patients aged 16 years and over. However, both 8-aminoquinolones carry a significant risk of severe haemolytic anaemia in individuals who are deficient in the glucose-6-phosphate dehydrogenase enzyme (G6PD), an inherited X-linked red blood cell enzyme disorder common in tropical and sub-tropical areas [7]. More recently, the importance of the cytochrome P450 enzyme, CYP2D6 in metabolism of primaquine to the active metabolite has been recognised [8, 9]. A common polymorphism in the *CYP2D6* gene for the cytochrome P450 enzyme results in poor conversion of primaquine to the active form, resulting in higher treatment failure rates. It is estimated that these two factors combined may make nearly 40% of the population at risk of *P. vivax* infection ineligible for primaquine therapy [7].

Control of *P. vivax* is also challenging, with re-emergence in areas where it has previously been eradicated. This is due to multiple factors including relapses, difficulty detecting asymptomatic infection, resistance to antimalarials and a lack of understanding of parasite biology [10]. Recent calls for control and 'eradication' of malaria worldwide [11] have focused attention on this neglected disease and the need for development of an effective *P. vivax* vaccine to be used alongside current control methods [12].

Consequently, the revised Malaria Vaccine Technology Roadmap to 2030 [12] now recognises the importance of *P. vivax* and calls for a vaccine to achieve 75% efficacy over two years – equally

weighted with *P. falciparum* in an era of renewed political will to move towards malaria elimination and eradication.

However, unlike *P. falciparum*, to date there has been relatively little research into this malaria species and one of the limiting factors has been the inability to culture *P. vivax* parasites *in vitro* over a prolonged period of time. More recently, research into vivax malaria has increased with candidate vaccines being developed and taken forward to clinical trial [13, 14]. However, none of these vaccines have progressed past a Phase IIa trial, and options for assessing the immune responses to new candidates are extremely limited.

3.2 Lifecycle of the *Plasmodium vivax* malaria parasite

The lifecycle of *P. vivax* is complex, involving both the vector (the *Anopheles* mosquito) and the human host (Figure 1). Sporozoites are injected into the human host when the female *Anopheles* mosquito takes a blood meal. From here they migrate within minutes to the liver, where they invade hepatocytes and the schizont develops (liver-stage). The mature schizont ruptures after around 7 days, releasing merozoites into the blood stream, which invade reticulocytes preferentially (unlike *P. falciparum* which invades all erythrocytes). As well as developing schizonts in the liver, *P. vivax* forms hypnozoites, a dormant stage which develop into schizonts weeks to years later, rupturing when mature and releasing merozoites to cause blood-stage malaria. It is this stage of the lifecycle that is responsible for relapse.

Once merozoites have invaded reticulocytes they are termed trophozoites, and again mature into schizonts which release merozoites after 2 days. These merozoites go on to infect other reticulocytes, and the blood-stage cycle continues. A subset of trophozoites develop into gametocytes, the sexual-stage of the malaria lifecycle, rather than forming schizonts. This occurs early in blood-stage infection with *P. vivax*, unlike *P. falciparum.* These are taken up by the mosquito in a blood meal. The sporogonic cycle takes place within the mosquito. The microgametocyte (male) exflagellates and enters the macrogametocyte (female) to form a zygote within the mosquito midgut. Zygotes become motile (ookinetes) and penetrate the midgut wall where they develop into oocysts. These rupture, release sporozoites which migrate to the mosquito salivary glands ready to be injected when the mosquito takes another blood meal [15].

The liver-stage of infection is asymptomatic. Symptoms, and subsequent complications, develop during the blood-stage.

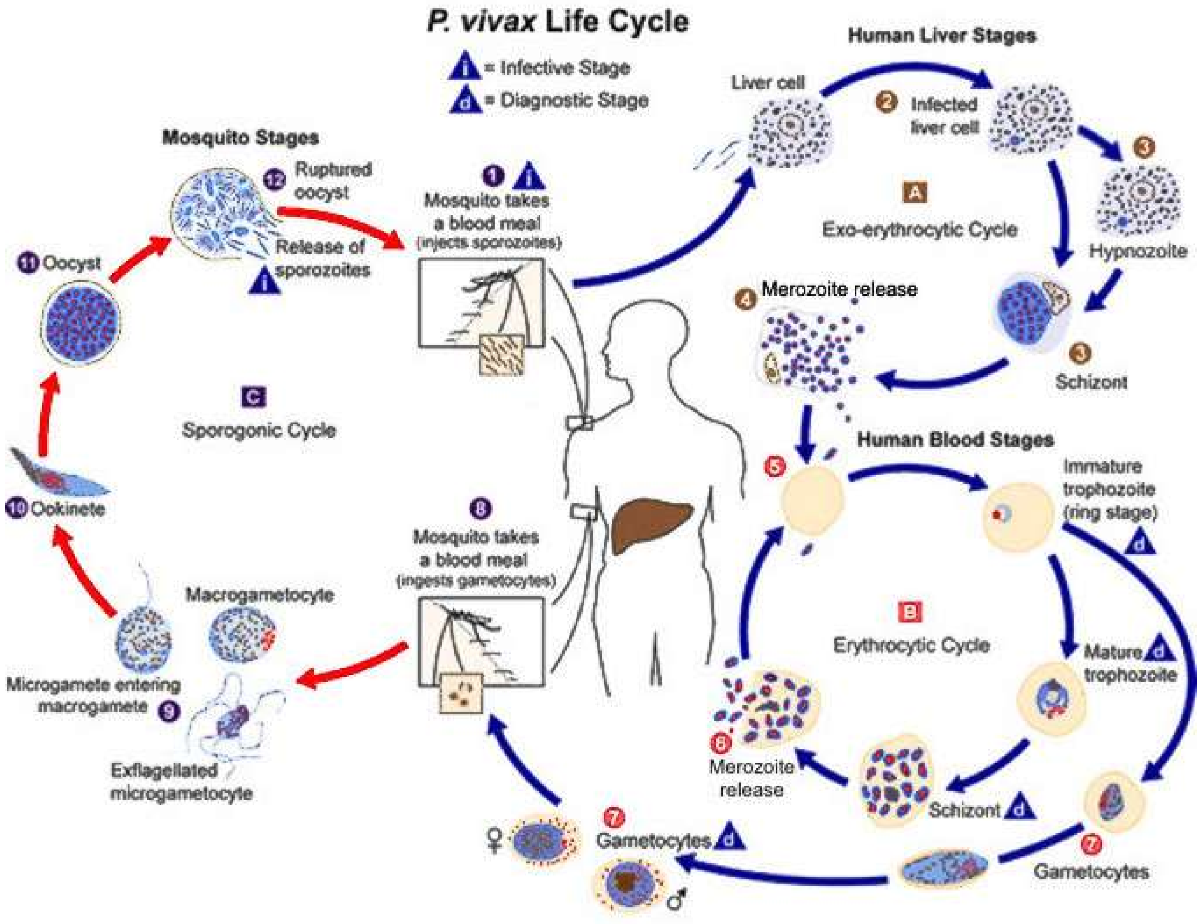

Figure 1: Lifecycle of *P. vivax* malaria

3.3. Challenges and directions in *P. vivax* vaccine development

The unique characteristics of *P. vivax* pose some specific challenges in studying its biology and designing strategies to control it. For example, the lack of a method for continuous culture of *P. vivax* blood-stages makes it difficult to perform *in vitro* growth inhibition assays to identify synergistic combinations of blood-stage antigens that can be targeted to inhibit *P. vivax* blood-stage growth with high efficiency. Several groups have recently succeeded in establishing short-term *P. vivax* culture for invasion assays using enriched reticulocytes from cord blood [16], but such methods are still dependent on access to fresh *P. vivax* isolates from malaria patients limiting the routine use of such assays to endemic regions. In the absence of a *P. vivax* blood-stage culture system, production of infected mosquitoes for sporozoite- or transmission-stage studies also requires access to *P. vivax* patients in endemic areas. In order to study hepatocyte invasion stages of *P. falciparum* as well as *P. vivax*, attempts have been made to establish *in vitro* liver models [17, 18]. In addition, a reliable humanised mouse model for *P. vivax* liver-stages is now available [19]. Both approaches will help to identify and test novel drugs and vaccines targeting hepatocyte stages. Studies on expression of parasite proteins in hypnozoites will determine if these latent stages can also be targeted with novel drugs and vaccines.

Most of the vaccines that are currently under development target individual stages of the parasite's lifecycle and are based on single antigens (Table 1 [13]). The development of an effective vaccine will likely require combination of multiple antigens that provide synergy to achieve high

efficacy. For example, achieving high rates of blood-stage growth inhibition may require targeting a combination of key blood-stage antigens involved in reticulocyte invasion. In addition, combination of blood-stage antigens with liver-stage antigens may be needed to achieve high efficacy, and inclusion of antigens from sexual- and mosquito-stages will be needed to inhibit transmission. It is therefore necessary to initiate efforts to combine antigens both within and across developmental stages to achieve synergy and attain the goal of developing a vaccine for *P. vivax* malaria with high efficacy.

3.4 Rationale for the development of a blood-stage challenge model

The development of a safe and reproducible *P. vivax* challenge model in malaria naïve humans could greatly accelerate clinical development of *P. vivax* vaccines, through selecting efficacious candidates for further clinical testing in more expensive and logistically challenging studies in the field. Herrera *et al.* have demonstrated the robustness and reproducibility of the *P. vivax* mosquito-bite delivered sporozoite challenge model in South America [20, 21], suitable for evaluation of efficacy of both pre-erythrocytic and blood-stage *P. vivax* vaccines. Within Europe, the safety and feasibility of a sporozoite *P. vivax* challenge, also delivered by mosquito bite, was demonstrated for the first time in April 2018 in Oxford within the ongoing VAC068 study (ClinicalTrials.gov Identifier: NCT03377296).

Mosquito-bite CHMI most closely models natural infection, and since cryopreserved *P. vivax* sporozoites are yet to be developed for human trials, remains the mainstay for CHMI in assessment of pre-erythrocytic vaccines. However, vaccines and anti-malarials targeting the erythrocytic stage, and with optimization, likely transmission-blocking candidates, may be evaluated by a blood-stage challenge model, which offers a number of advantages over sporozoite CHMI.

Infection of mosquitos requires a ready supply of blood containing gametocytes, from an infected patient. Without the possibility of long-term *P. vivax* ex-vivo culture, supply is limited to endemic areas. For CHMI studies conducted in non-endemic zones, this necessitates infection and importation of mosquitos from an appropriate area, with suitable entomological facilities and expertise, which may be logistically demanding, particularly in cases of vaccine efficacy studies, where timing of challenge may be critical.

Blood stage CHMI also permits both better characterization and standardization of the parasite inoculum. Parasite density of the cryopreserved isolates can be established, increasing homogeneity of initial parasite inoculum density, when compared to sporozoites delivered by mosquito bite, and allowing the possibility of controlled variations of the administered parasite load. Full genome sequencing, including screening for mutations known to be associated with drug resistance can also be performed prior to challenge. In addition, safety data can be accrued through multiple uses of the same inoculum. Together, standardisation of strain and density permit for better comparison between candidate vaccines, when evaluated through challenge using the same inoculum source.

Since parasitic load introduced via intravenous injection is smaller than that released following schizogony at the liver-stage, as in natural infection and sporozoite challenge, there is a longer period of blood-stage multiplication. This provides an extended period for assessment of parasite multiplication rate (PMR), before the development of clinical symptoms or diagnostic criteria might be reached. In addition, comparison of PMR between infectivity controls from blood-stage CHMI performed in Oxford within the VAC054 trial, assessing efficacy of the *P. falciparum* apical membrane antigen 1 (AMA1) vaccine candidate, FMP2.1/AS01, and historic controls challenged by mosquito-bite, illustrated that although mean PMR was comparable, blood-stage challenge

produced a much narrower range of PMR (smaller standard deviation with tight 95% confidence intervals). This provided greater power to detect partial vaccine efficacy, making blood-stage challenge a more robust tool for evaluation of candidate blood-stage vaccines [22]. This was indeed borne out in the recent Phase IIa efficacy study of the candidate vaccine RH5.1/AS01B, where the blood-stage challenge model was able to reliably detect a 17% reduction in the PMR between vaccinees and infectivity controls, with a high degree of confidence (P<0.02) (VAC063, ClinicalTrials.gov Identifier: NCT02927145).

Significantly, in addition to advantages seen with *P. falciparum* blood-stage CHMI, *P. vivax* blood-stage challenge provides a mode of infection which by-passes the liver stage. This eliminates the possibility of hypnozoite formation and therefore, risk of relapse. Equally, there is no requirement for CYP2D6 phenotype screening and primaquine therapy. A *P. vivax* blood-stage challenge is therefore less restrictive and confers important safety benefits over sporozoite challenge.

Vaccine candidate	Development Phase	Lifecycle stage	Antigen	Delivery system
VMP001	Phase I/IIa END	Liver-stage	PvCSP	Rec. protein-AS01B
CSV-S,S	Pre-clinical	Liver-stage	PvCSP	HBsAg fusion-AS01B
PvCSP-LSP	Phase I END	Liver-stage	PvCSP	Synthetic peptides-Montanide ISA 720
ChAd63-PvTRAP/MVA-PvTRAP	Pre-clinical	Liver-stage	PvTRAP	Prime-boost, viral vectors
PvDBPII	Phase I	Blood-stage	PvDBP	Rec. protein-GLA-SE
PvDBPII-DEKnull	Pre-clinical	Blood-stage	PvDBP	Rec. protein
ChAd63-PvDBPII/MVA-PvDBPII	Phase I/IIa	Blood-stage	PvDBP	Prime boost, viral vectors
$PvMSP1_{19}$	Pre-clinical	Blood-stage	PvMSP1	Rec. protein-Montanide ISA720
PvAMA1	Pre-clinical	Blood-stage	PvAMA1	Rec. protein-adjuvant
Pvs25H	Phase Ia END	Transmission-stage	Pvs25	Rec. protein-Alhydrogel; Rec. protein-Montanide ISA 51
Pvs28	Pre-clinical	Transmission-stage	Pvs28	Rec. protein-adjuvant
Pvs25-IMX313	Pre-clinical	Transmission-stage	Pvs25	Rec. protein-adjuvant
AnAPN1	Pre-clinical	Mosquito midgut Ag	AnAPN1	Rec. protein-adjuvant

Table 1: *P. vivax* vaccine candidates under development: taken from Ref [13].

3.5 Study rationale

As outlined above, the available methods for preventing and treating infections with *P. vivax* in the field are inadequate. Advancing the development of such methods needs renewed emphasis on understanding the biology, pathogenesis and transmission of *P. vivax*. A blood-stage challenge model would provide insights into this and greatly help validate and down select *P. vivax* vaccine candidates for further clinical testing in more time consuming and expensive field trials against natural challenge in endemic areas.

This study aims primarily to assess the safety and feasibility of controlled blood-stage human *P. vivax* malaria infection. This will be the first time that this source of *P. vivax* infected blood will be utilised and the first *P. vivax* blood-stage CHMI in Europe. If demonstrated to be safe and effective, it is intended that this parasitised blood bank be used in future trials to evaluate candidate vaccines.

As a secondary aim the safety of secondary and tertiary challenge will be assessed. Natural immunity to *P. vivax* is acquired over time, following repeated exposure. Homologous re-challenge would improve understanding of acquisition of the human immune response and parasite growth dynamics, with second and third exposures.

Only four controlled blood-stage *P. vivax* primary challenge studies have previously been conducted, of which only two have been published. These studies focused on safety and parasite growth dynamics and have not reported on the immunological response. No prior studies have evaluated repeated homologous blood-stage *P. vivax* CHMI. In this study, detailed immunological analyses, including gene expression studies of the parasite and host will be performed for primary, secondary and tertiary challenges. These approaches will provide new and more detailed insight into naturally acquired immunity than has been achieved before in any *P. vivax* blood-stage CHMI study, which could be crucial in guiding next-generation vaccine development.

3.6 Rationale for re-challenge with *P. falciparum*

The original hypothesis on the effects of homologous re-challenge was that using the same clone of *P. vivax* would lead to rapid anti-parasite immunity and lower parasite multiplication rate during re-challenge compared to the first malaria infection. However after completion of VAC069A to D with a significant number of participants completing secondary re-challenge, no anti-parasite immunity has been observed in any participants. However, of great interest, signs of clinical immunity have been consistently noted in participants undergoing secondary challenge. This clinical immunity is evidenced by reduced symptoms and signs of malaria disease with reduced fever and lower incidence of lymphopenia, thrombocytopenia and transaminitis recorded during secondary infection. This finding is in contrast to homologous re-challenge studies with *P. falciparum* where volunteers remained susceptible to clinical disease on repeat infection (Sandoval et *al.,* unpublished data) and is compatible with historical records that suggest *P. vivax* induces clinical immunity more quickly than *P. falciparum* [23-25].

The *P. vivax* CHMI model established in this study provides a unique opportunity to investigate the mechanisms of naturally acquired clinical immunity that minimise disease rather than pathogen load. Changing the malaria species in the final re-challenge from homologous to a heterologous challenge with *P. falciparum* will help determine whether *P. vivax* induces clinical immunity that is species-specific or species-transcending. The latter result would indicate that immunity is antigen independent and does not require adaptive immune control of inflammation. A mechanistic understanding of clinical immunity could aid the development of interventions that are designed to reduce the risk of disease in *P. vivax* infected patients. In addition, as *P. falciparum* and *P. vivax* are co-endemic in many areas, an understanding of the

effects of clinical immunity of *P. vivax* on *P. falciparum* would be of great importance in malaria control programmes.

This change in final *Plasmodium* species challenge remains in line with our experimental objectives (e.g. to assess the immune response to primary, secondary and tertiary infection) but instead allows us to switch focus from anti-parasite immunity to clinical immunity. This is entirely in line with the original goals of our funded programme of work to develop a rechallenge model of *P.vivax* malaria and examine mechanisms of naturally acquired immunity.

Given we have seen no evidence of anti-parasitic immunity after multiple *P.vivax* challenges and have acquired a clear understanding of the effect on clinical immunity (and now have the samples to explore immune mechanisms for this effect), there is no scientific gain from subjecting these participants to another *P. vivax* challenge, which makes the ethics of a re-challenge with *P. vivax* also questionable. By contrast, there is much to be gained scientifically from a final *P. falciparum* challenge for the reasons described, and this also fulfils the participants' expectations as they consented for and are expecting a secondary or tertiary malaria challenge.

Finally and importantly, there is no perceived clinical risk in subjecting these individuals to a heterologous challenge. While this has not been done before experimentally, exposure to multiple controlled homologous challenges has been shown to be safe by this group and others (Themistocleous et al, unpublished data, [26]), and exposure to multiple species occurs in the field, either simultaneously or sequentially, throughout an individual's lifetime. The net result appears to be accumulation of partial protection rather than association with any adverse outcome.

4 OBJECTIVES

Primary Objectives

To assess the safety and feasibility of controlled blood-stage *P. vivax* human malaria infection in healthy adult volunteers, through experimental injection of cryopreserved *P. vivax* infected erythrocytes

To choose, based on the first phase of the study (VAC069A), the optimal inoculation dose of *P. vivax* for use in blood-stage CHMI studies.

Secondary Objectives

To assess safety and feasibility of secondary and tertiary homologous blood-stage controlled human *P. vivax* malaria infection.

To assess the immune response to primary, secondary and tertiary homologous and heterologous *Plasmodium* infection, through experimental injection of *P. vivax and P. falciparum*-infected erythrocytes.

To assess gametocytaemia following primary, secondary and tertiary *P. vivax* infection through experimental injection of *P. vivax* infected erythrocytes.

To assess transmission of *P. vivax* gametocytes from infected volunteers to mosquito vectors

5 CONTROLLED HUMAN MALARIA INFECTION STUDIES

5.1 Microbial challenge studies of human volunteers

The deliberate infection of human volunteers with micro-organisms has contributed uniquely to our understanding of the pathogenesis, immune responses and the treatment and prevention of numerous microbial diseases including *P. falciparum* malaria, influenza, cholera, typhoid and hepatitis [27]. A review by the UK Academy of Medical Sciences on microbial challenge studies recognised that such studies are desirable for providing proof of concept for prophylactic and therapeutic interventions and can significantly accelerate progress to Phase III studies [27, 28].

5.2 Controlled human malaria infection with *Plasmodium falciparum*

Plasmodium falciparum is a microbe particularly well-suited to "challenge" studies. It has a relatively short asymptomatic period, a well-established diagnostic laboratory test (thick film microscopy), and rapid recovery with no long-term infectious state following appropriate and timely treatment. Studies involving controlled human malaria infection (CHMI) are a powerful tool for investigating malaria vaccine and prophylactic drug efficacy [29]. With an increasing number of candidate malaria vaccines being developed, the number of centres conducting CHMI studies is expanding in developed countries and now Africa [29].

Following the development of protocols for the continuous culture of *P. falciparum* in 1976 [30] and the generation of mature *P. falciparum* gametocytes *in vitro* in 1981 [31], it became possible to produce laboratory-reared infectious mosquitoes, meaning that CHMI trials could be performed more routinely [32].

The first well-documented CHMI study with laboratory-reared infectious mosquitoes was carried out in 1986 at the US Walter Reed Army Institute of Research (WRAIR), the US Naval Medical Research Institute (NMRI) and the US National Institutes of Health (NIH) [33]. The following year, the efficacies of the first recombinant protein and synthetic peptide *P. falciparum* vaccines were reported for experimentally infected volunteers [34, 35].

CHMI has now become established as a key tool to assess the efficacy of novel malaria vaccines and drugs; a total of 1,343 volunteers were experimentally infected with *P. falciparum* between 1985 and 2009 [29, 36]. As CHMI trials are carried out in a controlled environment, they allow detailed evaluation of parasite growth and immunological responses, providing key information for vaccine and drug development [29].

5.3 Controlled human malaria infection with *Plasmodium vivax*

In contrast to *P. falciparum*, there is much less experience with *P. vivax* in the modern CHMI era, with only a small handful of studies reported in the last few years. A key bottleneck has been the inability to culture *P. vivax* long-term *in vitro*. Sporozoite challenge with *P. vivax* also carries the risk of relapse weeks to years after infection if hypnozoites are not cleared from the liver (there is no dormant liver form in the *P. falciparum* lifecycle). In only two of the studies published to date has efficacy of immunisation been assessed (Table 2 – taken from Payne *et al*., Trends in Parasitology, 2017 [37]).

However, despite lack of modern CHMI, there is an extensive history of deliberate infection with *P. vivax*; most notably in malariotherapy, which was carried out for the treatment of neurosyphilis almost a century ago. The Austrian psychiatrist Julius Wagner-Jauregg later received a Nobel Prize for his work with this treatment [38] and the practice was widely adopted as the only effective treatment available at the time. Malariotherapy provided a wealth of information about *P. vivax* infection, which has been reviewed previously [39]. Deliberate

infection with *P. vivax* was also conducted in the USA from the 1940s to 1970s in prisoners involved in the Malaria Research Project at the Illinois State Penitentiary. The studies mainly examined compounds for their potential use as antimalarials [40]. Similar studies were also carried out at the United States Penitentiary, Atlanta. Key discoveries of the biology of *P. vivax* were made during this period including, for example, the association between Duffy negativity and resistance to *P. vivax* infection [41].

Following on from the studies of *P. vivax* infection conducted in Illinois, CHMI experiments were carried out to see if prior exposure to irradiated mosquitoes could confer protection by immunization. Rieckmann *et al.* [42] reported no protection against CHMI in three participants previously exposed to *P. vivax*-infected irradiated mosquitoes on four occasions at intervals of 2 – 4 weeks (total of <200 mosquitoes). During the 1970s, in Maryland, USA, three further studies assessed the ability to immunise following exposure to infected irradiated mosquitos, followed by challenge with non-radiated infected *Anopheles*. In these studies, there was no cross-species protection to the *P. vivax* Chesson strain, after exposure to *P. falciparum*, but exposure to X-irradiated Chesson and El Salvador *P. vivax* strains did confer protection against *P. vivax* CHMI following some of the 'immunisation phases', but not all and was achieved for a short duration (3-5 months), following exposure to hundreds of X-irradiated mosquitoes [22–24]. Recent data emerging from the field of *P. falciparum* sporozoite immunization indicate that the magnitude of protection, longevity of protection, and the ability to protect against heterologous strains requires increasing doses of sporozoites. These experiments in Maryland demonstrated that CHMI with both *P. falciparum* and *P. vivax* could be successfully carried out, but the studies were very small.

5.4 *P. vivax* sporozoite challenge in the modern era

More recent mosquito-bite delivered sporozoite CHMI trials have taken place in Cali, Colombia and at the Walter Reed Army Institute of Research (WRAIR), Maryland, USA. A total of four sporozoite challenge trials have been conducted in Cali. The first trial involved eighteen healthy volunteers, exposed to the bites of 2-10 *P. vivax* infected *Anopheles albimanus* mosquitoes, of which seventeen developed infection. Authors speculated that the volunteer who did not develop malaria had surreptitiously taken anti-malarial medication but this was never confirmed [20]. There were no serious adverse events (SAEs) in this trial, but seven volunteers required fluid therapy due to nausea and vomiting, and five developed blurred vision lasting 2-3 days after treatment initiation with chloroquine. The second *P. vivax* CHMI trial was carried out by the same group in Colombia, aiming to demonstrate the reproducibility of this method of infection using three different *A. albimanus* mosquito lots fed on blood from three *P. vivax*-infected donors [21]. Seventeen individuals whose red blood cells were positive for the Duffy antigen/chemokine receptor (DARC, "Duffy positive") and five Duffy negative controls were enrolled then randomly assigned to three groups (with six Duffy positive individuals in two of the groups and five in the third group). Following 2-4 bites from infected mosquitoes, all Duffy positive participants (and none of the Duffy negative participants) developed blood-stage malaria. A third *P. vivax* CHMI trial was carried out in Cali among both 'semi-immune' (previously-exposed; n=9) and malaria-naïve adult volunteers (n=7) [43]. Symptoms were significantly worse among the malaria-naïve subjects but there were no SAEs.

The most recent CHMI trial from the Cali group assessed "immunisation" through repeated exposure to radiation-attenuated sporozoites, delivered by mosquito bite, followed by challenge. Both Duffy positive and Duffy negative volunteers were recruited. Twelve Duffy positive participants and five Duffy negative controls completed the seven "immunisations" followed by CHMI, alongside two Duffy positive negative controls, exposed to non-irradiated, non-infected

mosquitoes. Two weeks after the final immunisation participants were treated with chloroquine and primaquine to clear any malaria infections that may have developed during the immunisation phase. CHMI was carried out at 8 weeks after final "immunisation", with each individual receiving 2 – 4 *P. vivax*-infected mosquito bites. There were no reported SAEs related to immunisation, although one volunteer developed severe elevation of hepatic transaminases (>10 times the upper limit of normal [x ULN]) with associated abdominal pain and vomiting following CHMI, with no alternative cause found. These symptoms resolved spontaneously. The protective efficacy of the immunisation schedule was 42% (five out of twelve Duffy positive participants protected). Interestingly, all of the volunteers protected in this trial were female [44].

Only one CHMI trial assessing a *P. vivax* vaccine has been published to date. The VMP001/AS01B vaccine was tested in healthy malaria-naïve adults at the WRAIR in the USA [45]. VMP001 is a soluble recombinant protein vaccine [46], encoding the *P. vivax* circumsporozoite protein (PvCSP) administered with the AS01B adjuvant (GlaxoSmithKline). The vaccine was administered to 30 volunteers in three cohorts (10 in each) at doses of 15 µg, 30 µg and 60 µg, given three times at a 4 week interval between the first and second dose; the third dose was given 8 (15 µg cohort), 6 (30 µg cohort) or 4 (60 µg cohort) weeks after the second. Twenty-nine volunteers completed the vaccination phase, with twenty-seven proceeding to sporozoite CHMI, delivered by mosquito bite, two weeks after final vaccination, alongside 6 malaria-naïve controls. Vaccine protective efficacy was 0%; all volunteers had developed thick blood film-detectable parasitaemia by day 13. The median pre-patent period for all immunised participants was 11.9 days versus 10.7 days for infectivity controls. The challenge was well tolerated with no untoward reactogenicity following mosquito bites and no untoward SAEs in challenge and treatment phases. Participants were treated with standard chloroquine and primaquine therapy with rapid clearance of infection. However, two volunteers went on to have multiple relapses. One participant experienced two relapses (at weeks 8 and 18 after CHMI), while the other experienced three (at weeks 11, 20 and 48 after CHMI) [9]. By study completion the participants had been followed up for 5 years, and had not had any further relapses [45]. Exploratory genotyping for the cytochrome P450 (CYP) allele CYP2D6 was undertaken in 25 of the 33 volunteers. The volunteers with relapses were found to have either an intermediate-metaboliser phenotype or poor-metaboliser phenotype. These phenotypes were associated with significantly lower levels of primaquine clearance 24 hours after dosing [9].

The first *P. vivax* sporozoite challenge in Europe was recently conducted in April 2018, in Oxford within the VAC068 study (ClinicalTrials.gov Identifier: NCT03377296). Two malaria-naïve adult volunteers were exposed to five infectious bites (bites by mosquitos with >10 sporozoites as detected on microscopic examination of salivary glands post-feeding) by *Anopheles dirus* mosquitoes. Infection in the mosquitoes was established by direct membrane feeding on the blood from a *P. vivax* infected source patient, in Songkhala, Southern Thailand, before transport of the infected mosquitoes to the UK. Both volunteers were successfully infected, with parasitic DNA being detectable from day 9 after challenge by qPCR in both subjects. Symptoms consistent with malaria infection, including fatigue and subjective feverishness, chills, headache, loss of appetite, nausea and malaise, were reported by one volunteer from day 11 after challenge and on day 14 by the second volunteer. Fever was detected in both subjects on day 14. Thick film microscopy was positive (≥2 morphologically normal asexual parasites seen in 200 high-power (1000x) fields) in the asymptomatic volunteer on the evening of day 13 and at day 14 in the more symptomatic volunteer, leading to diagnosis on day 14 in both volunteers. A 250mL blood donation was made by each volunteer, immediately prior to initiation of anti-malarial treatment, without complication, for cryopreservation and future use as a parasitised blood inoculum for

blood-stage challenge. After 48 hours' anti-malarial treatment, thick film microscopy was negative and parasitaemia by qPCR had substantially reduced on two consecutive samples. Both volunteers completed the clinical follow-up period, including a two-week course of primaquine for radical cure of hypnozoites, until 90 days post-challenge, without safety concerns. To-date (more than 8 months post completion of primaquine therapy) there has been no report of symptoms to suggest relapse in either volunteer (email questionnaire follow-up is being continued for 5 years).

Trial Site	Number of volunteers	Pre-patent period (days)[a]	Number of infected mosquitoes OR inoculum	Number of volunteers with patent parasitaemia	References
Sporozoite (mosquito-bite) CHMI studies					
Cali, Columbia	18	9 – 13	2 - 10	17/18	[20]
Cali, Columbia	17 Duffy positive<br>5 Duffy negative	9 – 16	2 - 4	17/17 (Duffy positive)<br>0/5 (Duffy negative)	[21]
Cali, Columbia	7 malaria-naïve<br>9 semi-immune	11 – 13	2 - 4	16/16	[43]
WRAIR, USA	27 vaccinees<br>6 infectivity controls	10 – 13<br>10 – 11	5		[45]
Cali, Columbia	12 Duffy positive vaccinees<br>2 Duffy positive controls<br>5 Duffy negative controls	12 – 13	2 - 4	7/12 vaccinees<br>2/2 Duffy positive controls<br>0/5 Duffy negative controls	[44]
Blood-stage CHMI studies					
QIMRB, Australia	2	8-9	13,000 genome equivalents	2/2	[47]
QIMRB,<br>Australia	6	8-9	31,786 (± 11,947) genome equivalents (= 15 ± 5 viable *P. vivax* parasites)	6/6	[48]

Table 2: Overview of Published *Plasmodium vivax* CHMI Studies. [a]The pre-patent period refers to the period before malaria diagnosis: by blood film (sporozoite) or qPCR (blood-stage).

5.5 Modern blood-stage controlled human *P. vivax* infection

There have been four studies of blood-stage *P. vivax* CHMI, of which two are published, all conducted at the Queensland Institute of Medical Research (QIMR), Brisbane Australia. Both published studies used a cryopreserved source of parasitised erythrocytes obtained from a returned traveller with blood group A, Rhesus negative, who was infected with *P. vivax* during a trip to the Solomon Islands [47, 48].

In the first study, two volunteers were inoculated with 270µL of reconstituted parasitised red cells, containing approximately 13,000 genome equivalents. Both subjects were successfully infected, with PCR becoming positive on day 8 or 9. Clinical symptoms consistent with malaria infection were present from day 11 in one volunteer, who reported myalgia, headache and malaise and day 14 in the other, with both experiencing chills and sweats at day 14. Treatment with artemether/lumefantrine was started at day 14 and volunteers were admitted for ongoing monitoring. Both volunteers experienced worsening of symptoms over the initial 12 hours post-treatment, including nausea and vomiting, headache, sweats and rigors in one volunteer. Pyrexia above 39°C was detected in both volunteers. After 24 hours of anti-malarials, all symptoms had resolved, although non-tender clinically palpable mild splenomegaly was detected in one subject at day 15. By day 28, splenomegaly had resolved fully and no other adverse events were reported by either subject in the period since discharge. Whole genome sequencing was performed, demonstrating a genetic sequence most closely related to Cambodia strains, of the publically available strains. Gametocytaemia was measured by quantitative reverse-transcriptase PCR for the *pvs25* transcript, a mature gametocyte marker. *Pvs25* transcripts were detectable from day 11 or 12 and peaked at day 14 in both volunteers, coinciding with peak quantitative parasite PCR, prior to the initiation of anti-malarial treatment [47].

A second study was conducted to demonstrate reproducibility of the blood-stage controlled infection method and assess transmissibility of infection to *Anopheles stephensi* mosquitoes. Six volunteers were infected after receiving an inoculum containing approximately 31,786 (+/-11947) genome equivalents, estimated by investigators using linear regression growth modelling to equate to 15+/-5 viable parasites. Evolution of clinical symptoms was similar to the first study, with volunteers developing symptoms in keeping with malaria infection on days 11-13 (mean 12.2). There were no serious adverse events during the course of either study, but for 4 of 6 volunteers, liver enzymes became significantly elevated, above 5 times the upper limit of normal. Mild bilirubinaemia was also detected in three subjects, however increases were transient and did not exceed 2 times the upper limit of normal. No clinical manifestations were associated with biochemical abnormalities. No other cause for this deranged liver function was found and liver ultrasound was normal but abnormalities subsequently resolved spontaneously without sequelae [48]. Transmissibility of infection was assessed by both direct feeding assay (DFA) and membrane feeding assay (MFA), carried out 3 days prior to the anticipated peak parasitaemia and initiation of treatment on day 14, as predicted from the first study. A total of 16 DFAs and 32 MFAs were performed, with rates of successful feeding above 90% across the two methods, without significant difference between the two. Overall, 1801 mosquitoes were dissected for examination of oocysts 7-9 days after feeding, of which 1.8% (n=32) were demonstrated to be infected, although the rate of infectivity was five times higher for DFA when compared to MFA. Other atypical ovoid structures were also detected by investigators on the mosquito midgut, which could not be identified by PCR as either microsporidia or fungal pathogens, known to infect mosquitoes. For standardisation of the method, if used for drug or vaccine efficacy evaluation, other methods for confirmation of oocysts will be required. The reason for low infectivity could not be determined, however, a possible incompatibility of the Solomon Island

derived parasite strain and the laboratory-reared *A. stephensi* colony, which had originally been sourced from India, was cited by the authors as a potential contributory factor. While small, these studies demonstrate the safety, feasibility and reproducibility of a *P. vivax* blood-stage challenge model and show the potential for use as a transmission model for evaluation of transmission-blocking vaccine candidates, although the methodology is yet to be optimised.

5.6 Re-challenge in controlled human malaria infection

Re-challenge is well established in the sporozoite controlled infection model, with both mosquito bite delivery or more recently, inoculation of cryopreserved sporozoites. This has primarily been in assessment of durability of pre-erythrocytic vaccine-induced responses, where sterile protection has been established at primary challenge. However, repeated sporozoite challenge has also been investigated as a form of "immunisation". There is much less experience with repeated controlled blood-stage malaria infection for both *P. falciparum* and *P. vivax*, although, historically, re-challenge was routinely performed as part of deliberate malaria infection in malariotherapy for neurosyphilis patients. In repeated infection, malariotherapy patients had a reduced number of fever episodes and a log reduction in the maximum mean parasite count after a homologous *P. vivax* re-challenge, suggesting conferred protection from a prior challenge. However, with little experience in human blood-stage re-challenge in the modern era, the level of immunity afforded remains largely unknown.

There have been no previous studies assessing repeated *P. vivax* blood-stage CHMI, and only two studies which have assessed *P. falciparum* blood-stage re-challenge. A group at the Queensland Institute of Medical Research, Australia, assessed repeated exposure to small doses of parasitised erythrocytes, followed by administration of anti-malarial treatment at sub-clinical infection, in five malaria-naïve healthy volunteers. After three rounds of low-dose challenge and cure, three out of the four volunteers who completed the study did not develop infection. However, with very small numbers, lack of a control group and detection of residual atovaquone, which may have caused this protective effect, the interpretation of these results is difficult.

The first *P. falciparum* blood-stage re-challenge in volunteers who had reached criteria for malaria diagnosis, before initiation of treatment, was conducted in a recent study at Oxford (VAC063, ClinicalTrials.gov Identifier: NCT02927145). Nine volunteers vaccinated with the RH5.1/AS01 candidate, and eight unvaccinated controls received a second blood-stage challenge, alongside six malaria naïve controls receiving a primary challenge. Vaccinees were re-boosted two weeks before secondary challenge (at a timepoint four months after primary challenge). After secondary challenge, higher vaccine-induced antibody responses and a greater significant reduction of parasite multiplication rate was demonstrated in vaccinated volunteers compared to the first CHMI, indicating an enhanced partially protective effect upon second challenge. However, remarkably a significant reduction in parasite multiplication rates compared to primary CHMI was also seen in two of eight re-challenged control volunteers. This was particularly striking in one participant who had no detectable infection by qPCR until day 18, a significant delay as compared to initial PCR patency at day 3-5 in other volunteers. Findings lend support to the development of protective immunity, following a prior blood-stage exposure, underlying the need for adequate controls in assessing vaccine efficacy in re-challenge studies. Furthermore, these preliminary results also suggest potential for re-challenge studies to contribute to understanding of acquisition of blood-stage immunity.

5.6.1 Safety of re-challenge in controlled human malaria infection

Safety of re-challenge is indicated by the natural history of induced protection from previous malaria exposure in endemic areas, as well as the wealth of data from repeated infection

administered as malariotherapy, which strongly indicates that exposing a previously malaria-exposed volunteer to a second or third challenge would not confer any additional risk over that of a first challenge. Indeed, it is highly possible that this may in fact confer some protective immunity, as in the natural exposure setting, where asymptomatic infections and tolerance of high levels of parasitaemia without symptoms or clinical signs of malaria are seen. Repeated challenge may therefore result in a milder infection and/or delayed time to diagnosis. Alternatively, if there is no immunity from the previous challenge or challenges, then the expected course of a second or third challenge would be comparable to the primary CHMI, with no change in risk.

Hundreds of healthy volunteers have previously been re-challenged with *P. falciparum* in sporozoite challenge, delivered either by mosquito bite, or infected sporozoite, in the UK, Netherlands and the USA. No safety concerns were reported in any of these trials [49-52] In the only previous study assessing a second homologous *P. falciparum* blood-stage challenge in volunteers reaching criteria for malaria diagnosis, recently conducted in Oxford (VAC063, ClinicalTrials.gov Identifier: NCT02927145), preliminary data is also supportive of a level of risk of re-challenge, which is the same as primary controlled malaria infection. During the second challenge in this study, solicited haematological and biochemical severe adverse events occurred at the same frequency as among controls undergoing a primary challenge and there were no serious adverse events (SAEs). All solicited and laboratory adverse events were self-limiting. Unsolicited AEs have yet to be peer reviewed, however, there were no events of medical concern in either vaccinated or control volunteers receiving a second challenge.

5.7 Source and preparation of the *P. vivax* cryopreserved inoculum

The cryopreserved *P. vivax* parasitized red blood cells that will be used were produced by Dr Angela Minassian, Prof Simon Draper and colleagues at the University of Oxford in April 2018, as part of the VAC068 study (ClinicalTrials.gov Identifier: NCT03377296), approved by the South Central - Oxford A Research Ethics Committee.

5.7.1 Collection of *P. vivax* infected erythrocytes

Parasitised erythrocytes were collected from two *P. vivax*-infected volunteers (otherwise healthy), who donated blood following sporozoite *P. vivax* CHMI, delivered by mosquito bite. Volunteers were infected via five infectious bites (defined as bites by mosquitoes with >10 sporozoites as detected on microscopic examination of salivary glands post-feeding) from infected laboratory-reared *Anopheles dirus* mosquitoes, under controlled conditions. Infected mosquitoes were provided by, and imported from, The Mahidol University, Bangkok. The colony is maintained through feeding on rigorously screened human blood (provided by the Red Cross) to induce egg-stimulation, animal blood is never used. For infection of mosquitoes, a source patient was recruited from a medical clinic in one of Thailand's Southern endemic areas – Songkhala (~7 hours' drive from the Vivax Research Unit in Bangkok). Microscopic diagnosis of the source patient was established at both the field site and in the Bangkok reference laboratory, where PCR analysis confirmed *P. vivax* mono-infection. Molecular speciation by PCR (research-grade laboratory assay) was verified at the Jenner Institute Laboratories and clonality of the infection was established by the Wellcome Trust Sanger Institute in Cambridge, UK, on a 2mL whole blood sample, sent directly by courier from Thailand to Oxford. Mosquitoes were infected via direct membrane feeding with blood from the source patient, and infectivity was confirmed at 5-7 days post-feeding through oocyst count on dissection of the midgut.

Both UK volunteers ("Donors") were followed from days 1-6 post-challenge by telephone and from the evening of day 6 at twice-daily clinic visits. Blood sampling was performed at each visit

for thick film microscopy and PCR. Clinical symptoms consistent with malarial infection were reported from day 11.5 post-challenge in Donor 1 and from day 14 in Donor 2, with both becoming febrile at day 14. Thick film positivity was defined as the detection of at least two morphologically normal malaria parasites seen in 200 high-power (1000x) fields. Thick film microscopy was positive at day 14.5 in volunteer 1 and at day 13.5 in volunteer 2, corresponding to 31,010 and 16,717 genome copies/mL by PCR, respectively. Blood donation was performed in each volunteer immediately prior to initiation of treatment on the morning or afternoon of day 14. A 250mL blood sample was collected using aseptic technique, via a whole blood donation kit (Leuokotrap WB, Haemonetics Corp), containing an in-line leukodepletion filter. For anonymization of the blood donor, blood collected for cryopreservation was labelled with either "Donor 1" or "Donor 2". Traceability of the blood donor is however maintained within a confidential clinical record, which may be accessed by the Chief Investigator, or by key members of the clinical team, to whom responsibility is delegated, if requested by the sponsor or other external bodies. After blood collection, treatment with artemether/lumefantrine (Riamet®) was administered, followed by a two week course of primaquine. No supportive treatment or admission was required. Follow-up (clinic visits) until 90 days post-challenge was completed, with no safety concerns. Ongoing follow-up by email will continue for 5 years in order to monitor for relapse.

5.7.2 Cryopreservation and storage of blood bank

After collection, blood was maintained at 37°C and transported immediately to the Jenner Institute Laboratory, University of Oxford. All laboratory processing was conducted under GMP-like conditions, under QP and QA oversight. Procedures were performed within fumigated microbiological safety cabinets, under precautions in compliance with Containment Level 3 Code of Practice and with sterile technique.

First, the red cells were separated from plasma by centrifugation of the leukodepleted blood before mixing with Glycerolyte 57 (at 1:2 erythrocyte to Glycerolyte 57 volume ratio). The first 20% of Glycerolyte 57 was added dropwise with gentle agitation, the suspension was then incubated for 5 minutes at room temperature before the remaining Glycerolyte 57 was added. The RBC-Glycerolyte mixture was aliquoted into 1.5mL cryovials and frozen at -80°C for one night before transfer to a dedicated liquid nitrogen tank. After 6 days, samples were transferred to Thermo Fisher Bishop's Stortford temperature-monitored liquid nitrogen facility, Hertfordshire, UK, where samples are stored on behalf of the University of Oxford. This process was performed under conditions similar to those used for blood thawing in previous CHMI trials at Oxford and according to the Jenner Institute Laboratory SOPs.

5.7.3 Infectivity and characterisation of parasite stock

Confirmation of parasitic density within the blood collected for cryopreservation was performed via microscopy and quantitative PCR on the leukodepleted blood samples. Thick film microscopy demonstrated 11 asexual parasites per 1µL leukodepleted blood for Donor 1, and 5 asexual parasites per 1µL leukodepleted blood for Donor 2. Quantitative PCR confirmed the presence of 23,566 genome copies/mL in Donor 1, and 14,078 genome copies/mL in Donor 2. This suggested minimal parasitic loss through the leukodepletion process when compared to the diagnostic PCR values of 31,010 and 16,717 genome copies/mL, respectively.

Viability has also been demonstrated in an *ex-vivo* short-term culture of thawed infected red blood cells in enriched McCoy 5A medium. Parasitic growth was detectable by light microscopy, quantitative PCR and flow cytometry through an initial 40-hour growth cycle in samples collected

from Donor 1, with normal progression of normal morphology as seen on Giemsa stained thick and thin films. However, parasite growth was sub-microscopic in samples obtained from Donor 2. Therefore, we intend to proceed with further testing of cryopreserved samples from Donor 1, and will use this as the challenge inoculum in this and future studies. Further testing will include whole genome sequencing to further characterize the parasitic strain, with particular scrutiny for SNVs present in the five putative drug resistant genes *pvdhfr*, *pvdhps*, *pvcrt*, *pvmdr* and *pvmrp*.

5.7.4 Testing of source patient and blood donors for blood-borne and mosquito-borne infections

Initial testing for blood-borne infections and mosquito-borne disease was carried out on blood samples from the original source patient in Thailand, taken at the time of *P. vivax* diagnosis. Since *Anopheles* is a known vector of *Wucherichia bancrofti*, the main causative agent of lymphatic filariasis, blood was screened for filarial disease via rapid diagnostic test for IgG_4 antibodies to the *W. bancrofti* Wb123 antigen (SD BIOLINE Lymphatic Filariasis IgG_4) at the Thai field site. In addition, although there is no evidence that blood-borne infections can be transmitted by mosquito bite, for maximal assurance of safety, serology for HIV-1 and HIV-2, HTLV, Hepatitis B and C and syphilis were performed on a serum sample from the Thai source patient, at Oxford University Hospitals NHS Trust. Due to anecdotal reports of Japanese B encephalitis and Chikungunya in *Anopheles* species, blood from the source patient was also screened for both arboviral infections by PCR, as performed by the WRAIR unit, who used the same mosquito source for a CHMI trial that assessed the efficacy of the VMP001/AS01 *P. vivax* circumsporozoite recombinant protein vaccine[45]. As a further precautionary measure, although *Anopheles* species are not known to be vectors of Dengue, Zika or West Nile virus, on the independent advice of clinical Tropical Medicine specialist, Prof. Nicholas Day, Professor of Tropical Medicine and Director of the Mahidol Oxford Tropical Medicine Research Unit in Thailand, PCR for these infections was performed on the source patient's blood. In summary, molecular analysis (PCR) for Chikungunya, Japanese B encephalitis, West Nile, Dengue and Zika viruses was performed on whole blood at the Rare Imported Pathogens Laboratory (RIPL) in the UK. All source patient infection screen tests were negative.

Both UK volunteers ("donors") were subjected to screening for blood-borne infections, in line with the Joint UK Blood Transfusion and Tissue Transplantation Services Professional Advisory Committee guidelines https://www.transfusionguidelines.org/red-book/chapter-9-microbiology-tests-for-donors-and-donations-general-specifications-for-laboratory-test-procedures/9-1-general-requirements. Testing comprised serological tests for HIV-1 and HIV-2, hepatitis B and C, syphilis (anti-treponemal Ab) and HTLV-1 and HTLV-2 at screening, and nucleic acid amplification tests for HIV-1 and HIV-2 and hepatitis B and C, as well as repeat serological tests for HTLV-1 and HTLV-2 and syphilis 7 days before sporozoite challenge. Neither volunteer had travelled to any area which represented a risk of exposure to any other form of blood-borne infection. All tests were negative. Volunteers also underwent repeat serological testing for HIV, hepatitis B and C, syphilis, HITLV-1 and HTLV-2, 90 days after challenge was undertaken, to ensure that no seroconversion from a recently-acquired infection (that may have been undetectable around the time of challenge) had occurred since the challenge period. All tests remained negative.

In addition, volunteers were screened for Epstein-Barr virus (EBV) and Cytomegalovirus (CMV) prior to enrolment. Both volunteers were IgG seropositive for both infections. However, leukodepletion has been shown to be effective in preventing transmission of both EBV and CMV [53] therefore, EBV-CMV concordant serostatus would not be considered a requirement for recipients of this donated blood in this study, unless PCR testing of the blood-bank is positive for either infection. This is in line with conduct of previous *P. falciparum* CHMI studies at Oxford,

which have utilised a cryopreserved source of infected erythrocytes from a donor who was seropositive for both EBV and CMV. Initial studies using this inoculum had initially required that participants in CHMI trials be seropositive for both infections. However, following the development of PCR assays for both infections, molecular analysis of the inoculum was negative for both EBV and CMV. Following a risk-assessment QIMR, Australia, subsequently removed seropositivity to these viruses as a criterion for participation as a recipient of the inoculum. In a previous *P. falciparum* challenge study (VAC054) at Oxford, 63% of volunteers were seronegative for CMV and 11% were seronegative for EBV pre-CHMI, and no cases of seroconversion were recorded post-CHMI [22]. Volunteers will however be informed of the theoretical risk of transmission of infection and this will be included within the consent form. Serostatus will be tested prior to CHMI at screening for volunteers as part of this protocol, however, this solely relates to interest in the effect on the immunological response. Serum samples collected after each CHMI will be tested for for serostatus analysis to HIV, Hepatitis B, Hepatitis C, EBV and CMV to confirm lack of transmission of blood-bourne viruses. Serum samples post-CHMI will also be stored in case further analysis of serostatus is required or requested by the Chief Investigator subsequently.

5.7.5 Sterility and screening for blood-borne infections of cryopreserved blood bank

Blood collected for cryopreservation was tested for bacterial contamination using validated GMP or GLP laboratory techniques. These included tests of sterility by direct inoculation, mycoplasma specific culture and detection and quantification of bacterial endotoxin by kinetic chromogenic limulus amoebocyte lysate assay. All of these assays were performed by SGS Vitriology, Glasgow, UK, using assays compliant with harmonised European Pharmacopeia and United States Pharmacopeia standards.

A further screen for blood-borne infections is being conducted on the plasma, derived directly from the blood bank (separated from the red cells prior to freezing down), in line with testing procedures performed by the NHS Blood Transfusion service. DNA PCR for HIV-1, hepatitis B, hepatitis C, EBV, CMV, serology for HIV-2 and serology for HTLV-1, HTLV-2, and *Treponema pallidum* (RPR) will be performed on thawed plasma samples at Kings College Hospital NHS Foundation Trust laboratory.

We intend to proceed to human challenge if all the above screens for contamination and infection are negative, with the exception of PCR for CMV or EBV. In the event that DNA PCR for CMV or EBV is positive, volunteers for this study will only be enrolled if they are also seropositive for these infections, i.e. are seroconcordant.

5.8 Source and preparation of the *P. falciparum* cryopreserved inoculum

The *P. falciparum* infectious inocula to be used in VAC069E were produced by Drs Gregor Lawrence, Allan Saul and colleagues at QIMR in Brisbane, Australia in 1994 [54]. The protocol for the study was reviewed and approved by the QIMR Ethics Committee and the Healthy Volunteer Studies Research Ethics Subcommittee, Lothian Health Board (Edinburgh). Procedures were designed to minimise the risk of other infectious agents in the cryopreserved samples.

5.8.1 Collection and cryopreservation of inoculum

Laboratory-reared *Anopheles stephensi* mosquitoes were infected with the *P. falciparum* clone 3D7 (a chloroquine-sensitive strain) by membrane feeding on a blood meal containing gametocytes. Ten and fourteen days later, the mosquitoes were fed on two volunteers. Parasitaemia in the volunteers was followed by daily microscopy from day 4 after infection. Blood

was taken and frozen in aliquots from the volunteers 6 hours after they developed fever, when both were microscopically parasite positive. The volunteers were treated with chloroquine soon after blood was drawn with complete recovery.

Initial development of the blood inoculum to be used in this study and its use in five volunteers is described by Cheng *et al.* 1997 [54]. The inoculum used for all volunteers comes from one of the donors described above. This is because the second donor (whose blood has not been used) had a much lower parasitaemia [55]. Blood was collected at the Australian Red Cross Blood Bank in an aseptic manner using standard blood bank equipment. The leukocytes were removed with a leukocytic filter. The thawing and washing of the cells reduced the amount of serum transferred with the red cells by a factor of 1000, compared to injecting the same volume of blood. The volume of inoculum to be given to each volunteer contains a very small volume of red blood cells, equivalent to only 1.5 to 4 microlitres of blood.

The red cells were cryopreserved using a protocol from the American Association of Blood Banks Technical Manual that is normally employed for freezing blood from patients and donors with rare blood groups. Blood from both volunteers was group O and Rhesus negative.

Viability of the inoculum was confirmed by culture in a trial in Nijmegen in 2011 [56]. For each vial of inoculum used, a small amount will be cultured after inoculations are complete to confirm viability and the estimated number of parasitised red blood cells inoculated.

Between 1994 and 2003 the cryopreserved samples to be used in this trial were stored in dedicated liquid nitrogen cylinders in a secure facility at QIMR. The liquid nitrogen containers were kept locked and accessible only to approved staff. In 2003 the samples were transferred to Biotec Distribution Ltd., Bridgend, UK and then to Thermo Fisher Bishop's Stortford, Hertfordshire, UK, in 2007 where they have been stored on behalf of Oxford University in temperature-monitored liquid nitrogen.

5.8.2 Previous Studies

The blood-stage *P. falciparum* 3D7 inoculum has to date been used in over 400 volunteers in 32 studies.

Oxford University has previously undertaken six malaria challenges of healthy human volunteers using this inoculum. The first was a pilot study in 2003 in which 5 volunteers received the inoculum. As expected, the results showed less variation in rate of rise of parasitaemia than is typically experienced with a liver-stage (or sporozoite) malaria challenge. The trial was successful, with all volunteers being diagnosed with malaria between the morning of day 7 and the morning of day 9 after inoculation. The symptoms of malaria experienced by volunteers at and around the point of diagnosis were as expected. No SAEs were reported. All volunteers were clinically well after antimalarial treatment when reviewed in clinic at day 42 after inoculation. During the trial one volunteer became mildly anaemic but this was transient, with a normal haemoglobin on repeat testing one week later; and one volunteer had a raised bilirubin of 35 µmol/L on two occasions. All volunteers successfully completed follow-up [57].

Following this a Phase IIa study of the Safety, Immunogenicity and Parasite Growth Inhibitory Activity of AMA1-C1/Alhydrogel® + CPG 7909 was conducted (VAC035). This involved 8 healthy volunteers (5 vaccinees and 3 controls) receiving the blood-stage CHMI with the same inoculum. All individuals challenged developed malaria (confirmed by positive blood film and positive qPCR), although only 2 of the 8 subjects developed clinical malaria symptoms pre-diagnosis [58]. A significant inverse relationship between vaccine-induced growth inhibitory activity (GIA) and PMR was seen in vaccinated subjects, but no impact on overall PMRs was observed across the

vaccinated and control groups. However, the sample size in this study was very small, limiting the statistical power to assess differences in PMR between the groups [58].

VAC054 next looked at the safety, immunogenicity and efficacy of the AMA1 vaccine candidate FMP2.1 given with the AS01 Adjuvant System. In this trial 12 vaccinated volunteers and 15 infectivity controls received the blood-stage CHMI inoculum, which was homologous to the vaccine. The vaccine induced functional antibodies which were able to inhibit growth *in vitro* in a growth inhibition activity (GIA) assay, but demonstrated no efficacy with all vaccinees and controls developing blood-stage malaria. There was no effect on PMR in vaccinees compared with the infectivity control group. However, this trial demonstrated the reproducibility of the blood-stage CHMI model, with much larger group sizes than used in previous studies. The study also demonstrated its utility for measuring modest reductions in PMR in comparison to mosquito-bite CHMI where analysis of historical data showed a higher dispersion of data in the infectivity controls [22].

VAC063 looked at the safety, immunogenicity and efficacy of the blood-stage vaccine candidate RH5.1 given with the AS01 Adjuvant System. Fifty healthy adult volunteers were enrolled into the trial and the RH5.1/$AS01_B$ formulation was administered using a range of RH5.1 protein vaccine doses (2, 10, and 50 µg) and was found to be safe and well tolerated. A regimen using a delayed and fractional third dose, in contrast to three doses given at monthly intervals, led to significantly improved antibody response longevity over ∼2 years of follow-up. Following primary and secondary CHMI of vaccinees with blood-stage *P. falciparum*, a significant reduction in parasite growth rate was observed, defining a milestone for the blood-stage malaria vaccine field. The trial also demonstrated that growth inhibition activity (GIA) measured *in vitro* using purified immunoglobulin G (IgG) antibody strongly correlates with *in vivo* reduction of the parasite growth rate [59].

VAC063C was a follow-on study to VAC063 to assess repeat challenge with *P. falciparum*. 6 volunteers previously enrolled in VAC063 underwent tertiary challenge, 2 volunteers previously enrolled in VAC063 underwent secondary challenge and 3 volunteers underwent primary challenge. No SAEs related to the malaria challenge occurred during the trial. Re-challenge resulted in no anti-parasitic immunity and no obvious change in the clinical symptom profile of participants. Two volunteers undergoing primary challenge developed transaminitis, one of whom had severe elevation of ALT and GGT and was mildly symptomatic. Coagulation and bilirubin remained normal and the transaminitis resolved spontaneously within 4 weeks. However, there was barely any deviation from baseline in liver function tests in those participants undergoing a secondary or tertiary infection, and this was independent of parasite load. This may indicate that the risk of tissue damage and injury can be reduced in the absence of parasitic control (Sandoval *et al.,* unpublished data; Themistocleous *et al.,* unpublished data).

5.9 Conduct of CHMI trials

Following a collaborative consensus process involving investigators from the USMMVP, Sanaria, University of Maryland, University of Oxford, RUNMC, The Seattle Biomedical Research Institute and the KEMRI-Wellcome Kilifi Research Programme, a consensus document; *"Standardization of Design and Conduct of P. falciparum Sporozoite Challenge Trials"* was developed, and provides a comprehensive guide to the appropriate conduct of sporozoite CHMI studies [36]. There is no equivalent document for controlled blood-stage infection or for *P. vivax* challenge, however, this study will be conducted in line with the following key points.

- All volunteers should have a medical assessment no longer than 48 hours before challenge, including an interim medical history, directed physical examination, pregnancy test for female volunteers.
- Volunteers should be questioned about the occurrence of adverse events and use of medication at each follow-up visit.
- In the event that a volunteer does not attend a scheduled follow-up visit it is imperative that Investigators find that volunteer as quickly as possible and assess them for patent parasitaemia and clinical malaria. Should the volunteer withdraw consent from further follow-up prior to receipt of antimalarial drugs, it may be appropriate to withdraw the volunteer from the trial protocol and administer a course of antimalarial chemotherapy under close supervision.
- Grading and reporting of adverse events should be performed using international and local guidelines. It should be noted that the occurrence of a low frequency of grade 3 severe adverse events, of short duration, and with no long-term sequelae, is not unexpected in CHMI studies. A minority of those challenged are known to experience grade 3 systemic adverse events and this fact should be included in the informed consent form.
- Vital signs should be recorded at least once daily from day 6 post-CHMI (or sooner if any clinical concerns) and at any subsequent visits for medical attention. Directed physical examination should be performed when necessary.
- It is critical that every volunteer must receive every dose of anti-malarial therapy. In some settings fully directly observed treatment will be essential. Where directly observed treatment is not used, Investigators must follow volunteers closely to ensure compliance with the treatment regimen.
- After challenge, all volunteers should be followed until they have completely finished anti-malaria treatment.
- Volunteers should be evaluated at least two weeks after finishing treatment.
- A local safety monitor and an independent safety monitoring committee should be established to act as independent experts in evaluating adverse events. The safety monitor or monitoring committee may advise the Investigators on initiating anti-malarial treatment for a specific volunteer or volunteer group. While safety monitoring committees are not a requirement for Phase I trials, they should be considered a requirement for CHMI trials which have an efficacy/human challenge component and which have major potential safety concerns.

5.9.1 Clinical presentation post-CHMI

Nearly all unvaccinated volunteers in CHMI studies develop symptoms of clinical malaria infection; based on data predominately from *P. falciparum* challenge studies approximately one-fifth of volunteers temporarily develop symptoms graded as severe (symptoms that prevent daily activities), but severe or life-threatening malaria has never occurred [60]. The expected time-course, symptoms and management of clinical malaria are outlined in Section 9.

Routine laboratory checks generally show a moderate decrease in leukocyte and platelet numbers during infection, with no change in haemoglobin concentration [61]. Bleeding or thrombogenic complications have never been described [60, 61]. Abnormalities of liver enzymes have been observed, but these abnormalities have rarely resulted in clinical manifestations (just one volunteer with raised ALT associated with abdominal pain and vomiting in the recent RCT from Cali [44]) and they resolved after a few days.

As of 2011, human malaria challenge infections have been conducted with *P. falciparum* in over 1,300 volunteers [60-62]. In 2009, safety concerns were raised when young volunteer suffered a cardiac event shortly after treatment for diagnosed malaria, following a *P. falciparum* challenge. This was diagnosed as probable myopericarditis, although ischaemia could not be ruled out. Although a definite relationship between the cardiac event, which resolved fully and rapidly, and the experimental malaria infection was not established [63], it has been generally agreed that volunteers with an increased risk of cardiac disease should be excluded from such trials [36]. A further case of myopericarditis has since been identified in a recent *P. falciparum* CHMI study, also at the Nijmegen, Netherlands centre, but in this case the individual was also diagnosed with an intercurrent rhinovirus infection so that the relation to malaria infection is again uncertain. There was a brief episode of clinical chest pain and the volunteer made a full recovery [64].

5.9.2 Ethical considerations of CHMI trials

Participants in CHMI trials are healthy volunteers who do not obtain direct health benefit from participation. Challenge trial investigators must exercise all possible safeguards for volunteer safety to ensure that trial participation is of minimal risk. Investigators must also ensure that maximal scientific benefit accrues from each challenge trial. Key ethical considerations agreed by consensus of the field are outlined in a review by Moorthy from 2011, and include; [36]

1. Volunteer safety is the paramount consideration in conduct of CHMI trials.
2. Adherence to both international and local guidelines with respect to ethical considerations and in accordance with the Declaration of Helsinki and local regulatory and ethics committee requirements.
3. CHMI trials should be conducted according to ICH and/or WHO Good Clinical Practice Guidelines with the aim of maximising scientific benefit whilst minimising risk.
4. The raw data (both microscopy and PCR where available) from challenge trial datasets should be made publicly available to facilitate scientific benefit to the community.
5. If an unexpected SAE which is possibly related to CHMI occurs at a challenge trial centre, recognising legal restrictions, every effort should be made to communicate information on this SAE to the community of challenge trial centres within 90 days of the occurrence of the SAE.

5.9.3 Oxford's experience conducting CHMI trials

The University of Oxford has been conducting CHMI studies (with *P. falciparum*) for the last 14 years. To date, more than 400 volunteers have undergone CHMI in studies conducted by the University of Oxford, including more than 100 unvaccinated control volunteers. An analysis has shown that the symptoms of malaria experienced by volunteers undergoing sporozoite challenge by mosquito bite in Oxford are broadly similar to those experienced by volunteers challenged by mosquito bite at Radboud University Nijmegen Medical Center (RUNMC) and the US Military Malaria Vaccine Program (USMMVP) [65]. In Oxford, four volunteers amongst >400 individual challengees have required admission to hospital for observation following challenge (SAE related to CHMI). All of these subjects made a complete recovery. Oxford has never before conducted a blood-stage *P. vivax* challenge study, but we have based our study design on that of the previous *P. vivax* blood-stage challenge studies in Australia, combined with our own extensive experience of *P. falciparum* sporozoite challenge studies and our recent VAC068 *P. vivax* sporozoite challenge study.

In one Phase I/IIa sporozoite challenge study assessing the efficacy of viral vectored malaria vaccines in Oxford (VAC039; Clinicaltrials.gov reference: NCT01142765), a volunteer who underwent sporozoite challenge on 1st October 2010 failed to attend his next scheduled study

visit on 7th October 2010 [66]. The police were immediately informed and began a nationwide search for the individual. All volunteers had been informed at screening that the police would be notified should they go missing following CHMI if they had not completed a full course of an appropriate anti-malarial treatment. The volunteer was found in the Netherlands by the local police 17 days following CHMI. He then had very mild malaria symptoms. He was admitted to a local hospital where he received appropriate treatment for *P. falciparum.* He had no signs of severe malaria but showed an altered mental state considered unrelated to malaria with apparent memory loss and suicidal ideation. He was therefore transferred for in-patient psychiatric assessment and discharged a few days later to his GP's care. He has subsequently been reportedly diagnosed as schizophrenic.

It emerged that from 2nd October 2010, the day after CHMI, the volunteer had experienced an alteration in his expected behaviour following his arrest the previous evening by the police relating to their investigation of a serious crime, of which he was later convicted. This arrest appeared to trigger his leaving home and disappearance and appears relevant to the subsequent finding of memory loss and suicidal ideation. It emerged on subsequent investigation that the volunteer actually had a history of some psychiatric morbidity pre-dating his involvement in the study by many years, which was not disclosed at screening by the volunteer or his GP. Of note, the volunteer had attended 9 clinic visits prior to challenge and appeared a reliable and appropriate volunteer.

This event was extensively discussed with investigators, colleagues and appropriate authorities and non-study related causality agreed. Given the exceptional circumstances relating to this case, it seems very unlikely that a similar event would happen again in the future. Management of the event was extensively reviewed by the trial's sponsor, the MHRA and ethical committee who felt that appropriate and timely action was taken. Follow-up procedures following CHMI have been reviewed locally, and it has been decided that for future studies volunteers should be contacted daily on days 1-5 post sporozoite challenge in order to make sure they are contactable and well. Blood-stage challenge model volunteers are seen from Day 1 post-challenge and therefore followed even more closely initially.

5.9.4 Conducting CHMI in the context of the COVID-19 pandemic

Due to the COVID-19 pandemic this trial was temporarily halted in March 2020. The incidence of COVID-19 continues to change in England and some ongoing transmission is likely to remain when this trial restarts in 2021 [67]. Each phase of this malaria challenge trial will only proceed, when rates of COVID-19 infections in England are low and the risk of contracting COVID-19 during malaria challenge is small, however the risk cannot be eliminated. There is currently no evidence for the possible effects of co-infection with malaria and COVID-19. As the risk of co-infection cannot be quantified, participants will be tested for COVID-19 prior to malaria challenge and any participant that tests positive for COVID-19 will not proceed with malaria challenge.

Participants who test positive for COVID-19 will be advised to self-isolate as per UKHSA guidance and directed to local NHS services for further care if required. Positive COVID-19 PCR results will be reported as a notifiable disease as per UKHSA guidance.

Fevers which occur following malaria challenge may pose diagnostic difficulties as concurrent COVID-19 infection will likely remain an ongoing possibility in England. Data from the 12 primary and 4 secondary CHMI with *P. vivax* conducted in Oxford to date, show that no participants developed symptoms that are consistent with malaria within the first week post challenge. Pyrexia or subjective feelings of feverishness occurred in only half of the participants prior to diagnosis and treatment of malaria. However the majority of remaining participants who were initially apyrexial, did subsequently develop fever after commencing malaria treatment, although

this was short lived and resolved within 2 days. Following malaria challenge, participants first developed pyrexia or subjective feelings of feverishness between days 10 to 20 post challenge (median 14 days), with corresponding malaria qPCR levels of 100 to 9600 genome copies/mL (median 2700 genome copies/mL). No recorded pyrexia of >37.8°C occurred at a malaria qPCR below 1000 genome copies/mL in any participant. Malaria diagnosis was made in participants within a few days following the onset of symptoms with some participants reporting no symptoms prior to laboratory diagnosis. Participants were diagnosed with malaria based on a combination of clinical symptoms, malaria qPCR and thick film microscopy between days 12 and 21 post challenge (median 15.5 days).

Fevers within the first week after malaria challenge are therefore unlikely to be due to malaria. After the first week post challenge, malaria as the cause of fever becomes increasingly likely with increasing time since challenge. However, as concurrent COVID-19 infection will likely remain a possibility, testing for COVID-19 has been added in the post-challenge period for participants who become febrile. For participant safety, if a participant tests positive for COVID-19 prior to malaria diagnosis, they will be treated for malaria regardless of if they have reached the criteria for malaria diagnosis.

There is no evidence for any detrimental effect of anti-malarial medication on the outcome of concurrent COVID-19 infection. This is in the context of widespread antimalarial use during the COVID-19 pandemic in Africa, where no safety signal has emerged. Recent randomised controlled trials have shown no benefit of Hydroxychloroquine in COVID-19 [68], but also no harm, and Chloroquine will not be used for malaria treatment in this trial. The anti-malarial medications Riamet and Malarone are not expected to have any effects on the outcome of COVID-19 disease.

Social distancing requirements to control COVID-19 will likely remain in place to some extent in England. To abide with these, participants' attendances at study clinics will be carefully planned to adhere to social distancing guidelines. Participant flow will be arranged so that contact with other people is minimised and level 1 Personal Protective Equipment (PPE - compromising apron, gloves, surgical mask and eye protection) will be worn by members of clinical staff for all participant visits. This will follow an infection control SOP that covers all PPE requirements for clinical trials at the Oxford Vaccine Centre in the era of the COVID-19 pandemic.

As the national rollout of COVID-19 vaccines proceeds, some participants may be offered a COVID-19 vaccine around the time of planned malaria challenge. It is unknown what effect concomitant COVID-19 vaccine administration around the time of CHMI may have on the immune response to malaria infection, which is being studied in this trial. In addition reactogenicity from COVID-19 vaccination may be difficult to distinguish from symptoms of malaria and may lead to earlier diagnosis and treatment of malaria after challenge. Therefore concomitant COVID-19 vaccination around the time of CHMI has been added as an exclusion criteria in this trial.

6 DESCRIPTION AND JUSTIFICATION OF STUDY DESIGN

6.1 Overview

This is a blood-stage *P. vivax* human challenge study with the primary aim of assessing the safety and feasibility of the challenge model using this bank of cryopreserved *P. vivax* infected erythrocytes for the first time. This will be assessed in VAC069A at three different doses of inoculum, by injection at three dilutions, with a view to identifying the lowest concentration producing a reliable infection within a practicable timeframe. A secondary objective is to determine the safety and feasibility of secondary and tertiary homologous controlled blood-stage *P. vivax* infection. If safe and feasible, this study will serve as the basis for a challenge model for future *P. vivax* candidate vaccine efficacy studies, as well as re-challenge studies without vaccination.

Healthy, malaria-naïve adults aged between 18 and 50 years will be recruited at the CCVTM, Oxford. CHMI will be induced by injection of *P. vivax* infected erythrocytes and all follow-up in the post-challenge period will be performed at the CCVTM in Oxford.

Volunteers will have blood taken at regular intervals post-CHMI to assess the immune response to primary, secondary and tertiary *P. vivax* infection, as well as parasite growth dynamics and gametocytaemia. Depending on the observed gametocytaemia and availability of laboratory strains of the appropriate *Anopheline* mosquito vectors, we will also assess transmission of *P. vivax* gametocytes from the infected volunteers to mosquitos. If performed, this will be done by taking blood for Direct Membrane Feeding Assays (DMFA) and/or by direct feeding of the mosquitos on the volunteers' skin at, or as close as possible to, peak gametocytaemia.

Close monitoring will continue until volunteers meet criteria for treatment or until 28 days after challenge, when treatment will be started empirically.

Therapy will be with either a standard 60-hour course of oral artemether-lumefantrine (Riamet®) or 3 day course of oral atovaquone-proguanil (Malarone). As infection will be induced via intravenous injection of blood-stage parasites, there will be no liver-stage infection and no hypnozoite formation, thereby eliminating need for radical cure with primaquine therapy. Follow-up will be for up to 96 days after the last challenge via clinic visits.

In the final phase of the study VAC069E, volunteers who have previously undergone primary or secondary challenge with *P. vivax* will undergo a repeat challenge with *P. falciparum*. The aim of this is to study if the development of clinical immunity following primary infection with *P. vivax* also extends to another *Plasmodium* species ie *P. falciparum*.

6.2 Study groups

Up to twenty-four volunteers will be recruited through the five phases of the study VAC069A-E (see Table 4). Each stage of study (VAC069A-E) comprises a CHMI, involving up to three groups, and will take place sequentially at intervals of five to eighteen months, as described below. A new group of volunteers will be recruited at each stage (VAC069A-D) to undergo primary CHMI in parallel with secondary or tertiary CHMI of volunteers already enrolled in the study. VAC069E will not recruit new volunteers. Instead, volunteers who completed at least one previous CHMI with *P. vivax* during VAC069D will be re-enrolled. Recruitment for VAC069A-D is summarised as follows:

VAC069A: 6 volunteers recruited into Groups 1-3 (plus two back-up volunteers)

VAC069B: 2-3 new volunteers recruited into Group 6 (plus one back-up volunteer)

VAC069C: 4-8 new volunteers recruited into Group 9 (plus up to four back-up volunteers)

VAC069: A study of blood-stage controlled human *Plasmodium vivax* malaria infection. IRAS project ID: 252499

VAC069D: 4-8 new volunteers recruited into Group 12 (plus up to four back-up volunteers)

Back-up volunteers may take the place of another volunteer, should they withdraw or become ineligible prior to challenge.

When first enrolled in the study, volunteers will be recruited into either Groups 1-3, 6, 9 or 12 and will undergo primary CHMI. Following primary CHMI, enrolled volunteers will enter a new group and partake in a new phase of the study, as follows: volunteers initially in Groups 1-3, will enter Group 4 (secondary CHMI) and subsequently, Group 5 (tertiary CHMI); volunteers recruited into Group 6 will enter Group 7 (secondary CHMI) and then Group 8 (tertiary CHMI); volunteers recruited into Group 9, will subsequently enter Groups 10 (secondary CHMI). Volunteers in Groups 10 and 12 who completed at least one CHMI with *P. vivax* during VAC069D will be re-enrolled into Group 13 to undergo a heterologous re-challenge with *P. falciparum*.

During phases VAC069B-D, recruitment will only be open to one new group per phase of the study, therefore, volunteers will not be able to choose the group to which they are allocated.

In March 2020 due the COVID-19 pandemic, this trial was temporarily halted. At the time of trial halt VAC069A and VAC069B had been completed. Participants in Groups 1-3 had completed their primary CHMI as part of VAC069A and these participants formed Group 4 who underwent secondary CHMI as part of VAC069B. Participants in Group 6 had completed primary CHMI as part of VAC069B.

On restarting the trial no new participants will be recruited into the groups from VAC069A and B, only those participants who have already undergone primary or secondary CHMI as part of VAC069A and VAC069B will continue to undergo secondary and tertiary CHMI respectively. VAC069C will be delayed by about twelve months. VAC069C Group 9 will be reduced in size for logistical reasons to ensure compliance with social distancing. To compensate numbers and ensure we have enough volunteers going through three successive challenges, VAC069D Group 12 has been increased in size and this group will now subsequently undergo secondary and tertiary CHMI, in addition to the originally planned primary CHMI. However, the increased size of this group will not affect the total number of participants undergoing challenge at the same time as the VAC069D phase of the study because the number of vaccinees from VAC071 and VAC079 studies, who will be undergoing challenge in parallel to VAC069D, will be reduced.

Following completion of the CHMI in VAC069D in October 2021, results from phases of the study completed to date indicated that no anti-parasitic immunity develops upon re-challenge with the same clonal *P. vivax* inoculum. However, clinical immunity developed after the first infection and upon homologous secondary challenge, volunteers had reduced clinical symptoms and signs of malaria. In order to study if the clinical immunity that develops following primary infection with *P. vivax* also extends to other *Plasmodium* species, repeat challenge will be conducted with *P. falciparum*. In the next phase VAC069E, volunteers who completed at least one CHMI with *P. vivax* in VAC069D will be re-enrolled and undergo a final CHMI with *P. falciparum*.

6.2.1 VAC069A Initial challenge and dose-finding: Groups 1 (n=2), 2 (n=2) and 3 (n=2).

Six volunteers will be recruited into three groups, with two back-up volunteers who may take the place of a volunteer in any group, should they withdraw or become ineligible prior to challenge. Should they wish, volunteers will be able to choose the group to which they are allocated, provided the group has not yet been filled. Otherwise, volunteers will be allocated to groups on a sequential basis, from Group1 to Group 3.

Each group will comprise two volunteers. All groups will receive an inoculum of parasitised red blood cells, reconstituted in 0.9% normal saline, to a total volume of 5mL. Group 1 will receive a

whole vial's worth of infected erythrocytes (starting total volume 1.5mL, containing 0.5mL packed red blood cells and 1mL Glycerolyte – these cells are thawed and extensively washed to remove Glycerolyte prior to resuspension in saline). Group 2 will receive one fifth of the dose of parasitised erythrocytes, at a 1:5 dilution in 0.9% normal saline, and Group 3 will be inoculated with one twentieth of the dose, at a 1:20 dilution in 0.9% normal saline. This will constitute the primary blood-stage challenge. Later challenges will be conducted at the lowest concentration proving potency and feasibility within this first phase of the study (see Tables 3 and 4).

6.2.2 VAC069B Secondary re-challenge with new primary controls: Groups 4 (n=6) and 6 (n=2-3).

Approximately eight months (and up to nine months) later, the six volunteers from Groups 1, 2 and 3 in VAC069A will undergo secondary challenge using the optimal inoculum as determined in that first phase of the study. They will constitute 'Group 4' in this phase of the study. Three new malaria naïve volunteers, as well as a one volunteer who will act as a back-up, will be recruited (Group 6) to undergo primary challenge and act as controls to Group 4. The three malaria-naïve volunteers in Group 6 will also act as the non-vaccinated control group for three vaccinated volunteers in a parallel vaccine efficacy trial, known as VAC071.

VAC071 will be a Phase IIa study to assess efficacy of the chimpanzee adenovirus serotype 63 (ChAd63)/modified vaccinia virus Ankara (MVA) viral-vectored vaccine candidate encoding the *P. vivax* Duffy-binding protein region II (PvDBP_RII), in a heterologous prime-boost regimen. The ChAd63-PvDBP/MVA-PvDBP vaccine candidate has proven safety and tolerability, as well as induction of strain-transcending antibodies in an open-label dose-escalation Phase Ia study [37].

6.2.3 VAC069C Tertiary (n=6) and secondary (n=2) re-challenges with new primary challenge controls (n=4-8): Groups 5, 7 and 9.

Approximately sixteen to twenty months later any volunteers remaining in the study from Group 4 in VAC069B will undergo tertiary challenge, again using the optimal inoculum as determined in VAC069A and will constitute Group 5 in this third phase of the study. Any remaining volunteers who underwent primary challenge in Group 6 will now form Group 7 and undergo secondary challenge. Four to eight new malaria-naïve volunteers will be recruited (Group 9) and act as controls for Groups 5 and 7 and for vaccinated volunteers undergoing primary CHMI as part of VAC071 and VAC079. In addition, up to four volunteers will be recruited to act as back-ups for Group 9.

VAC079 will be a Phase IIa study to assess the safety and efficacy of the protein subunit vaccine PvDBP_RII administered with the adjuvant Matrix M. The safety and immunogenicity of PvDBP_RII has previously been shown with adjuvant GLA-SE (made by IDRI, Seattle, USA) in a Phase Ia clinical trial in India, and induces strain-transcending functional antibodies [69]. The safety profile of Matrix M with other malaria protein subunit vaccines is excellent, as shown in a previous clinical trial [70] but it has not yet been tested in combination with PvDBP_RII in humans. Challenge of vaccinated volunteers in the VAC071 and VAC079 studies will occur in parallel for comparative assessment of vaccine efficacy. Where consent is obtained, the data generated from the challenge of malaria-naïve controls within the VAC069 study will be utilised on an anonymous basis, for comparative analysis with the challenge of vaccinated volunteers in VAC071 and VAC079.

Conduct of secondary and tertiary CHMI in parallel with these control groups, will permit direct comparison across the two, or three, groups respectively, allowing adequate assessment of safety and feasibility of secondary and tertiary challenge, as well as immune responses and gametocytaemia during secondary and tertiary CHMI.

6.2.4 VAC069D Tertiary (n=2) and secondary (n=4-8) re-challenges with new primary challenge controls (n=4-8): Groups 8, 10 and 12.

Between five to ten months later the remaining volunteers from Group 7 in VAC069C will undergo tertiary challenge and constitute Group 8 in this fourth phase of the study. The four to eight volunteers from Group 9 in VAC069C will undergo secondary challenge and form Group 10 in this phase of the study. Four to eight new malaria-naïve volunteers will be recruited to form Group 12 and undergo primary CHMI. They will act as naïve controls for Groups 8 and 10 and as controls for vaccinated volunteers undergoing primary CHMI as part of VAC071 and VAC079. Up to four back-up volunteer will also be recruited for Group 12.

6.2.5 VAC069E Re-challenge with *P. falciparum*: Group 13.

In this final phase of the study scheduled for eight to fourteen months after VAC069D, volunteers in Groups 10 and 12 who completed at least one *P. vivax* CHMI in VAC069D will be re-enrolled into Group 13 to undergo repeat CHMI with *P. falciparum* instead. There will be no volunteers undergoing primary challenge in this phase.

Month (approx.)		0	9	26	34	40
Months between CHMI		0	9	16-24	5-10	8-14
Phase of Study		A	B	C	D	E
Group	Group size					
Group 1	2	1° CHMI: 1 vial				
Group 2	2	1° CHMI: 1:5				
Group 3	2	1° CHMI: 1:20				
Group 4 (previously groups 1-3)	3		2° CHMI			
Group 5 (previously group 4)	1-2			3° CHMI		
Group 6	2		1° CHMI			
Group 7 (previously group 6)	1-2			2° CHMI		
Group 8 (previously group 7)	1-2				3° CHMI	
Group 9	4-8			1° CHMI		
Group 10 (previously group 9)	4-8				2° CHMI	
Group 12	4-8				1° CHMI	
Group 13 (previously groups 10 and 12)	4-12					***P. falciparum*** **CHMI**

Table 3: Study groups. *CHMI = controlled human malaria infection. Parasitised red blood cells will be reconstituted to a total volume of 5mL normal saline (0.9%) (one inoculum vial contains approximately 0.5mL cryopreserved packed red blood cells). Secondary and tertiary CHMI will take place between approximately 5 to 14 months after the preceding challenge. CHMI during phases B, C and D will take place in parallel with primary challenge of malaria naïve vaccinated volunteers recruited as part of the VAC071 and VAC079 studies.*

Phase of study	Month (approx.)	Max Total participants	Groups (previous Group)	Group size	Intervention
**VAC069A**	**0**	**6**	**1**	**2**	**1° CHMI**
			**2**	**2**	**1° CHMI**
			**3**	**2**	**1° CHMI**
**VAC069B**	**9**	**9**	**4 (previously 1-3)**	**6**	**2° CHMI**
			**6**	**2-3**	**1° CHMI**
**VAC069C**	**26**	**12**	**5 (previously 4)**	**1-6**	**3° CHMI**
			**7 (previously 6)**	**1-2**	**2° CHMI**
			**9**	**4-8**	**1° CHMI**
**VAC069D**	**34**	**18**	**8 (previously 7)**	**1-2**	**3° CHMI**
			**10 (previously 9)**	**4-8**	**2° CHMI**
			**12**	**4-8**	**1° CHMI**
**VAC069E**	**45**	**12**	**13 (previously 10 and 12)**	**4-12**	**Repeat CHMI with *P. falciparum***

Table 4: Study phases. *CHMI = controlled human malaria infection. Repeat CHMI for Groups 4-13 will take place approximately 5 to 14 months after the preceding challenge.*

6.3 Duration of volunteer participation

The duration of involvement in the study, from enrolment until the end of the follow-up following the third CHMI, will be approximately 30 months for Groups 1-8. This has been extended by approximately 12 months because of the temporary trial halt due to the COVID-19 pandemic. Duration of involvement for participation in groups 9-12 who are undergoing three CHMI will be approximately 18 months. Follow-up for participants in group 13 who will undergo repeat CHMI with *P. falciparum* will be 3 months. All volunteers will be followed for 3 months after their last challenge.

6.3.1 Definition of the start and end of the trial

The start of the trial is defined as the date of the site initiation visit (SIV). The end of the trial is the date of the last follow-up of the last volunteer.

6.4 Potential risks for volunteers

6.4.1 Phlebotomy

The maximum volume of blood drawn over the study period should not compromise these otherwise healthy volunteers. There may be minor bruising, local tenderness or pre-syncopal symptoms associated with venepuncture, which will not be documented as AEs if they occur. The total volume of blood taken over the study period will relate to the number of challenges

participated in by the volunteer. Volunteers will have a maximum of 1042 mL taken for each challenge taken over the challenge period (minimum 96 days). At each further challenge, a maximum of 1042mL would be taken, beginning at least five months after the preceding challenge. Male regular blood donors may donate one unit (470mL) every 12 weeks and females every 18 weeks. However, a recent multicentre study by NHS Blood and Transplant (in Oxford and Cambridge) compared outcomes in 45,000 regular blood donors who were randomised to different intervals between blood donations (as regularly as giving 470 mL every 8 weeks in male participant group and every 12 week in females) over 2 years[71]. There were no significant differences were observed in quality of life, physical activity, or cognitive function although there were more symptoms related to blood donation, in groups donating more frequently, including tiredness, breathlessness, feeling faint, dizziness and restless legs. Symptoms were most increased among male participants. Lower mean haemoglobin and ferritin concentrations were also detected.

In this study, participants will never donate 470mL of blood in one sitting; the maximum volume they will donate in a single visit is 149mL so we would not expect them to report a high frequency of symptoms at the time of or just after blood donation. They will be closely monitored at all times of blood donation, and haemoglobin will be checked regularly, as described in the study procedures. Any abnormal result will be re-checked and referred to the GP for further investigation and management, as deemed clinically appropriate by the Investigator.

The maximum total blood volume drawn in this study will be 2971mL over approximately 18 months. For male participants, this is 1259mL less than the 8-week interval group in the NHSBT study and for females, 151mL more than the 12-week interval group, over the 18 month period. However, this maximum volume assumes that each participant is not treated until C+28 for each of the three challenges they participate in. In practice, given volunteers are malaria-naïve at enrolment they are likely to be diagnosed between C+14 and C+16 in their primary challenge. This means the blood volume scheduled to be collected between C+17 and C+28 (192mL) is unlikely to be drawn. Therefore, we are confident that the actual volume of blood collected during the study period should not compromise our volunteers. However, the additional effect of malarial infection on potential anaemia is acknowledged. For this reason, the full blood count will be closely monitored throughout the study period. If a participant develops anaemia or there is any clinical concern, they will be reviewed by a physician and iron studies may be performed. Participant safety will be of paramount concern, and if there is any concern that a volunteer cannot safely tolerate the planned blood draws, the volume of blood collected from them may be reduced or the participant withdrawn from the study.

6.4.2 Risk of Infection with Blood Borne Organisms

This controlled blood-stage challenge will involve a blood transfusion, albeit of very small volume relative to standard blood transfusion. As for any transfusion, the risk of transmission of a blood borne infection cannot be completely eliminated. However, in order to minimise the risk, extensive screening for blood borne infections has been performed.

Regarding the *P. vivax* blood bank, both direct blood donors, as well as the initial source patient in Thailand, who provided blood for the mosquito infection by direct membrane feeding have undergone testing for blood borne infections. For viral infections and syphilis, testing was either serological or by nucleic acid amplification methods, in accordance with the Joint UK Blood Transfusion and Tissue Transplantation Services Professional Advisory Committee guidelines. The source patient was screened for antibodies to *W. bancrofti* at the Thai field site.

In addition, the blood bank has been subjected to direct screening and tested negative for bacterial contamination, sterility testing, endotoxin screen and mycoplasma specific culture.

Plasma derived from the bank has also been screened for blood borne infections, as detailed in Section 5.8.4.and has tested negative for all blood-borne infection screens by PCR. Further to this, as outlined in section 5.7.4, volunteers also underwent repeat serological testing for HIV, hepatitis B and C, syphilis, HITLV-1 and HTLV-2 testing was undertaken 90 days after challenge, to ensure that no seroconversion from a recently-acquired infection (that may have been undetectable around the time of challenge and therefore undetectable in the bank) had occurred since the challenge period. <u>All tests remained negative</u>. Since both donors have been resident in the UK or continental Europe in countries where Bovine Spongiform Encephalopathy (BSE) has been reported, there is a theoretical risk of transmission of variant Creutzfeldt Jacob Disease (vCJD) from the inoculum. This risk is reduced by leukodepletion, as is practised in UK blood donation, according to the Joint UK Blood Transfusion and Tissue Transplantation Services Professional Advisory Committee guidelines. Since universal leukodepletion was introduced in the UK in 1999, no cases of transmission of vCJD by transfusion have been recorded [72]. The risk of infection is further reduced by the very small size of the inoculum.

Regarding the P. falciparum blood bank, the donor was screened for a range of blood borne infections prior to the inoculum being collected, and has remained well since the first use of the inoculum in 1997 [55]. The donor tested negative for HIV, Hepatitis A, B and C, Syphilis, HTLV1 and Ross River virus. The donor was seropositive for Epstein Barr virus (EBV) and Cytomegalovirus (CMV), indicating previous infection. The blood was initially frozen for a year whilst the donor was monitored and retested. Over 430 volunteers have received the inoculum since 1997 and there have been no serious adverse events, and no cases of blood borne infection associated with this [73]. There were two individuals who were infected with *P. falciparum* malaria with the purpose of preparing the inoculum but all challenge inocula have since come from one of these donors as the other donor had a much lower parasitaemia when blood was collected.

More recently, an ampoule of the parasite seed stock from the donor was thawed and cultured within the Queensland Institute of Medical Research (QIMR) laboratory. Culture supernatant tested negative for Mycoplasma by PCR. The blood was also tested for CMV and EBV by PCR using methodologies not available at the time of the initial cryopreservation, and tested negative. Although the donor remains antibody positive for EBV and CMV, it is apparent that the risk of transmission of both CMV and EBV has been greatly reduced by leukodepletion of the donor blood (both EBV and CMV cause latent infection of leukocytes) and washing of the blood with clinical grade saline prior to use. Initially only CMV and EBV seropositive volunteers were enrolled due to a concern about the risk of transmission of these viruses, but the risk has since been deemed extremely low. The requirement for CMV and EBV seronegativity was therefore waived by QIMR and in the subsequent blood-stage CHMI trials carried out in both Nijmegen [56] and Oxford (VAC054), serostatus data were collected but not used as inclusion/exclusion criteria. None of the seronegative volunteers in VAC054 or the Nijmegen trial seroconverted during follow-up after CHMI. Subsequent Oxford CHMI studies have since stopped excluding volunteers based on EBV and/or CMV seronegativity.

The risk of transmission of variant Creutzfeldt Jacob Disease (vCJD) from the inoculum appears remote. The two donors are Australian residents, where no cases of either Bovine Spongiform Encephalopathy (BSE) in cattle or vCJD in humans have been reported to date.

The volume to be transfused for this study will be at least nine hundred times smaller than the amount received in typical blood transfusion (at the highest concentration). Serum from volunteers will be collected before and after challenge for storage.

6.4.3 *Plasmodium vivax* and *falciparum* infection

Volunteers are likely to develop symptomatic malaria infection following CHMI. Symptoms and signs will include feverishness, fever, tachycardia, hypotension, chills, rigors, sweats, headache, anorexia, nausea, vomiting, diarrhoea, myalgia, arthralgia, low back pain, thrombocytopenia and lymphopenia [65]. Unmonitored and untreated, *P. vivax* and *P. falciparum* infection can be serious and rarely fatal and, for this reason, volunteers will be followed up closely post-challenge and only enrolled in the study if they are deemed reliable and capable of complying with the intensive follow-up schedule. A very small proportion of volunteers in previous *P. vivax* blood-stage and sporozoite-stage CHMI studies have temporarily required intravenous fluid therapy for nausea and vomiting prior to treatment [44, 48]. If the Investigator judges the volunteer to be unwell enough to require intravenous fluids or continuous medical input, they shall be transferred to the John Radcliffe Hospital under the care of the NHS Infectious Diseases team until they are well enough to be discharged home.

With regards to the above, it is relevant to note that safety and parasitological data from 128 malaria-naïve subjects participating in CHMI studies in Oxford and the Netherlands were analysed and compared to a report from the US Military Malaria Vaccine Program. The authors found that cohorts with a longer prepatent period or a higher peak parasitaemia did not consistently show a higher frequency of adverse events [74]. However, these data were on *P. falciparum* challenge, and there is a lot less experience with *P. vivax* challenge.

6.4.4 Medications dispensed to volunteers in course of trial

(a) Treatment of *Plasmodium vivax* and *falciparum* Infection

P. vivax and *P. falciparum* malaria infection will be treated with either oral Riamet or oral Malarone.

Riamet contains 20mg of artemether and 120mg lumefantrine per tablet. 6 doses of 4 tablets will be given; the first dose is followed by additional doses after 8, 24, 36, 48 and 60 hours. If there is a contraindication to Riamet, oral Malarone will be used (see SmPC for side effects and contraindications to Riamet and Malarone). At a minimum, two doses (the first and third dose) of Riamet will be directly observed. The first dose will be observed in clinic and the volunteers will not return home until they are seen to be tolerating the medication.

Riamet is generally well tolerated, but may cause some side effects. Common side effects include headache, dizziness, abdominal pain and loss of appetite, sleeping problems, palpitations, nausea, vomiting, diarrhoea, pruritus, skin rash, cough, muscle or joint pain and fatigue. Volunteers will be counselled that certain side effects, for example dizziness, may impact on the performance of skilled tasks such as driving.

As Riamet may increase the QT interval, Riamet will not be administered to volunteers at risk for QT prolongation. Exclusion criteria for this study will include prolonged QT on baseline ECG, a history of long QT syndrome, a family history of congenital QT prolongation or sudden death, cardiac arrhythmias, severe heart disease, and a history of hypokalaemia or hypomagnesaemia. Volunteers will be advised to avoid grapefruit juice whilst taking Riamet as it can affect the bioavailability of the artemether [75]. Riamet is also contraindicated in volunteers using concomitant medications affecting the QT interval, and will not be given to these volunteers. Concomitant use of Riamet may decrease effectiveness of hormonal contraceptives.

Women using hormonal contraceptives will be advised to use an effective additional and/or alternative method of contraception whilst on Riamet treatment, until the start of the next menstruation after treatment.

Malarone contains 100 mg proguanil hydrochloride and 250 mg atovaquone per tablet. 4 tablets will be given once daily for 3 days. The first and second out of the three doses of Malarone will be directly observed in clinic. Prior to starting Malarone, volunteers will be screened for drug interactions and contraindications (including a serum β-hCG test in female volunteers). Malarone is generally well tolerated but may cause some side effects, most commonly headache, diarrhoea, nausea, vomiting, stomach pain, dizziness rash, fever, low mood, reduced appetite, cough or sleep disturbance.

(b) Treatment of Symptoms Associated with Malaria

Volunteers will be dispensed cyclizine and paracetamol for the treatment of symptoms associated with malaria unless there are contraindications to these medications. Cyclizine is generally well tolerated however there is a risk of an allergic reaction. Other side effects include skin rashes or itching, drowsiness, headache, dry mouth nose or throat, blurred vision, palpitations, difficulty urinating, constipation, anxiety, insomnia or hallucinations. Rare side effects include hypotonia, seizures, dizziness, hypertension, paraesthesia, jaundice, hepatitis, confusion or dyskinesias. Participants may be dispensed an appropriate, licensed alternative anti-emetic to cyclizine if they are unable to take cyclizine. Paracetamol is generally well tolerated however it can cause an allergic reaction or rarely pancytopaenia.

6.4.5 Risk of reaction to the blood sample

The donor of the blood used in the *P. vivax* inoculum is Blood Group O, Rhesus (Rh) and Kell antigen negative. People with Blood Group O negative blood are considered 'universal donors', as recipients of their blood are unlikely to develop red cell alloantibodies even when given much larger volumes of blood than is envisaged here. The Kell antigen is relatively rare in Caucasian populations, but if Kell positive red blood cells were infused into a Kell negative female volunteer, there is a theoretical risk that anti-Kell antibodies may induce foetal haemolysis in a future pregnancy. For this reason, and to avoid this risk, we ascertained that the donor blood used in these studies is Kell negative.

The donor of the blood used in the *P. falciparum* inoculum is also Blood Group O, Rhesus and Kell negative.

The maximum volume of blood to be used in this study (0.5mL packed red cells) is much smaller than that given in transfusion of one unit packed red cells (470mL). The risk of a transfusion reaction or development of antibodies to donor red cells, which may make blood transfusion more difficult in the future, cannot be completely excluded but is considered highly unlikely both because of the small volume of blood used and because the blood has been leukodepleted. Nevertheless, the volunteers will be monitored for this possibility in the period immediately after the administration of the malaria parasite dose. A baseline set of observations (respiratory rate, heart rate, temperature and blood pressure) will be taken before the administration of the inoculum. Volunteers will be reviewed 15 minutes (+/- 5 minutes) and 1 hour (+/- 20 minutes) after receiving the inoculum, with a repeat set of observations at each time point, before they are able to leave the CCVTM. The serum collected for storage (as described above) could be used to test for antibodies at a later date if deemed necessary.

6.4.6 Mosquito bites (for volunteers partaking in direct feeding assays only)

Mosquito bites may cause local inflammatory reactions with redness, itching, swelling, scaling and/or tenderness. Topical anti-histamine cream for use twice daily for 3 days post-mosquito bite will be dispensed to both volunteers on the day of mosquito feeding, according to SOP VC021: Handling, Storage and Dispensing of Non-IMP Medication. Serious allergic reactions

including anaphylaxis are rare, but may occur and for this reason volunteers will be take part in mosquito feeds in an area where Advanced Life Support trained physicians and defibrillator are immediately available. In sporozoite CHMI studies, delivered by mosquito bite, no serious allergic reactions have been seen to date. Data regarding itching and use of antihistamine cream will be collected but, as this is an expected part of the procedure, it will not be classed as an adverse event in the immediate post-challenge period.

6.5 Potential benefits for volunteers

Volunteers will not benefit directly from participation in this study. However, it is hoped that this proof-of-concept study will provide a basis for future *P. vivax* challenge studies and evaluation of candidate vaccines. Exploratory immunology performed on the volunteers' blood samples may also inform on future vaccine design. Volunteers will also receive information about their general health status.

7 RECRUITMENT AND WITHDRAWAL FOR TRIAL VOLUNTEERS

Volunteers may be recruited by use of an advertisement +/- registration form formally approved by the ethics committee(s) and distributed or posted in the following places:

- In public places, including buses and trains, with the agreement of the owner / proprietor.
- In newspapers or other literature for circulation.
- On a website operated by the Investigators' clinical trials group or with the agreement of the owner or operator (including on-line recruitment through our website).
- As a post on a Twitter or Facebook or other similar account owned and operated by the Investigators' clinical trials group.
- By e-mail distribution to a group or list only with the express agreement of the network administrator or with equivalent authorisation.
- By email distribution to individuals who have already expressed an interest in taking part in any clinical trial at the Oxford Vaccine Centre.
- On stalls or stands at exhibitions or fairs.
- Via presentations (e.g. presentations at lectures or invited seminars).
- Oxford Vaccine Centre databases: We may contact individuals from databases of groups within the CCVTM (including the Oxford Vaccine Centre database) of previous trial participants who have expressed an interest in receiving information about all future studies for which they may be eligible.

7.1 Informed consent

7.1.1 Informed consent

The information sheet will be made available to the volunteer at least 24 hours prior to the screening visit. At the screening visit, the volunteer will be fully informed of all aspects of the trial, the potential risks and their obligations. The following will be emphasised:

- Participation in the study is entirely voluntary.
- Refusal to participate involves no penalty or loss of medical benefits.
- The volunteer may withdraw from the study at any time. However if the volunteer has undergone CHMI and not completed a course of appropriate antimalarial therapy then the volunteer will need to maintain contact with the Investigators for monitoring and treatment.
- The volunteer is free to ask questions at any time to allow him or her to understand the purpose of the study and the procedures involved.
- There is no direct benefit from participating.
- The volunteer's GP will be contacted to corroborate their medical history and confirm that the volunteer is eligible to take part in the study. Volunteers will only be enrolled in the study if written information regarding the volunteer's medical history is obtained from the GP.
- The volunteer will be registered on the TOPS database (The Overvolunteering Prevention System; www.tops.org.uk).
- Blood samples will be taken and any leftover samples stored indefinitely for use in other, ethically approved research.

The aims of the study and all tests to be carried out will be explained. The volunteer will be given the opportunity to ask about details of the trial, and will then have time to consider whether or not to participate. If they do decide to participate, volunteers will be asked to complete a questionnaire testing their understanding of the trial [36]. This helps to ensure that individuals

understand the trial sufficiently to give informed consent. Provided the volunteer answers all questions in the questionnaire correctly, they will be asked to sign a consent form. A photocopy of the signed consent form will be taken and given to the participant to take away and keep and the original will be stored in the CRF. These forms will also be signed and dated by the Investigator. Volunteers who fail to answer all questions correctly on their first attempt will be allowed to re-take the questionnaire following further discussion with the Investigator. Provided they subsequently answer all questions in the quiz correctly they may then complete the consent form and be screened for the trial.

Initial informed consent will include consent for participation in up to three blood-stage CHMIs. On-going consent for participation in the study will routinely be assessed verbally at each clinical review. Given the significant time interval between challenges, prior to each of the second and third challenge, in order to identify new medical events, a re-screening procedure will be followed. At the re-screening visit, the volunteer will be reminded about all aspects of the trial, the potential risks and their obligations, with emphasis on the above listed points. The volunteer will be given the opportunity to ask about details of the trial, and will have time to consider whether or not to continue their participation in the trial.

7.2 Inclusion and exclusion criteria

7.2.1 Inclusion criteria

The volunteer must satisfy all the following criteria to be eligible for the study:

- Healthy adult aged 18 to 50 years.
- Red blood cells positive for the Duffy antigen/chemokine receptor (DARC).
- Normal serum levels of Glucose-6-phosphate dehydrogenase (G6PDH).
- Negative haemoglobinopathy screen
- Able and willing (in the Investigator's opinion) to comply with all study requirements.
- Willing to allow the Investigators to discuss the volunteer's medical history with their General Practitioner.
- Volunteers with the potential to become pregnant only: Must practice continuous effective contraception* for the duration of the clinic visits (first 3 months post-CHMI).
- Agreement to permanently refrain from blood donation
- Written informed consent to participate in the trial.
- Reachable (24/7) by mobile phone during the period between CHMI and completion of all antimalarial treatment.
- Willing to take a curative anti-malarial regimen following CHMI.
- Willing to reside in Oxford for the duration of the study, until antimalarials have been completed.
- Answer all questions on the informed consent quiz correctly.

* Female volunteers are required to use an effective form of contraception during the course of the study as malaria challenge could pose a serious risk to both maternal health and the unborn foetus.

Acceptable forms of contraception for female volunteers include:

- Established use of oral, injected or implanted hormonal methods of contraception (an additional form of contraception will be required when taking the antimalarial medication, as this can interfere with the efficacy of hormonal contraception).
- Placement of an intrauterine device (IUD) or intrauterine system (IUS).
- Total abdominal hysterectomy.

- Barrier methods of contraception (condom or occlusive cap with spermicide).
- Male sterilisation, if the vasectomised partner is the sole partner for the subject.
- True abstinence, when this is in line with the preferred and usual lifestyle of the subject (periodic abstinence and withdrawal are not acceptable methods of contraception).

7.2.2 Exclusion criteria

The volunteer may not enter the study if any of the following apply:

- History of clinical malaria (any species).
- Travel to a clearly malaria endemic locality during the study period or within the preceding six months.
- Use of systemic antibiotics with known antimalarial activity within 30 days of CHMI (e.g. trimethoprim-sulfamethoxazole, doxycycline, tetracycline, clindamycin, erythromycin, fluoroquinolones and azithromycin).
- Haemoglobin <120 g/L for a female volunteer or <130 g/L for a male volunteer prior to primary CHMI. (However, for enrolment into secondary and tertiary CHMIs slightly lower haemoglobin values (≤0.5 g/L) will be permitted at the discretion of the Investigator, to account for the blood volume donated during the previous CHMI).
- Receipt of immunoglobulins within the three months prior to enrolment.
- Receipt of blood transfusion at any time in the past.
- Peripheral venous access unlikely to allow twice daily blood testing (as determined by the Investigator).
- Receipt of an investigational product in the 30 days preceding enrolment, or planned receipt during the study period.
- Prior receipt of an investigational vaccine likely to impact on interpretation of the trial data or the *P. vivax* parasite as assessed by the Investigator.
- Planned receipt of a COVID-19 vaccine between 2 weeks before the day of CHMI until completion of antimalarial treatment
- Any confirmed or suspected immunosuppressive or immunodeficient state, including HIV infection; asplenia; recurrent, severe infections and chronic (more than 14 days) immunosuppressant medication within the past 6 months (inhaled and topical steroids are allowed).
- History of allergic disease or reactions likely to be exacerbated by malaria infection.
- Pregnancy, lactation or intention to become pregnant during the study.
- Use of medications known to cause prolongation of the QT interval ***and*** existing contraindication to the use of Malarone.
- Use of medications known to have a potentially clinically significant interaction with Riamet ***and*** Malarone.
- Any clinical condition known to prolong the QT interval.
- History of cardiac arrhythmia, including clinically relevant bradycardia.
- Disturbances of electrolyte balance, e.g. hypokalaemia or hypomagnesaemia.
- Family history of congenital QT prolongation or sudden death.
- Contraindications to the use of both of the proposed anti-malarial medications Riamet and Malarone.
- History of cancer (except basal cell carcinoma of the skin and cervical carcinoma in situ).
- History of serious psychiatric condition that may affect participation in the study.
- Any other serious chronic illness requiring hospital specialist supervision.

- Suspected or known current alcohol abuse as defined by an alcohol intake of greater than 25 standard UK units every week.
- Suspected or known injecting drug abuse in the 5 years preceding enrolment.
- Hepatitis B surface antigen (HBsAg) detected in serum.
- Seropositive for hepatitis C virus (antibodies to HCV) at screening or at C-7 (***unless*** has taken part in a prior hepatitis C vaccine study with confirmed negative HCV antibodies prior to participation in that study, and negative HCV RNA PCR at screening for this study).
- Positive family history in both 1st AND 2nd degree relatives < 50 years old for cardiac disease.
- Volunteers unable to be closely followed for social, geographic or psychological reasons.
- Any clinically significant abnormal finding on biochemistry or haematology blood tests, urinalysis or clinical examination. In the event of abnormal test results, confirmatory repeat tests will be requested. Procedures for identifying laboratory values meeting exclusion criteria are shown in Appendix A.
- Any other significant disease, disorder, or finding which may significantly increase the risk to the volunteer because of participation in the study, affect the ability of the volunteer to participate in the study or impair interpretation of the study data.
- Inability of the study team to contact the volunteer's GP to confirm medical history and safety to participate.

Additional exclusion criteria for Groups 6-13

- Body weight <50Kg, as measured at screening

7.2.3 Prevention of 'Over Volunteering'

Volunteers will be excluded from the study if they are concurrently involved in another trial. In order to check this, volunteers will be asked to provide their National Insurance or Passport number (if they are not entitled to a NI number) and will be registered on a national database of participants in clinical trials (***www.tops.org.uk***).

7.2.4 Exclusion criteria on day of CHMI

The following constitute absolute contraindications to CHMI:

- Acute disease, defined as moderate or severe illness with or without fever.
- Current COVID-19 infection, defined as ongoing symptoms with positive COVID-19 PCR swab test taken during current illness or positive COVID-19 PCR swab test within preceding 7 days without symptoms.
- Pregnancy.

Potential volunteers who are due to have a COVID-19 vaccine in the period between 2 weeks before the day of malaria challenge and expected time of completion of malaria treatment will not be enrolled (estimate up to 3 weeks after day of challenge based on experience in this study to date). If a volunteer receives an appointment for COVID-19 vaccination after they have undergone malaria challenge but before reaching malaria diagnosis criteria, they will be advised to delay their COVID-19 vaccination until after reaching malaria diagnostic criteria and completion of antimalarial treatment. If a participant does not wish to delay their COVID-19 vaccination, they will be treated with antimalarial treatment at that time point and withdrawn from the study but will be encouraged to complete follow-up for safety.

7.3 Withdrawal of volunteers

In accordance with the principles of the most recent revision of the Declaration of Helsinki (2013) and any other applicable regulations, a volunteer has the right to withdraw from the study at any time and for any reason, and is not obliged to give his or her reasons for doing so. In addition the volunteer may withdraw/be withdrawn from further study procedures at any time in the interests of the volunteer's health and well-being, or for any of the following reasons:

- Administrative decision by the Investigator.
- Ineligibility (either arising during the study or retrospectively, having been overlooked at screening).
- Significant protocol deviation.
- Volunteer non-compliance with study requirements.
- Incorrect answers to screening questionnaire at the second attempt upon re-screening for either second or third CHMI
- An AE, which requires discontinuation of the study involvement or results in inability to continue to comply with study procedures.

The Local Safety Monitor (LSM) may recommend withdrawal of volunteers. The reason for withdrawal from further study procedures will be recorded in the CRF. If a volunteer withdraws after having completed a course of antimalarials, as much continued safety data collection as possible, including procedures such as safety bloods, will be continued, with agreement of the volunteer. For all AEs, appropriate follow-up visits or medical care will be arranged, with the agreement of the volunteer, until the AE has resolved, stabilised or a non-trial related causality has been assigned.

Any volunteer who fails to attend for two or more follow-up visits will be deemed to have withdrawn from the study. If a volunteer withdraws from the study after CHMI but before reaching the criterion for malaria diagnosis, a complete, appropriate, curative course of anti-malarial therapy must be completed. The importance of this will be emphasised to volunteers at screening. If a volunteer refuses to take anti-malarial therapy after malaria diagnosis, a rapid assessment of mental state and capacity will be undertaken, with the involvement of NHS psychiatric and infectious diseases services. If necessary the volunteer may be detained under section 4 of the UK Mental Health Act until this assessment can be carried out.

If a volunteer withdraws from the study, blood samples collected before their withdrawal from the trial will be used/stored unless the volunteer specifically requests otherwise. Similarly, all data collected up to the point of withdrawal will be stored, unless they specifically request for it to be destroyed. Volunteers are free to request that their blood samples be destroyed anytime during or after the study.

In all cases of subject withdrawal, excepting those of complete consent withdrawal, safety data collection will continue until 96 days after challenge (C+96), if subjects have undergone CHMI.

7.4 Pregnancy

Should a volunteer become pregnant during the trial, she will be treated with antimalarials immediately and will be withdrawn from the study. We will not routinely perform venepuncture on such volunteers, other than blood films to check that the parasitaemia has been cleared by the antimalarials. Additionally, if a volunteer who had been negative for CMV IgG antibodies at screening should become pregnant during the study, given the theoretical risk of CMV infection from exposure to the inoculum, venepuncture would also be performed to check CMV serostatus. With the volunteer's permission she shall be followed up until pregnancy outcome.

The management of any volunteer found to be pregnant at any time after challenge up to the point of malaria treatment will be discussed with the on-call infectious diseases consultant at the Oxford University Hospitals NHS Foundation Trust, including advice on antimalarial drug choice.

Should a volunteer become pregnant after receiving antimalarial treatment (but prior to the end of the study), they shall be withdrawn from the study as soon as we have confirmed that their parasitaemia has cleared. With the volunteer's permission, she shall then be followed up until pregnancy outcome.

8 CONTROLLED BLOOD-STAGE MALARIA INFECTION INOCULUM

8.1 Preparation of the inoculum

Thawing and washing of the inoculum will be done with commercial solutions for human use and with disposable syringes and needles according to standard operating procedures used in previous studies at Oxford [57, 58]. Work will be carried out in the derogated category III laboratory at the Jenner Institute, Old Road Campus Research Building (ORCRB) or New Biochemistry Building. Sample manipulations will be performed within a safety cabinet that has been fumigated, sterilised and dedicated for this purpose.

Thawing of the blood inoculum and preparation of the syringes will be carried out in accordance with local standard operating procedures.

8.2 Administration of the inoculum

The inoculation will take place in Oxford at the CCVTM. The inoculum will be administered by intravenous injection into an indwelling intravenous cannula.

In VAC069A, the first *P. vivax* CHMI, either one whole vial, containing approximately 0.5mL of red blood cells, one fifth of a vial, or one twentieth of a vial will be reconstituted in 0.9% saline, to a total volume of 5mL. The reconstituted inoculum will be injected via an indwelling cannula, preceded and followed by a saline flush. One volunteer from each group (Groups 1-3), receiving one of the three concentrations, will be administered the inoculum first. The inoculum will subsequently be administered to each of the second three volunteers from each group. This is in case the time between administration of the inoculum affects the viability of the parasites.

In subsequent phases of the study using *P. vivax* VAC069B to D, the inoculum dose will be the same for all volunteers and based on the optimal dose as determined in VAC069A. Results from VAC069A showed that all volunteers were successfully infected, with a mean day of diagnosis of 15 days after CHMI (range 12.5-16.5). The two volunteers receiving the highest inoculum dose were diagnosed at days 12.5 and 15.5 respectively, those receiving a 1:5 dilution were both diagnosed at day 15 and those receiving the 1:20 dilution were diagnosed at days 15.5 and 16.5. All three dilutions were therefore demonstrated to be reliably infective. Based on these infectivity data, and accounting for possible variation in number of parasitised erythrocytes between thawed cryopreserved vials, an inoculum dilution of 1:10 (prepared as per Jenner laboratory SOP) has been chosen for all subsequent CHMI.

In the final phase of the study VAC069E, the *P. falciparum* inoculum 3D7 will be used for CHMI. Each vial of the 3D7 inoculum is estimated to contain between ~3.9×10^6 to 5.2×10^8 erythrocytes, of which ~1 in 40 will be parasite-infected erythrocytes. The inoculum will be diluted with saline to achieve a final dose of approximately 1,000 infected red blood cells in a total volume of 5 mL of normal saline, to be injected intravenously.

For all phases of the study, all volunteers will receive the inoculum within a maximum of 4 hours of removal from frozen storage. Subjects will be observed for a minimum of one hour after the inoculation before being discharged from the clinic.

9 TREATMENT OF TRIAL VOLUNTEERS

This section describes the clinical procedures for evaluating study participants and follow-up after CHMI. All participants (including back-up volunteers) will be required to attend all visits up to the point of malaria challenge. Back-up volunteers also must be available on the morning of the malaria challenge, in case one of the volunteers withdraws at the last minute. This means that confirmation of whether or not a back-up volunteer will be needed for the challenge will not be made until the day of challenge (one of the Investigators will call the back-up volunteer that morning to confirm either way).

9.1 Trial sites

Volunteers will be recruited and undergo screening visits, and clinic visits post-CHMI at the CCVTM, Oxford. From day of challenge until completion of anti-malarial therapy, all volunteers will reside in Oxford or surrounding areas and attend follow-up visits at the CCVTM, Oxford. Post-challenge follow up visits will also take place in Oxford. Mosquito-bite transmission studies will take place at the Old Road Campus Research Building, Oxford.

9.2 Study procedures

Procedures will be performed at the time points indicated in the schedule of procedures (Tables 5-14). Additional procedures or laboratory tests may be performed, at the discretion of the Investigators if clinically necessary (e.g. urine microscopy in the event of positive urinalysis).

Study procedures for the first, second and third challenge will be the same, with the exception of differences at re-screening, as compared to initial screening, prior to the second and third challenges, as described below.

9.2.1 Observations

Pulse, blood pressure and temperature will be measured at the time points indicated in the schedule of procedures. Weight and height will be measure at screening and weight shall be re-measured at C-1 (C-2 for VAC069C onwards).

9.2.2 Blood tests

Blood will be drawn at the time points indicated in the schedule of procedures below and the following laboratory assays performed:

At Oxford University Hospitals NHS Foundation Trust, using NHS standard procedures

- **Haematology;** Full blood count, to include reticulocyte count at C-2 visit. Duffy antigen/chemokine receptor (DARC) gene positivity, and glucose-6-phosphate dehydrogenase (G6PDH) levels and a haemoglobinopathy screen will be performed at screening only. Stored serum or blood will be tested for red blood cell alloantibodies in a sample taken at C-1/C-2 of the volunteer’s 1st CHMI and at C+90/96 following each CHMI.
- **Biochemistry;** Sodium, potassium, urea, creatinine, albumin, liver function tests, magnesium and calcium. Magnesium and calcium will only be measured at screening and re-screening. Serum will also be tested for beta-human chorionic gonadotrophin (β-HCG) at C-1 or C-2, C+7, C+14, C+21 and C+28/day of malaria diagnosis.
- **Diagnostic serology;** HBsAg, HCV antibodies, HIV antibodies, CMV antibodies and EBV antibodies will be performed at screening, re-screening and C+90 or C+96; and HTLV antibodies at screening. If a volunteer is seropositive for EBV or CMV at screening, further serological testing for EBV or CMV will not be conducted post-CHMI.
- **Immunology;** Human Leukocyte Antigen (HLA) typing at C-1 or C-2.

At the University of Oxford research laboratories (at the Jenner Institute and Department of Biochemistry)

- **Diagnostic Tests:** Blood films for malaria parasites and PCR for *P. vivax* and *P. falciparum* DNA and gametocytes.
- **Immunology:** Immunological responses to *P. vivax* and *P. falciparum* infection will be assessed by a variety of established and exploratory assays. This may include:
    - Antibodies and B cell responses to whole parasites or red blood cell invasion ligands by array, ELISA and flow cytometry, including total IgG, isotypes and avidity;
    - T cell responses to parasite antigens by *ex-vivo* ELISpot and flow cytometry assays;
    - High dimensional cell phenotyping by mass cytometry
    - Serum cytokine analysis.
    - B cells, plasma and/or serum may be analysed and used to produce human monoclonal antibodies against *P. vivax* or *P. falciparum* malaria.
    - Neutralising in vitro antibody assays.
- **Genetic tests:** DNA analysis of genetic polymorphisms potentially relevant to immunological responses and gene expression studies of the host and the parasite amongst others may be performed at the discretion of the Investigators.
- **COVID-19 serology:** Antibody to SARS-COV-2 will be measured at screening, re-screening and at the final study visit following the final CHMI for each participant (+/- at interim time points retrospectively)
- Samples may be sent to collaborating laboratories for other immunological assays or studies of malaria.
- Samples may also be sent to collaborating laboratories within and outside the UK for immunomonitoring and/or harmonisation of key immunological assays and/or studies of malaria.

Immunological assays will be conducted according to the procedures established in the test laboratories. Specific details of which immunological tests will be performed at each time-point are detailed in the separate immunomonitoring plan – this will also clearly state which assays are conducted to meet the trial's primary objectives and which are exploratory. With the volunteers' informed consent, any leftover cells, plasma, serum, whole blood (or their purified components) will be registered under University of Oxford HTA licence 12217 and stored indefinitely for future analysis of human malaria infection. This may include human DNA and RNA analysis. If a subject elects not to permit this, all of that subject's leftover samples will be discarded after the required period of storage to meet Good Clinical Practice (GCP) and regulatory requirements.

Depending on observed gametocytaemia and availability of appropriate laboratory–reared *Anopheline* mosquito vectors, blood may also be drawn for Direct Membrane Feeding Assays (DMFA) to assess transmission to mosquitos. This will involve a 10ml blood draw on day 14 post-challenge and/or on day of diagnosis, whichever is sooner.

9.2.3 Urinalysis

Urine will be tested for the presence of clinically significant proteinuria, glycosuria or haematuria (as defined in Appendix A) at the screening and re-screening visits. For female volunteers only, urine will be tested for beta-human chorionic gonadotrophin (β-HCG) at screening, re-screening and at diagnosis, prior to initiation of treatment.

9.2.4 Electrocardiogram

An electrocardiogram will be performed at the initial screening visit for detection of any conduction abnormalities, including long QT syndrome.

9.2.5 SARS-COV-2 testing

Whilst the COVID-19 pandemic is ongoing, a SARS-COV-2 antigen test will be undertaken within 2 days of the day of malaria challenge in all volunteers. This can either be a lateral flow test or a PCR test and can be self-administered. Asymptomatic COVID-19 infection will be a contra-indication to proceeding to CHMI. Any participant who develops symptoms, which may be consistent with COVID-19 disease after undergoing malaria challenge and prior to completing malaria treatment, may also undergo testing for COVID-19 by PCR swab or lateral flow test or asked to self-administer a test at home. If a volunteer tests positive on a lateral flow test, it will be at the Investigator's discretion to perform a confirmatory PCR swab test.

If a participant develops symptoms consistent with COVID-19 after completing malaria treatment, they will be referred to local community testing for COVID-19.

9.3 Clinical reviews

All clinical reviews and procedures will be undertaken by one of the clinical team. The procedures to be included in each visit are documented below. Each review is assigned a time point and a window period within which the review will be conducted.

9.3.1 Screening visits

Where possible, and where agreement to contact by telephone has been provided by email, individuals may be phoned by a member of the clinical team to discuss the study, prior to the screening visit. This pre-screening telephone call will provide an opportunity for interested individuals to discuss the study, eligibility criteria and study requirements. Where a pre-screening call takes place, if individuals continue to express an interest in taking part in the trial following the pre-screening call, a screening visit will be scheduled.

Screening visits may take place up to 90 days prior to enrolment. Informed consent will be gained at screening. If consent is given, the screening procedures indicated in the schedule of procedures (Table 5) will be undertaken, including testing for Duffy antigen/chemokine receptor (DARC) positivity, G6PDH levels and haemoglobinopathy screen.

The subject's general practitioner will be contacted with the written permission of the subject after screening to ascertain any significant medical history and as notification that the subject has volunteered for the study. During the screening the volunteers will be asked to provide their National Insurance or passport number so that this can be entered on to a national database which helps prevent volunteers from participating in more than one clinical trial simultaneously or over-volunteering for clinical trials (TOPS).

Abnormal clinical findings from the medical history, physical examination, urine or blood tests at any point in the study will be assessed according to the scales in Table 15 and site specific laboratory adverse event grading tables kept in the trial master file (ref: SOP VC027). If a test is deemed clinically significant it may be repeated to ensure it is not a single occurrence. If an abnormal finding is deemed to be clinically significant, the volunteer will be informed and appropriate medical care arranged with the permission of the volunteer. Exclusion of the

volunteer from enrolling in the trial or withdrawal of a volunteer from the trial will be at the discretion of the Investigator.

9.3.2 Re-screening visits for participants not yet enrolled in the study

Where more than 90 days has lapsed between the initial screening visit and date of enrolment, a re-screening visit will be conducted to ascertain if there have been any changes in the medical history, medical examination findings or urinalysis, or blood tests for; haematology, biochemistry, liver function tests or blood borne virus serology (HIV, Hepatitis B, Hepatitis C, EBV and CMV). Other blood tests and ECG will not be repeated routinely. In addition, where a reply from the subject's GP has already been received, the GP will not be re-contacted unless there is any need to obtain further information where appropriate (e.g. regarding a new medical event or finding) and at the discretion of the Investigator.

9.3.3 Re-screening visits prior to 2nd and 3rd Challenges

A re-screening visit will take place prior to each subsequent challenge. Re-screening visits may take place up to one month and no less than 1 week prior to re-enrolment. Re-screening visits prior to the *P. falciparum* CHMI in VAC069E may take place up to 90 days and no less than 1 week prior to re-enrolment. This will be to identify any new medical events. A new informed consent will be obtained at the re-screening visit. If consent is given, study specific procedures at the visit will be the same as for the screening visit, with the exception that testing for Duffy antigen/chemokine receptor (DARC) positivity, G6PDH levels or haemoglobinopathy screen will not be repeated (as described in Table 5). In addition, the volunteer's general practitioner will not be re-contacted to corroborate their medical history, unless the Investigators have any concerns, e.g., if any new medical events are declared. However, the general practitioner will be informed of the volunteer's intention to participate in subsequent challenges.

9.3.3 Two days before CHMI (C-2)

Any new medical issues or symptoms that have arisen will be assessed. Physical observations, serum β-HCG test in female volunteers and venepuncture for immunology, malaria PCR and safety bloods will be undertaken according to Table 5. A SARS-COV-2 test will also be taken either by the study team or self-administered by the participant. Results of safety bloods and the SARS-COV-2 test taken for this visit must be available and reviewed prior to challenge. Any participant with a positive SARS-COV-2 test will not proceed to CHMI.

9.3.4 Day of CHMI (C0)

CHMI will be administered, according local standard operating procedure (SOP), this will include

- Interim history and examination of the injection site and any body systems felt to be necessary by the Investigator and verify continuing eligibility / contraindications.
- Baseline physical observations (respiratory rate, pulse, blood pressure and temperature).
- Intravenous access via cannulation in forearm vein, flushed with normal saline.
- For each volunteer the inoculum must be injected within 4 hours of the inoculum being thawed, preceded and followed by further normal saline flushes.
- Physical observations will be repeated at 15 minutes (+/- 5 mins) post-administration of inoculum and again at 1 hour (+/- 20 mins), in order to assess for immediate adverse reactions, with regular visual observation throughout [76]. If the volunteer should show signs or symptoms of a transfusion reaction, observations will be repeated and the volunteer will be medically reviewed by a physician.

- Volunteers will be observed for at least 60 minutes, however, this period may be extended if there are any clinical concerns.
- If there have been no symptoms or signs indicative of a transfusion reaction, the cannula will be removed after 1 hour.

Before leaving CCVTM, volunteers will be informed about the unlikely possibility of a delayed transfusion reaction and will be provided with the 24 hour emergency mobile telephone number, to enable them to contact a physician in the event of concern.

A Medic-Alert type card will be issued to each volunteer. This will contain the study physician 1contact details and a request that the research team be contacted immediately in the event of illness/accident. Each subject will also be issued with an accurate oral thermometer. If the subject does not have their own mobile telephone they will be issued with one for the duration of the study, and counselled about the importance of keeping it switched on or checking the messages regularly. In addition full contact details for each subject will be documented, including home and work addresses, home and work landline telephone numbers where available and next-of-kin address and telephone numbers. Mobile telephone numbers will be verified prior to challenge to ensure the volunteers are easily contactable. Subjects must also provide the Investigators with the name and 24 hour telephone number of a close friend, relative or housemate who will be kept informed of their whereabouts for the duration of the study.

Volunteers must be resident in Oxford / the surrounding area until they have two consecutive qPCR results with substantial reduction in genome copies/mL after commencing antimalarial treatment. If volunteers do not reach malaria diagnosis, they must remain in the Oxford area until they have completed anti-malarial treatment.

Volunteers will be counselled that should they fail to return for treatment having been infected with *P. vivax* or *P. falciparum* malaria they could become very unwell and potentially die. They will be informed that should they fail to attend a scheduled clinic visit post-challenge, their nominated contact, next of kin and the police may be informed and a search started.

9.3.5 Days 1-28 post-CHMI (C+1 – C+28)

VAC069A

Volunteers will be reviewed in clinic once in the morning of day 1 after challenge (C+1), then twice daily from day 2 until day 12 post-challenge inclusive (C+2-C-12.5). From day 13 to day 20 (C+13-20.5), visits may either be once or twice daily depending on the qPCR result. Once a qPCR threshold of 1000 genome copies/µL is reached, visits will continue twice daily. If qPCR has not reached this threshold, visits will reduce to once daily only.

VAC069B

Volunteers will be contacted by phone daily from the day following challenge (C+1) until day 5 post–challenge (C+5). From days 6-9 (C+6 to C+9) they will attend clinic once daily for medical review and assessment of parasitaemia, by both qPCR and smear microscopy. From day 10 onwards they will attend clinic twice daily, in the early morning and late afternoon, for medical review and monitoring of parasitaemia, by qPCR and smear microscopy.

From day 21 after challenge, visits may be reduced to once daily at the discretion of the Investigator (as agreed by the Chief Investigator) e.g., if qPCR is very low or undetectable, and volunteer is completely asymptomatic (qPCR results may be unblinded for this purpose).

VAC069C

Volunteers will be contacted by phone daily from the day following challenge (C+1) until day 6 post–challenge (C+6). From days 7-9 (C+7 to C+9) they will attend clinic once daily for medical review and assessment of parasitaemia. From day 10 onwards they will attend clinic twice daily, in the early morning and late afternoon, for medical review and monitoring of parasitaemia by qPCR.

From day 21 after challenge, visits may be reduced to once daily at the discretion of the Investigator (as agreed by the Chief Investigator) e.g., if qPCR is very low or undetectable, and volunteer is completely asymptomatic (qPCR results may be unblinded for this purpose).

VAC069D

Following review of the results from the CHMI in VAC069C and the VAC079 vaccine and challenge trial running in parallel, the number of visits in the post challenge period has been reduced for subsequent challenges to minimise the number of bleeds and burden on volunteers.

Review of the qPCR results and parasite multiplication rate (PMR) modelling from the CHMI in VAC069C and VAC079 has shown that sufficient data can be obtained for accurate PMR modelling if bleeds for qPCR continue once a day until qPCR reaches ≥1000 genome copies/ml, instead of starting twice a day visits from C+10 onwards. Once qPCR levels reach ≥1000 genome copies/ml, then visits will increase to twice a day in order to not delay diagnosis as qPCR may reach the diagnostic threshold of ≥5000 genome copies/ml with malaria symptoms within about half a day. In volunteers in the VAC079 study, who were diagnosed between days 19 to 27 post challenge, PMR modelling would also be minimally affected if participants are treated at C+21, instead of C+28, even if they have not reached malaria diagnostic criteria at that timepoint. Changing both parameters, shortening time to treatment from C+28 to C+21 and reducing frequency of bleeds from twice to once daily until >1000 genome copies/mL threshold is reached, did not significantly change the PMRs and also did not change the significance of the comparison between the vaccine and the control groups. In VAC069C, volunteers reached diagnostic criteria between C+14 to C+19 with median of 15 days post challenge, so the changes will have less impact on the number of visits in control volunteers in VAC069. However the post-challenge visits will be standardized between the two vaccine trials (VAC071 and VAC079) and VAC069 to allow direct comparison between controls and vaccinees.

Volunteers in phase VAC069D to F will therefore be contacted by phone daily from the day following challenge (C+1) until day 6 post-challenge (C+6). From days 7 to 9 (C+7 to C+9) they will attend clinic once daily for medical review and assessment of parasitaemia. From day 10 onwards they will attend visits daily, until their malaria qPCR count reaches ≥1000 genome copies/ml, when visits will increase to twice a day. Visits will continue twice a day until diagnosis, or if diagnosis criteria are not met, until day 21 post-challenge inclusive (C+21).

VAC069E

Volunteers will be contacted by phone daily from the day following challenge (C+1) until day 4 post-challenge (C+4). On days 5 to 6 (C+5 to C+6) they will attend clinic once daily for medical review and assessment of parasitaemia. From day 7 onwards volunteers will attend visits daily, until their malaria qPCR count reaches ≥1000 genome copies/ml, when visits will increase to twice a day until diagnosis. If diagnosis criteria are not met, visits will continue, once or twice daily depending on the qPCR count, until day 21 post-challenge inclusive (C+21).

All volunteers will be seen by one of the clinical study team at each visit. The team will consist of the Investigators who are physicians (with experience in acute medicine and infectious diseases and familiarity assessing patients with malaria) and nurses.

At each follow-up visit:

- Physical observations will be performed. Venepuncture will be performed as per schedule of attendance (Table 6-10), including blood for assessing parasite growth rate, immune response and level of gametocytaemia.
- Volunteers will be questioned as to whether they have;
    - Experienced any of the foreseeable symptoms of malaria.
    - Experienced any other symptoms.
    - Taken any medications including over the counter medications.

Full physical examination will be performed if deemed necessary by the Investigators. Subjects will be encouraged to contact one of the Investigators on the 24 hour emergency mobile telephone number if they develop symptoms of malaria or concerning AEs between the regular clinic reviews. The severity of symptoms will be assessed using grading criteria summarised in Tables 12 & 13.

Diagnosis will be made according to criteria described below. In VAC069A, if volunteers reach day 21 post-challenge (C+21) without reaching diagnostic criteria, they shall be started on antimalarials. In VAC069B and C, if diagnosis criteria have not yet been met, antimalarial treatment shall be initiated at day 28 after challenge (C+28). For VAC069D to E, if diagnosis criteria have not yet been met, antimalarial treatment shall be initiated at C+21. Subjects will be reviewed in clinic in the subsequent days after starting anti-malarial therapy, as described in section 9.5.

If a volunteer is unwell and unable to attend the CCVTM for a visit they will be reviewed on the phone and appropriate arrangement made for clinical review as appropriate.

9.3.6 Direct feeding of mosquitos

Direct feeding of mosquitos on the volunteers' skin may also be performed if the volunteers additionally consent to this. This is to assess whether the sexual form of the parasite (the gametocyte) can be transmitted back to, and develop within, the mosquito host. This will take place at the Old Road Campus Research Building (ORCRB), just a short walk from the CCVTM. The procedure will involve laboratory-reared *Anopheline* mosquitos in a gauze-covered paper cup being placed on the volunteer's exposed skin and allowed to bite. As the biting behaviour of the mosquitos is variable, this procedure may take up to a few hours and volunteers will be compensated accordingly. Volunteers will be offered a topical antihistamine to reduce itching post-mosquito bite.

9.4 Algorithm for initiation of treatment

9.4.1 Algorithm for initiation of treatment in Groups VAC069A&B

Diagnostic criteria will be based on symptoms and signs in-keeping with malarial infection, thick film microscopy results and real-time quantitative polymerase chain reaction (qPCR), summarised in the algorithm in Figure 2.

Symptoms or signs that will be considered as indicative of symptomatic malarial infection will be fever >37.8^{o}C, rigors or other severe symptoms or signs related to malarial infection, including, but not limited to, sweats, headache, anorexia, nausea or vomiting.

Microscopy will be performed by one or more experienced microscopists and a positive microscopy result will be defined as the detection of at least two morphologically normal malaria parasites seen in 200 high-power (1000x) fields. This will be diagnostic in the presence of symptoms in-keeping with malaria infection. If the volunteer is asymptomatic, diagnosis will be

based on a combination of microscopy and qPCR results, as detailed below. Further microscopy, e.g. thin film microscopy, may also be performed in order to obtain further information on parasite life-cycle stage, but these results will not be considered for diagnosis and treatment decisions.

Asexual blood-stage parasitaemia will be measured by qPCR for the *P. vivax* 18S rRNA gene. In the presence of symptoms, >5,000 genome copies/mL will be considered diagnostic but a qPCR result >10,000 copies/mL will be considered positive, even in the absence of symptoms.

The Investigators are able to treat any volunteer for malaria regardless of the thick film microscopy or qPCR result if they are clinically concerned (and have discussed the case with the Chief Investigator), or a volunteer wishes to withdraw from the study. If necessary, volunteers can be discussed with the LSM and/or the infectious diseases consultant on call at the John Radcliffe Hospital for further management under the care of the infectious diseases team, inclusive of parenteral anti-malarial therapy if deemed appropriate.

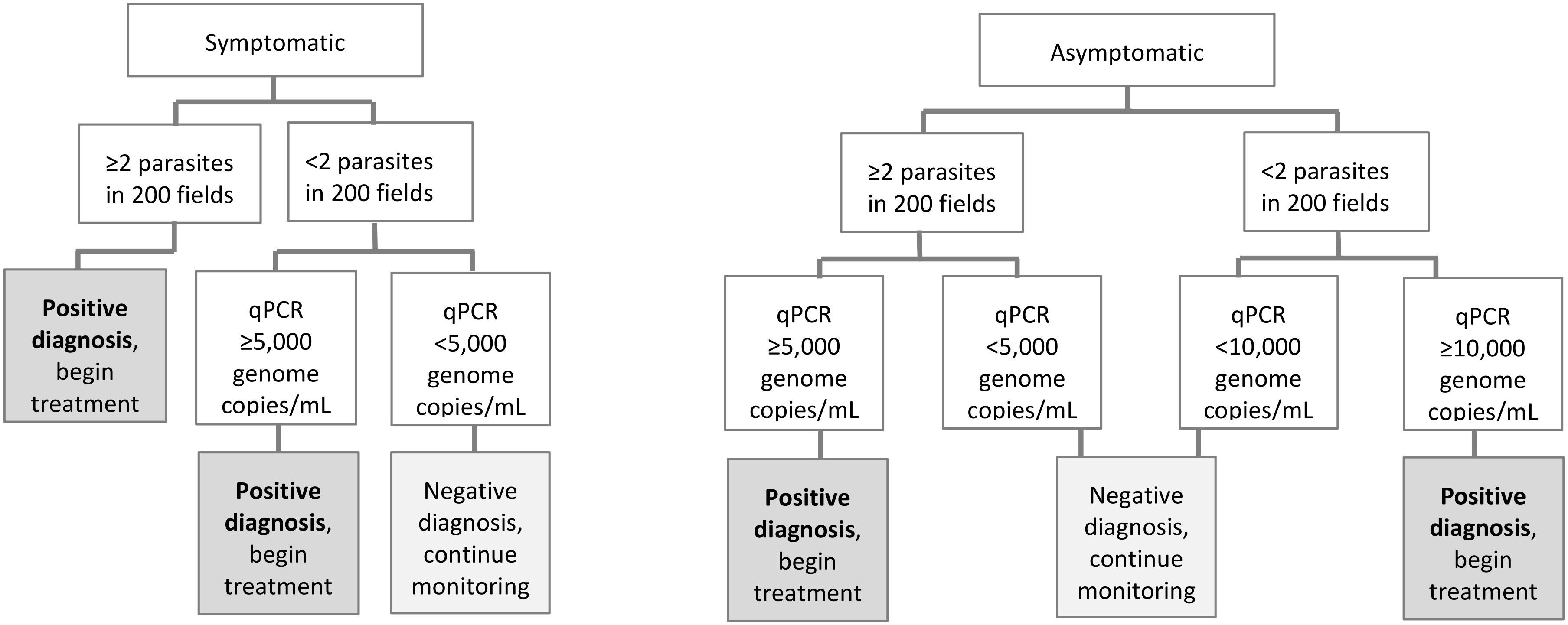

Figure 2: Algorithm for diagnosis and initiation of treatment VAC069A&B.

9.4.2 Algorithm for initiation of treatment in Groups VAC069C-E

It is now well established that nucleic acid amplification-based diagnostics have greater sensitivity than microscopic methods for detecting malaria infection both in clinical infection and in the *P. falciparum* challenge model. In the latter, qPCR targeting the 18S ribosome DNA target has been used as the diagnostic tool, reliably detecting parasitaemia earlier than thick film blood smear[77-80].

The qPCR assay that we have used in numerous Oxford-led CHMI *P. falciparum* challenge studies is now qualified and highly sensitive, reliably detecting parasitaemias as low as 20 parasites per mL of blood (0.02 parasites per µL), long before clinical symptoms manifest. The qPCR assay has also performed well in an international External Quality Assurance (EQA) exercise [77].

It is also important to note that other trial centres in the USA routinely use PCR as a diagnostic tool in CHMI studies. NCT020-15091 was a multi-institution, Phase I, open-label, dose-escalation trial with CHMI, designed to assess the safety, immunogenicity, and protective efficacy of PfSPZ Vaccine, and this used PCR as the primary diagnostic [81].

For both *P. vivax* and *P. falciparum*, we use a qPCR assay targeting the pan-plasmodium 18S ribosome DNA target. The only other centre worldwide that routinely performs blood-stage challenge studies for *P. vivax* (QIMR, Australia), uses PCR as the sole diagnostic tool in CHMI studies, and our qPCR assay is based on this, utilising identical primer and probe sequences. In all other aspects, apart from the QIMR derived detection sequences and reaction mixture employed, the extraction of DNA and qPCR is identical to the existing and formally validated *P.falciparum* qPCR method employed as sole, objective, diagnostic tool in the two *P.falciparum* trials described below. This pan-plasmodium qPCR is in the process of undergoing a similar formal validation and work completed so far, demonstrates that this is similarly fit for purpose. As examples of qualification parameters that contribute to validation, the assay has excellent specificity (within the context of a *P.viax* only CHMI study), linearity, acceptable accuracy (based on reference samples of microscopically quantified *P.vivax* infected blood), precision and reproducibility (based on several inter-operator tests with acceptable %CV values when assessing prepared samples at the described diagnostic thresholds in >20 replicates). The assay has an acceptable detection and reliable quantitation limit of 50-100 genomes per ml (well below reproducible microscopic detection levels. While detection is possible down to approximately 25 genomes per ml as indicated below, it is less reproducible at this level). The assay is also extremely robust, with both EDTA whole blood samples, extracted DNA and standards remaining stable and giving reproducible qPCR scores at 4°C storage for many days (which is beyond the intended use for real time qPCR follow up). The range of the qPCR is similar to that of the previously validated *P.falciparum* assay, with at least a 4 log range from 100 genomes per ml to 1,000,000 for the standards employed (actual range used in assays is from 25 to 1,000,000 genome copies per ml). These criteria assessments are in accordance with the ICH Harmonised Tripartite Guideline Part II: Validation of Analytical Procedures: Methodology, (European Medicines Agency, 2006). This is further supported by data from the lab's participation in the UKNEQAS external quality assurance scheme since mid-2018, with 5 rounds of samples assayed in both *P.falciparum* and *P.vivax* assays completed to date, with a 100% success rate.

To date, comparative qPCR and microscopy data are available for two blood-stage *P. vivax* CHMI studies conducted with the PvW1 blood inoculum in Oxford; VAC069A&B (n=11 including 3 participants who underwent CHMI twice) (NCT03797989) and CHMI of VAC071 Group 1 participants (n=3) (NCT04009096). Here, the greater sensitivity of qPCR compared to thick film microscopy has been consistently demonstrated with 3/14 participants diagnosed by qPCR prior to microscopic patency and, where infection was microscopically patent (11/14 participants),

qPCR detected parasitaemia (>25 genome copies/mL) at least 6 days prior to thick film positivity (as defined as ≥2 asexual forms seen in 200 high-power fields). At the pre-specified qPCR thresholds for diagnosis, as described in Figure 2, 3/14 participants did have a positive thick film result prior to reaching diagnostic criteria (≥5,000 genome copies/mL), however, none of these three participants had experienced symptoms consistent with the clinical threshold for diagnosis at the time of thick smear positivity and were later diagnosed within 1.5 days of microscopic patency, without complication.

Positive thick smear results may result from chance findings, since following blood-stage challenge, parasites may be seen on light microscopy at any time-point. Where this occurs, and premature diagnosis results, this limits the qPCR dataset available for PMR modelling. Removing this possibility, therefore, increases the data available for analysis of the primary outcome measure. In addition, use of qPCR as the sole diagnostic tool leads to greater standardisation of diagnosis across study groups, permitting improved inter-group as well as inter-trial comparison. In Oxford, we have now safely conducted two *P. falciparum* blood-stage CHMI studies, where qPCR is the sole diagnostic method (Clinicaltrials.gov identifiers NCT03906474 and NCT02927145). Analysis of the parasitaemia at diagnosis in one of these studies, VAC063B, indicates that removal of microscopy as a diagnostic measure, reduced the range over which diagnosis was made compared to two prior *P. falciparum* blood-stage CHMI studies, where microscopy was utilised (see Figure 3).

As microscopy findings are not considered within the primary efficacy analysis for this study, microscopy for Group 1 participants was principally utilised as an additional diagnostic tool for safety reasons. The data from the two previous *P. vivax* blood-stage CHMI studies were therefore analysed to apply the revised diagnostic criteria retrospectively, to determine if timing of diagnosis would have been altered. Using the revised criteria, the majority of diagnoses would not have been delayed, although it is estimated 36% of volunteers would have had a small delay to diagnosis. However, since clinical criteria remain integral to the diagnostic algorithm, this small delay should not result in any increased risk to volunteers, and the ability for investigators to treat any participant in the event of clinical concern, following discussion with the CI, regardless of parasitaemia, will ensure participant safety.

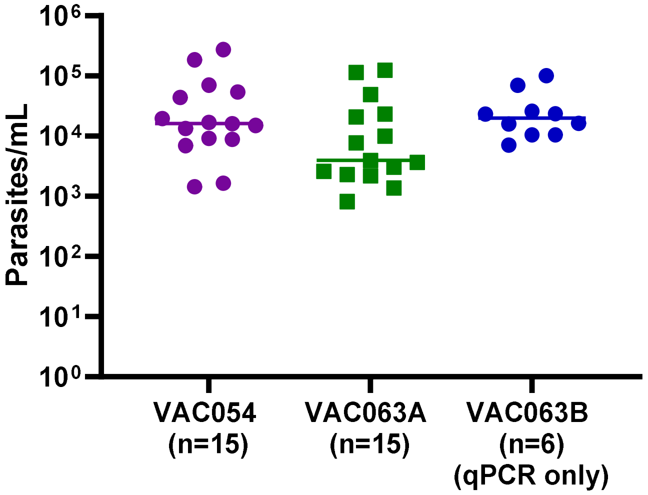

Figure 3: Parasitaemia at the diagnosis timepoint in unvaccinated control volunteers in three prior P. falciparum blood-stage CHMI studies. *In both the VAC054 and VAC063 CHMI studies, where microscopy was used as a diagnostic measure alongside qPCR and clinical assessment, diagnosis was made over a wider range of parasitaemia compared to the VAC063B study, where thick blood smear microscopy was not performed. Diagnostic parasitaemia ranged between 1440-273,247 parsites/mL in the VAC054 study, 815- 124063 parasites/mL in VAC063A and 7137-100946 parasites/mL in the VAC063B CHMI. Individual and median data points are shown[22].*

On this basis, for VAC069C-E, diagnostic criteria will be based on symptoms in keeping with malarial infection results and real-time quantitative polymerase chain reaction (qPCR) only, i.e. thick film microscopy will not be used as a diagnostic measure, as summarised in the algorithm in Figure 4. Symptoms or signs that will be considered as indicative of symptomatic malarial infection will be fever >37.8^{o}C, rigors or other severe symptoms or signs related to malarial infection, including, but not limited to, subjective feverishness, sweats, headache, myalgia, arthralgia, nausea or vomiting.

Asexual blood-stage parasitaemia will be measured by qPCR for the pan-plasmodium 18S DNA gene. In the presence of symptoms or signs indicative of malarial infection, ≥5,000 genome copies/mL will be considered diagnostic but a PCR result ≥10,000 copies/mL will be considered positive, regardless of symptoms. All Investigators (except the Principal Laboratory Investigator) will be blinded to the results. Blinding of PCR operators to the group of a volunteer will be maintained by assigning a new, unique identifier to the volunteer for PCR samples from the C-1 visit. If, as judged by the investigator, a volunteer develops symptoms in-keeping with malaria, the volunteer will be discussed with the Chief Investigator. If deemed appropriate by the Chief Investigator, the qPCR results for this volunteer will be unblinded by the Principal Laboratory Investigator and the volunteer treated only if any available qPCR result is ≥5000 genome copies/mL. At the point that a volunteer has a qPCR result exceeding 10,000 genome copies/mL, the Principal Laboratory Investigator will unblind the Chief Investigator to the result and the volunteer will be treated, regardless of the presence of symptoms.

Importantly, in the unlikely event of a technical failure with the qPCR machine, we also have a working back-up qPCR machine that will be available for use 24/7 if required.

The Investigators are able to treat any volunteer for malaria regardless of the qPCR result if they are clinically concerned (and have discussed the case with the Chief Investigator), or a volunteer wishes to withdraw from the study. If necessary, volunteers can be discussed with the LSM and/or the Infectious Diseases Consultant on call at the John Radcliffe Hospital for further management under the care of the infectious diseases team, inclusive of parenteral anti-malarial therapy if deemed appropriate.

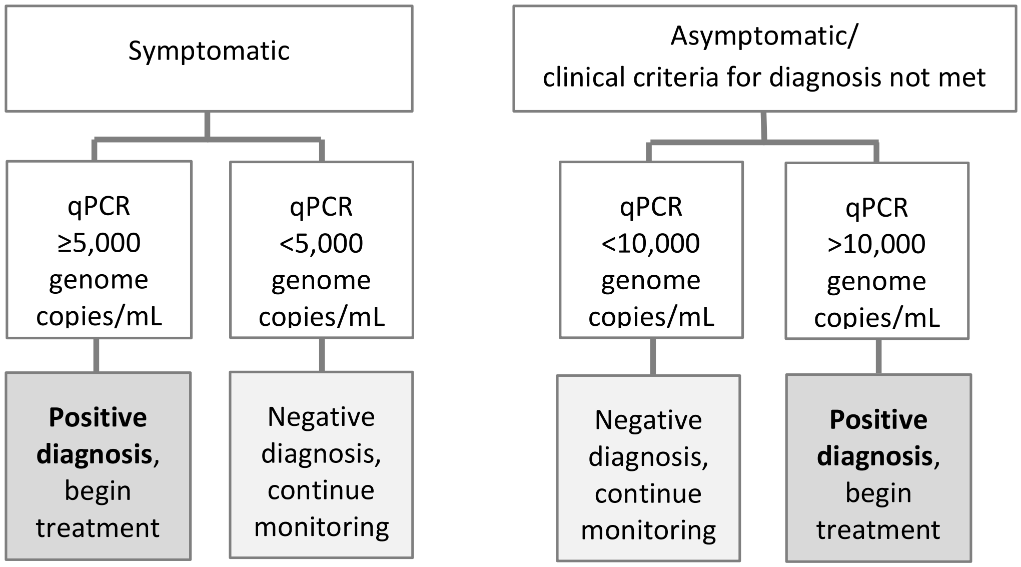

Figure 4: *Algorithm for diagnosis and initiation of treatment in VAC069C-E.* *N.B. In VAC069C, if day 28 after challenge is reached without meeting any diagnostic criteria; or day 21 in VAC069D and E, treatment will be initiated regardless of qPCR results, i.e. even if parasitaemia is undetectable.*

9.4.3 Participants with fever post malaria challenge and COVID-19 testing

In the first week following CHMI, if a participant becomes unwell with a febrile illness, it is highly unlikely that the cause of this is malaria. If a participant develops a fever between days C+1 to C+6 and their symptoms are consistent with possible COVID-19 disease the participant will be advised to follow UKHSA guidelines regarding staying at home and will be advised to take a COVID-19 test with the kit provided by the study team. If the SARS-COV-2 PCR test is negative, the participant can continue with study visits as per protocol.

From C+7 onwards, if a participant develops a fever of ≥37.8°C or other symptoms consistent with possible COVID-19 disease, they will be advised to inform the study team before attending their clinic visit. The participant's clinic visit will be conducted in an isolated clinic room with study staff wearing appropriate PPE. If a participant fulfils current COVID-19 testing criteria, the study team will either advise the participant to perform a home COVID-19 rapid antigen test (provided) or a COVID-19 test will be taken by the study team in addition to the study specified malaria blood tests.

If a participant has a persistent fever or study clinicians are clinically concerned for COVID-19 disease, then a SARS-COV-2 test can be repeated at the discretion of the Investigator, if the first test was negative.

If following malaria challenge and prior to malaria diagnosis and treatment, a participant is found to have a positive SARS-COV-2 test from any source, they will be commenced on malaria treatment, irrespective of the severity of COVID-19 disease and irrespective of malaria symptoms or malaria qPCR result at that time point.

9.5 Malaria management

Volunteers will be treated with a standard oral course of either artemether-lumefantrine (Riamet®) or atavoquone/proguanil (Malarone). Both are recommended treatment of *P. vivax* and *P. falciparum* infection in the UK, although use for non-falciparum malaria is an unlicensed use for both medications. The decision on which medication will be used, will be at the Investigator's discretion and depends on factors including if there are any contraindications to either medication.

If a volunteer withdraws/is withdrawn from the study after administration of CHMI but before reaching the criteria for malaria treatment, then a complete, appropriate, curative course of anti-malarial therapy must be completed. The importance of this will be emphasised to volunteers at screening.

9.5.1 Malaria management – Riamet

Artemisinin-based combination therapies (ACTs) containing mefloquine, piperaquine or lumefantrine are now the recommended treatment for *P. vivax* in areas where there might be chloroquine resistance (WHO 2015 guidelines). Although chloroquine remains effective in most recent studies in Thailand, there are some signs that low level chloroquine resistance may be becoming established along the Thai-Myanmar border.

Riamet® is a combination drug consisting of 20mg artemether and 120mg lumefantrine per tablet. The treatment regime will consist of 6 doses of total 80mg artemether/480mg lumefantrine (4 tablets) – to clear the blood-stage infection. The first dose will be directly observed, at treatment initiation, followed by additional doses after 8, 24, 36, 48 and 60 hours (window period +/- 1 hour for each dose). The 24 hour dose will also be directly observed, and volunteers will record the time that intervening doses were taken at home in a medication diary.

Prior to starting Riamet®, volunteers will be screened for drug interactions and contraindications. This includes checking for pregnancy by urine pregnancy test (serum β-hCG test in female volunteers at C+7 and C+14) and prolonged QT (on pre-challenge ECG). Volunteers will be reminded of the potential side effects and given the patient information sheet for Riamet® and a card outlining when their doses of Riamet should be taken. Volunteers will be advised to avoid grapefruit juice. Tablets should be taken together with a fatty meal (a light snack will provided when doses are observed in clinic). At least two doses for each volunteer will be directly observed in clinic.

9.5.1 Malaria management – Malarone

Malarone (see SmPc for Malarone) is a combination drug consisting of proguanil hydrochloride and atovaquone. A treatment course of Malarone consists of 4 'standard tablets' of Malarone (proguanil hydrochloride 100 mg, atovaquone 250 mg) once daily, orally for 3 days. Two out of three doses of Malarone will be directly observed in clinic. Prior to starting Malarone, volunteers will be screened for drug interactions and contraindications (including a serum β-hCG test in female volunteers). Volunteers will be reminded of the potential side effects of Malarone and given the patient information sheet for Malarone.

9.5.2 Malaria management – supportive medications

Provided there are no contraindications, volunteers will be provided with both paracetamol (1 g orally up to four times a day) and a course of cyclizine (50 mg orally three times a day) for symptomatic relief. (See SmPC for Paracetamol & Cyclizine). Participants may be provided with an appropriate, licensed alternative anti-emetic to cyclizine if they are unable to take cyclizine.

Volunteers will be given the patient information sheet for these medications and advised how frequently they can take doses. Volunteers will be issued with a medication diary card on which they will be asked to document all doses of medications taken post-CHMI.

All medications used in the trial will be handled and dispensed according to SOP VC021: Handling, Storage and Dispensing of Non-IMP Medication.

9.5.3 Follow-up after commencing malaria treatment

In VAC069A-C: at each visit at 24 hours and 48 hours after initiation of malaria treatment, procedures will be performed according to Tables 10a to 10c, including physical observations, assessment of AEs and venepuncture for blood tests including measurement of malaria qPCR. Once daily malaria qPCR blood tests will continue until the volunteer has two consecutive qPCR readings with substantial reduction in genome copies per mL. After completing the course of anti-malarials, on day 3 after initiation of treatment, participants will be reviewed again in clinic and undergo physical observations, assessment for AEs and venepuncture.

For VAC069D-E: post treatment visits will occur at 24 hours and 72 hours after initiation of treatment. During VAC069C a number of volunteers had an additional qPCR test on T+3 as the malaria parasite count at T+2 was still positive for some participants. All T+3 malaria qPCRs results were significantly reduced. In order to minimise the number of visits, the T+2 visit has been removed. Procedures will be performed according to Table 10d to 10e, including physical observations, assessment of AEs and venepuncture for blood tests including measurement of malaria qPCR. Malaria qPCR blood tests can be repeated if qPCR readings are not substantially reduced by T+3.

9.5.3 Criteria for inpatient transfer to the NHS

If any of the following criteria are met, admission under the care of the Infectious Diseases Team, Oxford University Hospitals NHS Foundation Trust will be considered:

- Failure of symptoms to improve within 48 hours of starting anti-malarial therapy.
- Unable to tolerate oral antimalarial therapy.
- Dehydration requiring intravenous fluid therapy.
- Signs or symptoms suggestive of pulmonary oedema.
- Signs or symptoms of neurological dysfunction including altered consciousness.
- Signs, symptoms or laboratory evidence of significant renal dysfunction.
- Unanticipated concern about subject's home circumstances.
- Any other significant finding which the Investigators feels warrant inpatient admission - this includes symptoms or signs of suspected worsening COVID-19 disease e.g. respiratory failure, in the unlikely event of a participant testing PCR positive for COVID-19 during the post-challenge period.

Ultimately, the decision regarding admission will be taken by the Investigators in conjunction with the Infectious Diseases Consultant on call.

9.6 Safety measures for conduct of CHMI

Volunteer safety is of paramount importance. The following measures are in place to safeguard volunteer safety:

- Volunteers will only be enrolled in the study if both Investigators and the volunteers' GP feel this is appropriate.
- Volunteers' understanding of the trial information will be tested by means of a questionnaire at screening. This provides further confidence that fully informed consent has been obtained.
- If the subject does not have their own mobile telephone they will be issued with one for the duration of the study and counselled about the importance of keeping it switched on or checking the messages regularly.
- Before challenge, full contact details for each subject will be documented, including home address and mobile telephone numbers. Mobile telephone numbers will be verified prior to challenge to ensure the volunteers are easily contactable. Home and work landline telephone numbers where available and next-of-kin address and telephone numbers will also be documented. Subjects must also provide the Investigators with the name and 24 hour telephone number of a close friend, relative or housemate who lives nearby and will be kept informed of their whereabouts for the duration of the study.
- On the day of challenge volunteers will be provided with a medic alert card containing contact details for the study team and brief details of the study including the optimal treatment for *P. vivax* or *P. falciparum*.
- Volunteers will be able to contact a medically qualified member of the study team 24 hours a day throughout the study period and will be instructed to contact the Investigator immediately should they manifest any signs or symptoms they perceive as serious.
- If necessary, the study team will visit volunteers in their own homes if they are unable to attend clinic for review.
- At least two doses of Riamet® for each volunteer will be observed by the study team. (For volunteers taking Malarone, two out of three doses will be observed).
- Volunteers will be counselled that should they fail to return for treatment having been infected with malaria they could become very unwell and potentially die. They will be instructed to remain in Oxford and the immediate surrounding area for the duration of the intensive follow-up schedule (days 0-21 post-challenge). They will be informed that should they fail to attend a scheduled clinic visit post-challenge, their nominated contact, next of kin and the police may be informed and a search started.

9.6.1 Measures to be taken if a volunteer goes missing post-CHMI

In the unlikely event that a volunteer should (a) fail to attend for a scheduled clinical visit or (b) be un-contactable by telephone after challenge and before completion of an appropriate course of anti-malarial therapy, the following stakeholders will be informed;

- All Investigators.
- The volunteer's nominated contact and next of kin.
- The trial Sponsor.
- The local safety committee.
- The ethical committee(s).
- Relevant hospital trust R&D departments.
- The local police department.
- Local Accident and Emergency departments.

Active efforts will be made to locate the volunteer by the police. While all parties will aim to preserve the volunteer's confidentiality, if necessary details of the volunteer's identity and participation in the study may be passed to the national media in order to help locate the missing individual. Volunteers will be informed of this during screening.

9.7 Follow-up post-treatment

9.7.1 Six days after initiation of treatment (T+6)

Physical observations will be performed and AEs assessed. Venepuncture will be performed (Tables 10). Where applicable, medication diary cards and malaria symptom diary cards will be collected from volunteers.

9.7.2 Nine days after initiation of treatment (for VAC069C-D) (T+9 (+2))

Volunteers will be reviewed by telephone to check for full resolution of malaria symptoms and any side effects from malaria medication.

9.7.3 Ten days after initiation of treatment (for VAC069E) (T+10 (±2))

Physical observations will be performed and AEs assessed. Venepuncture will be performed (as per Table 10e). Where applicable, medication diary cards, malaria symptom diary cards and unused, dispensed medications will be collected from volunteers.

9.7.4 Days 28, 45 and 90 post CHMI (for VAC069A)Physical observations will be performed and AEs assessed. Venepuncture will be performed (as per Table 10a). Where applicable, medication diary cards, malaria symptom diary cards and unused, dispensed medications will be collected from volunteers.

9.7.5 Days 56 and 96 post CHMI (for VAC069B-E) (C+56 (±14) and C+96 (±14))

Physical observations will be performed and AEs assessed. Venepuncture will be performed (as per Tables 10b-e). Where applicable, medication diary cards, malaria symptom diary cards and unused, dispensed medications will be collected from volunteers.

Time windows for these visits have been increased to allow any potential participants who are self-isolating due to COVID-19, to attend the study visits once they have ended self-isolation.

9.8 Secondary and tertiary blood-stage CHMI

All volunteers, except those in Groups 10 and 12, will receive a second and third blood-stage challenge, using a 1:10 dose of inoculum selected from VAC069A, approximately 5 to 10 months after the preceding CHMI. Following a re-screening visit, as detailed in Section 9.3.2, the procedures followed will be the same as for the initial challenge (as described in Sections 9.3.3-9.7.3 and Tables 5-10 inclusive).

Volunteers who underwent CHMI with *P. vivax* in Groups 10 and 12 in VAC069D will undergo a repeat (secondary or tertiary) blood-stage challenge with *P. falciparum* at approximately 8-14 months after the preceding CHMI. Procedures will be as described above for VAC069E.

**Day**	**S/RS**	**C-1**
**Attendance number**	**1**	**2**
**Timeline (days) (window)**	**-90 (-90—14)**	**-1**
Inclusion/Exclusion criteria	X	
Informed consent	X	
Informed consent questionnaire	X	
Physical observations*	X	X
Medical history/examination	X	(X)
Electrocardiogram	X	
Urinalysis	X	
Review contraindications		X
Urine β-hCG (females only)	X	
Serum βhCG (mL) (females only)		5
Haematology, G6PD*** and haemoglobinopathy screen*** (mL)	4	
Haematology (mL)	2	2
Biochemistry (mL)**	3	3
HIV, Hepatitis B, Hepatitis C, EBV, CMV serology (mL)***	5	
HLA typing***		4
Immunology plasma (mL)		30
Immunology serum (mL)		40
Safety serum (mL)		4
Plasma for exploratory studies (mL)		3
Gene expression profiling (mL)		20
qPCR & gametocyte PCR		3
Blood film (mL)		1
**Visit Total Blood Volume (mL)**	**14**	**115**
**Cumulative blood volume (mL)**	**14**	**129**

Table 5a: Schedule of events for VAC069A&B pre-CHMI *(timeline/window are in relation to the day of challenge).* ***S*** *= screening visit;* ***RS*** *= re-screening visit;* ***(X)*** *= If considered necessary, emphasising any acute complaints. *Physical observations include blood pressure, pulse and temperature, plus height and weight at screening/re-screening and C-1 visit for VAC069A&B.* ********Biochemistry will include sodium, potassium, urea, creatinine, albumin, liver function test, plus magnesium & calcium at re-screening/screening only.* *********To be tested at initial screening visits only, i.e. will not be performed at re-screening prior to second and third challenges.*

**Day**	**S/RS**	**C-2**
**Attendance number**	**1**	**2**
**Timeline (days) (window)**	**-90 (-90 to -7)**	**-2 (-3 to -1)**
Inclusion/Exclusion criteria	X	
Informed consent	X	
Informed consent questionnaire	X	
Physical observations*	X	X
Medical history/examination	X	(X)
Electrocardiogram	X	
Urinalysis	X	
Review contraindications		X
Urine β-hCG (females only)	X	
Serum βhCG (mL) (females only)		5
Haematology#, G6PD***, haemoglobinopathy screen*** (mL)	4	2
DARC (mL)***	2	
Biochemistry (mL)**	3	3
HIV, Hepatitis B, Hepatitis C, EBV, CMV serology (mL)	5	
HLA typing***		4
Safety serum (mL)		4
Exploratory Assays (mL)		85
qPCR & gametocyte PCR		2
SARS-COV-2 test		X
**Visit Total Blood Volume (mL)**	**14**	**105**
**Cumulative blood volume (mL)**	**14**	**119**

Table 5b: Schedule of events for VAC069C-E pre-CHMI *(timeline/window are in relation to the day of challenge). **S** = screening visit; **RS** = re-screening visit**; (X)** = If considered necessary, emphasising any acute complaints. *Physical observations include blood pressure, pulse and temperature, plus height and weight at screening/re-screening and weight at C-2 visit for VAC069C-E. **Biochemistry will include sodium, potassium, urea, creatinine, albumin, liver function test, plus magnesium & calcium at re-screening/screening only. ***To be tested at initial screening visits only, i.e. will not be performed at re-screening prior to second and third challenges. # FBC to include reticulocyte count at C-2 visit*

**Day**	**C**	**C+1**	**C+2**	**C+2.5**	**C+3**	**C+3.5**	**C+4**	**C+4.5**	**C+5**	**C+5.5**	**C+6**	**C+6.5**	**C+7**	**C+7.5**
**Attendance number**	**3**	**4**	**5**	**6**	**7**	**8**	**9**	**10**	**11**	**12**	**13**	**14**	**15**	**16**
**Timeline (days) (window)**	**0**	**1**	**2**	**2**	**3**	**3**	**4**	**4**	**5**	**5**	**6**	**6**	**7**	**7**
Physical observations*	X	X	X	X	X	X	X	X	X	X	X	X	X	X
Medical history/examination	(X)	(X)	(X)	(X)	(X)	(X)	(X)	(X)	(X)	(X)	(X)	(X)	(X)	(X)
Review contraindications	X													
Review Aes & medications		X	X	X	X	X	X	X	X	X	X	X	X	X
Medic alert card provided	X													
Diary card issued	X													
Serum βhCG (mL) (females only)													5	
Haematology (mL)													2	
Biochemistry (mL)**													3	
Immunology plasma (mL)													30	
Immunology serum (mL)													40	
Gene expression profiling (mL)											15		15	
qPCR & gametocyte PCR (mL)		3	3	3	3	3	3	3	3	3	3	3	3	3
Blood film(mL)		1	1	1	1	1	1	1	1	1	1	1	1	1
**Visit Total Blood Volume (mL)**		**4**	**4**	**4**	**4**	**4**	**4**	**4**	**4**	**4**	**19**	**4**	**99**	4
**Cumulative blood volume (mL)**		**133**	**137**	**141**	**145**	**149**	**153**	**157**	**161**	**165**	**184**	**188**	**287**	291

Table 6a: Schedule of events for day of challenge to day 7 after challenge for volunteers in VAC069A *(timeline/window are in relation to the day of challenge). **C** = challenge; **(X)** = If considered necessary. Visits continue according to schedule until diagnostic criteria are reached; when diagnostic criteria are met, venepuncture and procedures are followed according to Table 10a.*Physical observations include blood pressure, pulse and temperature. **Biochemistry comprises sodium, potassium, urea, creatinine, albumin, liver function tests.*

**Day**	**C**	**C+1**	**C+2**	**C+3**	**C+4**	**C+5**	**C+6**	**C+7**
**Attendance number**	**3**	**-**	**-**	**-**	**-**	**-**	**4**	**5**
**Timeline (days) (window)**	**0**	**1**	**2**	**3**	**4**	**5**	**6**	**7**
Physical observations*	X						X	X
Medical history/examination	(X)						X	X
Review contraindications	X							
Daily phone call		X	X	X	X	X		
Review AEs & medications		X	X	X	X	X	X	X
Medic alert card provided	X							
Diary card issued	X							
Serum βhCG (mL) (females only)								5
Haematology (mL)								2
Biochemistry (mL)**								3
Immunology plasma (mL)								30
Immunology serum (mL)								30
Gene expression profiling EDTA sample (mL)							9	
Gene expression profiling Li Heparin sample (mL)							3	
qPCR & gametocyte PCR (mL)							3	3
Blood film(mL)							1	1
**Visit Total Blood Volume (mL)**	**-**	**-**	**-**	**-**	**-**	**-**	**16**	**74**
**Cumulative blood volume (mL)**	**129**	**129**	**129**	**129**	**129**	**129**	**145**	**219**

Table 6b: Schedule of events for day of challenge – day 7 after challenge for volunteers in VAC069B*(timeline/window are in relation to the day of challenge).* ***C*** *= challenge;* ***(X)*** *= If considered necessary. Visits continue according to schedule until diagnostic criteria are reached; when diagnostic criteria are met, venepuncture and procedures are followed according to Table 10b.*Physical observations include blood pressure, pulse and temperature. **Biochemistry comprises sodium, potassium, urea, creatinine, albumin, liver function tests.*

Day	C	C+1	C+2	C+3	C+4	C+5	C+6	C+7
**Attendance number**	**3**	**-**	**-**	**-**	**-**	**-**	**-**	**4**
**Timeline (days) (window)**	**0**	**1**	**2**	**3**	**4**	**5**	**6**	**7**
Physical observations*	X							X
Medical history/examination	(X)							X
Review contraindications	X							
Daily phone call		X	X	X	X	X	X	
Review AEs & medications		X	X	X	X	X	X	X
Medic alert card provided	X							
Diary card issued	X							
Serum βhCG (mL) (females only)								5
Haematology (mL)								2
Biochemistry (mL)**								3
Exploratory assays (mL)								60
Gene expression profiling EDTA sample (mL)								
Gene expression profiling Li Heparin sample (mL)								
qPCR & gametocyte PCR (mL)								2
**Visit Total Blood Volume (mL)^**	-	**-**	**-**	**-**	**-**	**-**	**-**	**72**
**Cumulative blood volume (mL)**	119	**119**	**119**	**119**	**119**	**119**	**119**	**191**

Table 6c: Schedule of events for day of challenge – day 7 after challenge for volunteers in VAC069C-D *(timeline/window are in relation to the day of challenge). **C** = challenge; **(X)** = If considered necessary. Visits continue according to schedule until diagnostic criteria are reached; when diagnostic criteria are met, venepuncture and procedures are followed according to Table 10c.*Physical observations include blood pressure, pulse and temperature. **Biochemistry comprises sodium, potassium, urea, creatinine, albumin, liver function tests. ^Blood volumes as given in the table are maximal volumes and smaller blood volumes may be taken at any visit, at the discretion of the investigator, with the exception of additional blood volume that may be required for any additional tests that may be performed if deemed clinically necessary.*

Day	C	C+1	C+2	C+3	C+4	C+5	C+6	C+7
**Attendance number**	**3**	**-**	**-**	**-**	**-**	**4**	**5**	**6**
**Timeline (days) (window)**	**0**	**1**	**2**	**3**	**4**	**5**	**6**	**7**
Physical observations*	X					X	X	X
Medical history/examination	(X)					X	X	X
Review contraindications	X							
Daily phone call		X	X	X	X			
Review AEs & medications		X	X	X	X	X	X	X
Medic alert card provided	X							
Diary card issued	X							
Serum βhCG (mL) (females only)								5
Haematology (mL)								2
Biochemistry (mL)**								3
Exploratory assays (mL)								22
qPCR & gametocyte PCR (mL)						2	2	2
**Visit Total Blood Volume (mL)^**	**-**	**-**	**-**	**-**	**-**	**2**	**2**	**34**
**Cumulative blood volume (mL)**	**119**	**119**	**119**	**119**	**119**	**121**	**123**	**157**

Table 6d: Schedule of events for day of challenge – day 7 after challenge for volunteers in VAC069E *(timeline/window are in relation to the day of challenge).* ***C*** *= challenge;* ***(X)*** *= If considered necessary. Visits continue according to schedule until diagnostic criteria are reached; when diagnostic criteria are met, venepuncture and procedures are followed according to Table 10d.*Physical observations include blood pressure, pulse and temperature. **Biochemistry comprises sodium, potassium, urea, creatinine, liver function tests. ^Blood volumes as given in the table are maximal volumes and smaller blood volumes may be taken at any visit, at the discretion of the investigator, with the exception of additional blood volume that may be required for any additional tests that may be performed if deemed clinically necessary.*

Day	C+8	C+8.5	C+9	C+9.5	C+10	C+10.5	C+11	C+11.5	C+12	C+12.5	C+13	C+13.5[a]	C+14	C+14.5[a]
**Attendance number**	**17**	**18**	**19**	**20**	**21**	**22**	**23**	**24**	**25**	**26**	**27**	**28**	**29**	**30**
**Timeline (days) (window)**	**8**	**8**	**9**	**9**	**10**	**10**	**11**	**11**	**12**	**12**	**13**	**13**	**14**	**14**
Physical observations*	X	X	X	X	X	X	X	X	X	X	X	X	X	X
Medical history/examination	(X)	(X)	(X)	(X)	(X)	(X)	(X)	(X)	(X)	(X)	(X)	(X)	(X)	(X)
Review Aes & medications	X	X	X	X	X	X	X	X	X	X	X	X	X	X
Serum βhCG (mL) (females only)													5	
Haematology (mL)													2	
Biochemistry (mL)**													3	
Immunology (mL)													30	
Immunology serum (mL)													40	
Gene expression profiling (mL)	15		15		15		15		15		15		15	
qPCR & gametocyte PCR (mL)	3	3	3	3	3	3	3	3	3	3	3	3	3	3
Blood film(mL)	1	1	1	1	1	1	1	1	1	1	1	1	1	1
**Visit Total Blood Volume (mL)**	**19**	**4**	**19**	**4**	**19**	**4**	**19**	**4**	**19**	**4**	**19**	**4**	**99**	**4**
**Cumulative blood volume (mL)**	**310**	**314**	**333**	**337**	**356**	**360**	**379**	**383**	**402**	**406**	**425**	**429**	**528**	**532**

Table 7a: Schedule of events for days 8-14 after challenge for volunteers in VAC069A *(timeline/window are in relation to the day of challenge).* ***C*** *= challenge;* ***(X)*** *= If considered necessary. Visits continue according to schedule until diagnostic criteria are reached; when diagnostic criteria are met, venepuncture and procedures are followed according to Table 10a.*Physical observations include blood pressure, pulse and temperature. **Biochemistry comprises sodium, potassium, urea, creatinine, albumin, liver function tests. [a]From C+13, visits will once daily only if qPCR <1000 genome copies/mL, i.e. C+13.5 and C+14.5 visits will be omitted if this threshold has not been reached.*

**Day**	**C+8**	**C+9**	**C+10**	**C+10.5**	**C+11**	**C+11.5**	**C+12**	**C+12.5**	**C+13**	**C+13.5**	**C+14**	**C+14.5**
**Attendance number**	**6**	**7**	**8**	**9**	**10**	**11**	**12**	**13**	**14**	**15**	**16**	**17**
**Timeline (days) (window)**	**8**	**9**	**10**	**10**	**11**	**11**	**12**	**12**	**13**	**13**	**14**	**14**
Physical observations*	X	X	X	X	X	X	X	X	X	X	X	X
Medical history/examination	(X)	(X)	(X)	(X)	(X)	(X)	(X)	(X)	(X)	(X)	(X)	(X)
Review AEs & medications	X	X	X	X	X	X	X	X	X	X	X	X
Serum βhCG (mL) (Females only)											5	
Haematology (mL)											2	
Biochemistry (mL)**											3	
Immunology plasma (mL)											30	
Immunology serum (mL)											30	
Gene expression profiling EDTA sample (mL)	9	3	9		3		9		3		9	
Gene expression profiling Li Heparin sample (mL)	3		3				3				3	
DMFA (mL)											10	
Direct feeding assay (mosquito feeding)											X	
qPCR & gametocyte PCR (mL)	3	3	3	3	3	3	3	3	3	3	3	3
Blood film(mL)	1	1	1	1	1	1	1	1	1	1	1	1
**Visit Total Blood Volume (mL)**	**16**	**7**	**16**	**4**	**7**	**4**	**16**	**4**	**7**	**4**	**96**	**4**
**Cumulative blood volume (mL)**	**235**	**242**	**258**	**262**	**269**	**273**	**289**	**293**	**300**	**304**	**400**	**404**

Table 7b: Schedule of events for days 8-14 after challenge for volunteers in VAC069B *(timeline/window are in relation to the day of challenge).* ***C*** *= challenge;* ***(X)*** *= If considered necessary. Visits continue according to schedule until diagnostic criteria are reached; when diagnostic criteria are met, venepuncture and procedures are followed according to Table 10b.*Physical observations include blood pressure, pulse and temperature. **Biochemistry comprises sodium, potassium, urea, creatinine, albumin, liver function tests.*

**Day**	**C+8**	**C+9**	**C+10**	**C+10.5**	**C+11**	**C+11.5**	**C+12**	**C+12.5**	**C+13**	**C+13.5**	**C+14**	**C+14.5**
**Attendance number**	**5**	**6**	**7**	**8**	**9**	**10**	**11**	**12**	**13**	**14**	**15**	**16**
**Timeline (days) (window)**	**8**	**9**	**10**	**10**	**11**	**11**	**12**	**12**	**13**	**13**	**14**	**14**
Physical observations*	X	X	X	X	X	X	X	X	X	X	X	X
Medical history/examination	(X)	(X)	(X)	(X)	(X)	(X)	(X)	(X)	(X)	(X)	(X)	(X)
Review AEs & medications	X	X	X	X	X	X	X	X	X	X	X	X
Serum βhCG (mL) (females only)											5	
Haematology (mL)											2	
Biochemistry (mL)**											3	
Exploratory Assays (mL)	12		12				12				82	
DMFA (mL)											10	
Direct feeding assay (mosquito feeding)											X	
qPCR & gametocyte PCR (mL)	2	2	2	2	2	2	2	2	2	2	2	2
**Visit Total Blood Volume (mL)^**	**14**	**2**	**14**	**2**	**2**	**2**	**14**	**2**	**2**	**2**	**94**	**2**
**Cumulative blood volume (mL)**	**205**	**207**	**221**	**223**	**225**	**227**	**241**	**243**	**245**	**247**	**341**	**343**

Table 7c: Schedule of events for days 8-14 after challenge for volunteers in VAC069C *(timeline/window are in relation to the day of challenge).* ***C*** *= challenge;* ***(X)*** *= If considered necessary. Visits continue according to schedule until diagnostic criteria are reached; when diagnostic criteria are met, venepuncture and procedures are followed according to Table 10c.*Physical observations include blood pressure, pulse and temperature. **Biochemistry comprises sodium, potassium, urea, creatinine, albumin, liver function tests. ^Blood volumes as given in the table are maximal volumes and smaller blood volumes may be taken at any visit, at the discretion of the investigator, with the exception of additional blood volume that may be required for any additional tests that may be performed if deemed clinically necessary.*

**Day**	**C+8**	**C+9**	**C+10**	**C+10.5$**	**C+11**	**C+11.5$**	**C+12**	**C+12.5$**	**C+13**	**C+13.5$**	**C+14**	**C+14.5$**
**Attendance number**	**5**	**6**	**7**	**8**	**9**	**10**	**11**	**12**	**13**	**14**	**15**	**16**
**Timeline (days) (window)**	**8**	**9**	**10**	**10**	**11**	**11**	**12**	**12**	**13**	**13**	**14**	**14**
Physical observations*	X	X	X	X	X	X	X	X	X	X	X	X
Medical history/examination	(X)	(X)	(X)	(X)	(X)	(X)	(X)	(X)	(X)	(X)	(X)	(X)
Review AEs & medications	X	X	X	X	X	X	X	X	X	X	X	X
Serum βhCG (mL) (females only)											5	
Haematology (mL)											2	
Biochemistry (mL)**											3	
Exploratory Assays (mL)							12				82	
DMFA (mL)												
Direct feeding assay (mosquito feeding)											X	
qPCR & gametocyte PCR (mL)	2	2	2	2	2	2	2	2	2	2	2	2
**Visit Total Blood Volume (mL)^**	**2**	**2**	**2**	**2**	**2**	**2**	**14**	**2**	**2**	**2**	**94**	**2**
**Cumulative blood volume (mL)**	**193**	**195**	**197**	**199**	**201**	**203**	**217**	**219**	**221**	**223**	**317**	**319**

Table 7d: Schedule of events for days 8-14 after challenge for volunteers in VAC069D *(timeline/window are in relation to the day of challenge).* ***C*** *= challenge;* ***(X)*** *= If considered necessary. Visits continue according to schedule until diagnostic criteria are reached; when diagnostic criteria are met, venepuncture and procedures are followed according to Table 10d. $From day 10 post challenge onwards, visits will continue once a day in mornings, increasing to twice daily if the malaria qPCR count is >1000 genome copies/ml. *Physical observations include blood pressure, pulse and temperature. **Biochemistry comprises sodium, potassium, urea, creatinine, albumin, liver function tests. ^Blood volumes as given in the table are maximal volumes and smaller blood volumes may be taken at any visit, at the discretion of the investigator, with the exception of additional blood volume that may be required for any additional tests that may be performed if deemed clinically necessary.*

Day	C+7.5$	C+8	C+8.5$	C+9	C+9.5$	C+10	C+10.5$	C+11	C+11.5$	C+12	C+12.5$	C+13	C+13.5$	C+14	C+14.5$
**Attendance number**	**6**	**7**	**8**	**9**	**10**	**11**	**12**	**13**	**14**	**15**	**16**	**17**	**18**	**19**	**20**
**Timeline (days) (window)**	**7**	**8**	**8**	**9**	**9**	**10**	**10**	**11**	**11**	**12**	**12**	**13**	**13**	**14**	**14**
Physical observations*	X	X	X	X	X	X	X	X	X	X	X	X	X	X	X
Medical history/examination	(X)	(X)	(X)	(X)	(X)	(X)	(X)	(X)	(X)	(X)	(X)	(X)	(X)	(X)	(X)
Review AEs & medications	X	X	X	X	X	X	X	X	X	X	X	X	X	X	X
Serum βhCG (mL) (females only)														5	
Haematology (mL)														2	
Biochemistry (mL)**														3	
Exploratory Assays (mL)				12				12				12		10	
qPCR & gametocyte PCR (mL)	2	2	2	2	2	2	2	2	2	2	2	2	2	2	2
**Visit Total Blood Volume (mL)^**	**2**	**2**	**2**	**14**	**2**	**2**	**2**	**14**	**2**	**2**	**2**	**14**	**2**	**22**	**2**
**Cumulative blood volume (mL)**	**159**	**161**	**163**	**177**	**179**	**181**	**183**	**197**	**199**	**201**	**203**	**217**	**219**	**241**	**243**

Table 7e: Schedule of events for days 8-14 after challenge for volunteers in VAC069E *(timeline/window are in relation to the day of challenge).* ***C*** *= challenge;* ***(X)*** *= If considered necessary. Visits continue according to schedule until diagnostic criteria are reached; when diagnostic criteria are met, venepuncture and procedures are followed according to Table 10e. $From day 7 post challenge onwards, visits will continue once a day in mornings, increasing to twice daily if the malaria qPCR count is >1000 genome copies/ml. *Physical observations include blood pressure, pulse and temperature. **Biochemistry comprises sodium, potassium, urea, creatinine, liver function tests. ^Blood volumes as given in the table are maximal volumes and smaller blood volumes may be taken at any visit, at the discretion of the investigator, with the exception of additional blood volume that may be required for any additional tests that may be performed if deemed clinically necessary.*

**Day**	**C+15**	**C+15.5[a]**	**C+16**	**C+16.5[a]**	**C+17**	**C+17.5[a]**	**C+18**	**C+18.5[a]**	**C+19**	**C+19.5[a]**	**C+20**	**C+20.5[a]**
**Attendance number**	**31**	**32**	**33**	**34**	**35**	**36**	**37**	**38**	**39**	**40**	**41**	**42**
**Timeline (days) (window)**	**15**	**15**	**16**	**16**	**17**	**17**	**18**	**18**	**19**	**19**	**20**	**20**
Physical observations	X	X	X	X	X	X	X	X	X	X	X	X
Medical history/examination	(X)	(X)	(X)	(X)	(X)	(X)	(X)	(X)	(X)	(X)	(X)	(X)
Review Aes & medications	X	X	X	X	X	X	X	X	X	X	X	X
Gene expression profiling (mL)	15		15		15		15		15		15	
qPCR & gametocyte PCR (mL)	3	3	3	3	3	3	3	3	3	3	3	3
Blood film(mL)	1	1	1	1	1	1	1	1	1	1	1	1
**Visit Total Blood Volume (mL)**	**19**	**4**	**19**	**4**	**19**	**4**	**19**	**4**	**19**	**4**	**19**	**4**
**Cumulative blood volume (mL)**	**551**	**555**	**574**	**578**	**597**	**601**	**620**	**624**	**643**	**647**	**666**	**670**

Table 8a: Schedule of events for days 15-20 after challenge for volunteers in VAC069A *(timeline/window are in relation to the day of challenge).* ***C*** *= challenge;* ***(X)*** *= If considered necessary. Visits continue according to schedule until diagnostic criteria are reached; when diagnostic criteria are met, venepuncture and procedures are followed according to Table 10a.*Physical observations include blood pressure, pulse and temperature. **Biochemistry comprises sodium, potassium, urea, creatinine, albumin, liver function tests. [a]From C+13, visits will once daily only if qPCR <1000 genome copies/mL, i.e. C+15.5, C+16.5, C+17.5, C+18.5, C+19.5 and C+20.5 visits will be omitted if this threshold has not been reached .*

Day	C+15	C+15.5	C+16	C+16.5	C+17	C+17.5	C+18	C+18.5	C+19	C+19.5	C+20	C+20.5
**Attendance number**	**18**	**19**	**20**	**21**	**22**	**23**	**24**	**25**	**26**	**27**	**28**	**29**
**Timeline (days) (window)**	**15**	**15**	**16**	**16**	**17**	**17**	**18**	**18**	**19**	**19**	**20**	**20**
Physical observations	X	X	X	X	X	X	X	X	X	X	X	X
Medical history/examination	(X)	(X)	(X)	(X)	(X)	(X)	(X)	(X)	(X)	(X)	(X)	(X)
Review AEs & medications	X	X	X	X	X	X	X	X	X	X	X	X
Immunology plasma (mL)												
Immunology serum (mL)												
Gene expression profiling EDTA sample (mL)	3		9		3		9		3		9	
Gene expression profiling Li Heparin sample (mL)			3				3				3	
qPCR & gametocyte PCR (mL)	3	3	3	3	3	3	3	3	3	3	3	3
Blood film(mL)	1	1	1	1	1	1	1	1	1	1	1	1
**Visit Total Blood Volume (mL)**	**7**	**4**	**16**	**4**	**7**	**4**	**16**	**4**	**7**	**4**	**16**	**4**
**Cumulative blood volume (mL)**	**411**	**415**	**431**	**435**	**442**	**446**	**462**	**466**	**473**	**477**	**493**	**497**

Table 8b: Schedule of events for days 15-20 after challenge for volunteers in VAC069B *(timeline/window are in relation to the day of challenge).* ***C*** *= challenge;* ***(X)*** *= If considered necessary. Visits continue according to schedule until diagnostic criteria are reached; when diagnostic criteria are met, venepuncture and procedures are followed according to Table 10b.*Physical observations include blood pressure, pulse and temperature. **Biochemistry comprises sodium, potassium, urea, creatinine, albumin, liver function tests. ªFrom C+13, visits will once daily only if qPCR <1000 genome copies/mL, i.e. C+15.5, C+16.5, C+17.5, C+18.5, C+19.5 and C+20.5 visits will be omitted if this threshold has not been reached .*

**Day**	**C+15**	**C+15.5**	**C+16**	**C+16.5**	**C+17**	**C+17.5**	**C+18**	**C+18.5**	**C+19**	**C+19.5**	**C+20**	**C+20.5**
**Attendance number**	**17**	**18**	**19**	**20**	**21**	**22**	**23**	**24**	**25**	**26**	**27**	**28**
**Timeline (days) (window)**	**15**	**15**	**16**	**16**	**17**	**17**	**18**	**18**	**19**	**19**	**20**	**20**
Physical observations	X	X	X	X	X	X	X	X	X	X	X	X
Medical history/examination	(X)	(X)	(X)	(X)	(X)	(X)	(X)	(X)	(X)	(X)	(X)	(X)
Review AEs & medications	X	X	X	X	X	X	X	X	X	X	X	X
Exploratory Assays (mL)			12				12				12	
qPCR & gametocyte PCR (mL)	2	2	2	2	2	2	2	2	2	2	2	2
**Visit Total Blood Volume (mL)^**	**2**	**2**	**14**	**2**	**2**	**2**	**14**	**2**	**2**	**2**	**14**	**2**
**Cumulative blood volume (mL)**	**345**	**347**	**361**	**363**	**365**	**367**	**381**	**383**	**385**	**387**	**401**	**403**

Table 8c: Schedule of events for days 15-20 after challenge for volunteers in VAC069C *(timeline/window are in relation to the day of challenge).* ***C*** *= challenge;* ***(X)*** *= If considered necessary. Visits continue according to schedule until diagnostic criteria are reached; when diagnostic criteria are met, venepuncture and procedures are followed according to Table 10c.*Physical observations include blood pressure, pulse and temperature. **Biochemistry comprises sodium, potassium, urea, creatinine, albumin, liver function tests. ^Blood volumes as given in the table are maximal volumes and smaller blood volumes may be taken at any visit, at the discretion of the investigator, with the exception of additional blood volume that may be required for any additional tests that may be performed if deemed clinically necessary.*

Day	C+15	C+15.5$	C+16	C+16.5$	C+17	C+17.5$	C+18	C+18.5$	C+19	C+19.5$	C+20	C+20.5$
**Attendance number**	**17**	**18**	**19**	**20**	**21**	**22**	**23**	**24**	**25**	**26**	**27**	**28**
**Timeline (days) (window)**	**15**	**15**	**16**	**16**	**17**	**17**	**18**	**18**	**19**	**19**	**20**	**20**
Physical observations	X	X	X	X	X	X	X	X	X	X	X	X
Medical history/examination	(X)	(X)	(X)	(X)	(X)	(X)	(X)	(X)	(X)	(X)	(X)	(X)
Review AEs & medications	X	X	X	X	X	X	X	X	X	X	X	X
Exploratory Assays (mL)			12				12				12	
qPCR & gametocyte PCR (mL)	2	2	2	2	2	2	2	2	2	2	2	2
**Visit Total Blood Volume (mL)^**	**2**	**2**	**14**	**2**	**2**	**2**	**14**	**2**	**2**	**2**	**14**	**2**
**Cumulative blood volume (mL)**	**321**	**323**	**337**	**339**	**341**	**343**	**357**	**359**	**361**	**363**	**377**	**379**

Table 8d: Schedule of events for days 15-20 after challenge for volunteers in VAC069D *(timeline/window are in relation to the day of challenge).* ***C*** *= challenge;* ***(X)*** *= If considered necessary. Visits continue according to schedule until diagnostic criteria are reached; when diagnostic criteria are met, venepuncture and procedures are followed according to Table 10d. $From day 10 post challenge onwards, visits will continue once a day in mornings, increasing to twice daily if the malaria qPCR count is >1000 genome copies/ml. *Physical observations include blood pressure, pulse and temperature. **Biochemistry comprises sodium, potassium, urea, creatinine, albumin, liver function tests. ^Blood volumes as given in the table are maximal volumes and smaller blood volumes may be taken at any visit, at the discretion of the investigator, with the exception of additional blood volume that may be required for any additional tests that may be performed if deemed clinically necessary.*

**Day**	**C+15**	C+15.5$	**C+16**	C+16.5$	**C+17**	C+17.5$	**C+18**	C+18.5$	**C+19**	C+19.5$	**C+20**	C+20.5$
**Attendance number**	**21**	**22**	**23**	**24**	**25**	**26**	**27**	**28**	**29**	**30**	**31**	**32**
**Timeline (days) (window)**	**15**	**15**	**16**	**16**	**17**	**17**	**18**	**18**	**19**	**19**	**20**	**20**
Physical observations	X	X	X	X	X	X	X	X	X	X	X	X
Medical history/examination	(X)	(X)	(X)	(X)	(X)	(X)	(X)	(X)	(X)	(X)	(X)	(X)
Review AEs & medications	X	X	X	X	X	X	X	X	X	X	X	X
Exploratory Assays (mL)	12				12				12			
qPCR & gametocyte PCR (mL)	2	2	2	2	2	2	2	2	2	2	2	2
**Visit Total Blood Volume (mL)^**	**14**	**2**	**2**	**2**	**14**	**2**	**2**	**2**	**14**	**2**	**2**	**2**
**Cumulative blood volume (mL)**	**257**	**259**	**261**	**263**	**277**	**279**	**281**	**283**	**297**	**299**	**301**	**303**

Table 8e: Schedule of events for days 15-20 after challenge for volunteers in VAC069E *(timeline/window are in relation to the day of challenge).* ***C*** *= challenge;* ***(X)*** *= If considered necessary. Visits continue according to schedule until diagnostic criteria are reached; when diagnostic criteria are met, venepuncture and procedures are followed according to Table 10e. $From day 7 post challenge onwards, visits will continue once a day in mornings, increasing to twice daily if the malaria qPCR count is >1000 genome copies/ml. *Physical observations include blood pressure, pulse and temperature. **Biochemistry comprises sodium, potassium, urea, creatinine, liver function tests. ^Blood volumes as given in the table are maximal volumes and smaller blood volumes may be taken at any visit, at the discretion of the investigator, with the exception of additional blood volume that may be required for any additional tests that may be performed if deemed clinically necessary.*

**Day**	**C+21**	**C+21.5**	**C+22**	**C+22.5**	**C+23**	**C+23.5**	**C+24**	**C+24.5**	**C+25**	**C+25.5**	**C+26**	**C+26.5**	**C+27**	**C+27.5**
**Attendance number**	**30**	**31**	**32**	**33**	**34**	**35**	**36**	**37**	**38**	**39**	**40**	**41**	**42**	**43**
**Timeline (days) (window)**	**21**	**21**	**22**	**22**	**23**	**23**	**24**	**24**	**25**	**25**	**26**	**26**	**27**	**27**
Physical observations	X	X	X	X	X	X	X	X	X	X	X	X	X	X
Medical history/examination	(X)	(X)	(X)	(X)	(X)	(X)	(X)	(X)	(X)	(X)	(X)	(X)	(X)	(X)
Review AEs & medications	X	X	X	X	X	X	X	X	X	X	X	X	X	X
Serum βhCG (mL) (females only)	5													
Haematology (mL)	2													
Biochemistry (mL)**	3													
Immunology plasma (mL)	30													
Immunology serum (mL)	30													
Gene expression profiling EDTA sample (mL)	3		9		3		9		3		9		3	
Gene expression profiling Li Heparin sample (mL)			3				3				3			
qPCR & gametocyte PCR (mL)	3	3	3	3	3	3	3	3	3	3	3	3	3	3
Blood film(mL)	1	1	1	1	1	1	1	1	1	1	1	1	1	1
**Visit Total Blood Volume (mL)**	**77**	**4**	**16**	**4**	**7**	**4**	**16**	**4**	**7**	**4**	**16**	**4**	**7**	**4**
**Cumulative blood volume (mL)**	**574**	**578**	**594**	**598**	**605**	**609**	**625**	**629**	**636**	**640**	**656**	**660**	**667**	**671**

Table 9a: Schedule of events for days 21 - 27 after challenge for volunteers in VAC069B *(timeline/window are in relation to the day of challenge).* ***C*** *= challenge;* ***(X)*** *= If considered necessary. Visits continue according to schedule until diagnostic criteria are reached; when diagnostic criteria are met, venepuncture and procedures are followed according to Table 10b.*Physical observations include blood pressure, pulse and temperature. **Biochemistry comprises sodium, potassium, urea, creatinine, albumin, liver function tests.* [a] From C+21, visits may be reduced to once daily at the discretion of the Investigator (as agreed by the Chief Investigator) *i.e. C+21.5, C+22.5, C+23.5, C+24.5, C+25.5, C+26.5 and C+27.5 visits may be omitted at the Investigator's discretion*

Day	C+21	C+21.5[a]	C+22	C+22.5[a]	C+23	C+23.5[a]	C+24	C+24.5[a]	C+25	C+25.5[a]	C+26	C+26.5[a]	C+27	C+27.5[a]
**Attendance number**	**29**	**30**	**31**	**32**	**33**	**34**	**35**	**36**	**37**	**38**	**39**	**40**	**41**	**42**
**Timeline (days) (window)**	**21**	**21**	**22**	**22**	**23**	**23**	**24**	**24**	**25**	**25**	**26**	**26**	**27**	**27**
Physical observations	X	X	X	X	X	X	X	X	X	X	X	X	X	X
Medical history/examination	(X)	(X)	(X)	(X)	(X)	(X)	(X)	(X)	(X)	(X)	(X)	(X)	(X)	(X)
Review AEs & medications	X	X	X	X	X	X	X	X	X	X	X	X	X	X
Serum βhCG (mL) (females only)	5													
Haematology (mL)	2													
Biochemistry (mL)**	3													
Exploratory Assays (mL)	60		12				12				12			
qPCR & gametocyte PCR (mL)	2	2	2	2	2	2	2	2	2	2	2	2	2	2
**Visit Total Blood Volume (mL)^**	**72**	**2**	**14**	**2**	**2**	**2**	**14**	**2**	**2**	**2**	**14**	**2**	**2**	**2**
**Cumulative blood volume (mL)**	**475**	**477**	**491**	**493**	**495**	**497**	**511**	**513**	**515**	**517**	**531**	**533**	**535**	**537**

Table 9b: Schedule of events for days 21 - 27 after challenge for volunteers in VAC069C *(timeline/window are in relation to the day of challenge).* ***C*** = *challenge;* ***(X)*** *= If considered necessary. Visits continue according to schedule until diagnostic criteria are reached; when diagnostic criteria are met, venepuncture and procedures are followed according to Table 10c.*Physical observations include blood pressure, pulse and temperature. **Biochemistry comprises sodium, potassium, urea, creatinine, albumin, liver function tests.* [a] From C+21, visits may be reduced to once daily at the discretion of the Investigator (as agreed by the Chief Investigator) *i.e. C+21.5, C+22.5, C+23.5, C+24.5, C+25.5, C+26.5 and C+27.5 visits may be omitted at the Investigator's discretion ^Blood volumes as given in the table are maximal volumes and smaller blood volumes may be taken at any visit, at the discretion of the investigator, with the exception of additional blood volume that may be required for any additional tests that may be performed if deemed clinically necessary.*

Day	D/C+21 if undiagnosed[a]	T+1	T+2	T+6	C+28	C+45	C+90
**Attendance number**	**43**	**44**	**45**	**46**	**47**	**48**	**49**
**Timeline (days) (window)**					**28(±4)**	**45(±7)**	**90(±7)**
Physical observations*	X	X	X	X	X	X	X
Medical history/examination	(X)	(X)	(X)	(X)	(X)	(X)	(X)
Review Aes & medications	X	X	X	X	X	X	X
Review contraindications	X						
Diary card collected					X	(X)	(X)
Antimalarials Initiated	X						
Urine β-hCG (females only)	X						
Serum βhCG (mL) (females only)	5						
Haematology (mL)	2	2		2	2	0	2
Biochemistry (mL)**	3	3		3	3	0	3
Immunology plasma (mL)	30[b]				30		30
Immunology serum (mL)	30[b]				40	4	40
Safety serum (mL)^							4
Plasma for exploratory studies (mL)	13						
Gene expression profiling (mL)	35			15	1	20	
qPCR & gametocyte PCR (mL)	3	3	3	3	3	3	3
Blood film(mL)	1	1	1				
**Visit Total Blood Volume (mL)**	**122**	**9**	**4**	**23**	**79**	**27**	**82**
**Cumulative blood volume (mL)**	**792**	**801**	**805**	**828**	**907**	**934**	**1016**

Table 10a: Schedule of events from diagnosis to 90 days after challenge for volunteers in VAC069A *(timeline/window are in relation to the day of challenge).* ***D** = diagnosis**;** **C** = challenge; **T** = treatment;* ***(X)*** *= If considered necessary.*Physical observations include blood pressure, pulse and temperature. **Biochemistry comprises sodium, potassium, urea, creatinine, albumin, liver function tests. ^Serum will be sent for* HBsAg, HCV antibodies, HIV antibodies, CMV antibodies and EBV antibodies. *However, EBV and CMV serology will only be repeated post-challenge if the participant had negative serology results at screening. [a]Bloods to be taken immediately before drug treatment, if diagnostic criteria not met, treatment will be started at C+21 morning visit [b] If diagnosis is made at C+14.5 visit, immunology draw will not be taken, since a large volume bleed is taken for immunological analysis at morning C+14 visit*

**Day**	**D/C+28 if undiagnosed[a]**	**T+1[c]**	**T+2[c]**	**T+3**	**T+6**	**C+56**	**C+96**
**Attendance number**	**44**	**45**	**46**	**47**	**48**	**49**	**50**
**Timeline (days) (window)**	**-**	**-**	**-**	**-**	**-**	**56(±4)**	**96(±7)**
Physical observations*	X	X	X	X	X	X	X
Medical history/examination	(X)	(X)	(X)	(X)	(X)	(X)	(X)
Review AEs & medications	X	X	X	X	X	X	X
Review contraindications	X						
Diary card collected					X	(X)	(X)
Antimalarials Initiated	X						
Urine β-hCG (females only)	X						
Serum βhCG (mL) (females only)	5						
Haematology (mL)	2				2		2
Biochemistry (mL)**	3				3		3
Diagnostic Serology^							4
Immunology plasma (mL)	30[b]					30	30
Immunology serum (mL)	40[b]					40	40
Parasite cryopreservation	20						
DMFA (mL)	10						
Direct feeding assay (mosquito feeding)	X						
Plasma for exploratory studies (mL)	3						
Safety serum (mL)^							4
Gene expression profiling EDTA sample (mL)	9	9		9	9	17	
Gene expression profiling Li Heparin sample (mL)	23	3		3	3	3	
qPCR & gametocyte PCR (mL)	3	3	3				
Blood film(mL)	1	1	1				
**Visit Total Blood Volume (mL)**	**149**	**16**	**4**	**12**	**17**	**90**	**83**
**Cumulative blood volume (mL)**	**820**	**836**	**840**	**852**	**869**	**959**	**1042**

Table 10b: Schedule of events from diagnosis to 96 days after challenge for volunteers in VAC069B *(timeline/window are in relation to the day of challenge). **D** = diagnosis**;** **C** = challenge; **T** = treatment; **(X)** = If considered necessary.*Physical observations include blood pressure, pulse and temperature. **Biochemistry comprises sodium, potassium, urea, creatinine, albumin, liver function tests. ^Diagnostic serology includes;* HBsAg, HCV antibodies, HIV antibodies, CMV antibodies and EBV

antibodies. *However, EBV and CMV serology will only be repeated post-challenge if the participant had negative serology results at screening.* [a] *Bloods to be taken immediately before drug treatment, if diagnostic criteria not met, treatment will be started at C+28 morning visit* [b] *If diagnosis is made at C+14.5 or C+21.5 visit, immunology draw will not be taken, since a large volume bleed is taken for immunological analysis at morning C+14 and C+21 visits.* [c] *Blood draws for microscopy and qPCR will continue until the volunteer has two consecutive qPCR readings with substantial reduction in genome copies per mL (and at least one negative thick film reading if treated on basis of thick film).*

Day	D/C+28 if undiagnosed[a]	T+1[c]	T+2 [c]	T+3	T+6	T+9	C+56	C+96
**Attendance number**	**43**	**44**	**45**	**46**	**47**	**-**	**48**	**49**
**Timeline (days) (window)**	**-**	**-**	**-**	**-**	**-**	**+2**	**56(±14)**	**96(±14)**
Physical observations*	X	X	X	X	X		X	X
Medical history/examination	(X)	(X)	(X)	(X)	(X)		(X)	(X)
Review AEs & medications	X	X	X	X	X		X	X
Review contraindications	X							
Phone call						X		
Diary card collected					X		(X)	(X)
Antimalarials Initiated	X							
Urine β-hCG (females only)	X							
Serum βhCG (mL)	5							
Haematology (mL)	2	2		2	2		2	2
Biochemistry (mL)**	3	3		3	3		3	3
Safety serum^								4
Exploratory Assays	106	12		12	12		82	70
DMFA (mL)	10							
Direct feeding assay (mosquito feeding)[@]	X							
qPCR & gametocyte PCR (mL)[C]	2	2	2					
SARS-COV-2 PCR swab	X							
**Visit Total Blood Volume (mL)**	**128**	**19**	**2**	**17**	**17**	**0**	**87**	**79**
**Cumulative blood volume (mL)**	**665**	**684**	**686**	**703**	**720**	**720**	**807**	**886**

Table 10c: Schedule of events from diagnosis to 96 days after challenge for volunteers in VAC069C *(timeline/window are in relation to the day of challenge or treatment). **D** = diagnosis**; C** = challenge; **T** = treatment; **(X)** = If considered necessary.*Physical observations include blood pressure, pulse and temperature. **Biochemistry comprises sodium, potassium, urea, creatinine, albumin, liver function tests. ^Diagnostic serology includes;* HBsAg, HCV antibodies, HIV antibodies, CMV antibodies and EBV antibodies. *However, EBV and CMV serology will only be repeated post-challenge if the participant had negative serology results at screening.* [a]*Bloods to be taken immediately before drug treatment, if diagnostic criteria not met, treatment will be started at C+28 morning visit* [b] *If diagnosis is made at C+14.5 or C+21.5 visit, immunology draw will not be taken, since a large volume bleed is taken for immunological analysis at morning C+14 and C+21 visits.* [c] *Blood draws for qPCR will continue daily until the volunteer has two consecutive qPCR readings with substantial reduction in genome copies per mL.* [@]*DFA is optional. Blood volumes as given in the table are maximal volumes and smaller blood volumes may be taken at any visit, at the discretion of the investigator, with the exception of additional blood volume that may be required for any additional tests that may be performed if deemed clinically necessary.*

Day	D/C+21 if undiagnosed[a]	T+1[c]	T+3[c]	T+6	T+9	C+56	C+96
**Attendance number**	**29**	**30**	**31**	**32**	**33**	**34**	**35**
**Timeline (days) (window)**	**-**	**-**	**-**	**±1**	**±2**	**56(±14)**	**96(±14)**
Physical observations*	X	X	X	X		X	X
Medical history/examination	(X)	(X)	(X)	(X)		(X)	(X)
Review AEs & medications	X	X	X	X		X	X
Review contraindications	X						
Phone call					X		
Diary card collected				X		(X)	(X)
Antimalarials Initiated	X						
Urine β-hCG (females only)	X						
Serum βhCG (mL)	5						
Haematology (mL)	2	2	2	2		2	2
Biochemistry (mL)**	3	3	3	3		3	3
Serum storage							4
HIV, HBV, HCV, EBV, CMV serology (mL)^							5
Exploratory Assays	106[b]	12	12	12		82	70
DMFA (mL)	10						
Direct feeding assay (mosquito feeding)@	X						
qPCR & gametocyte PCR (mL)[C]	2	2	2				
SARS-COV-2 PCR swab	X						
**Visit Total Blood Volume (mL)**	**128**	**19**	**19**	**17**	**0**	**87**	**84**
**Cumulative blood volume (mL)**	**507**	**526**	**545**	**562**	**562**	**649**	**733**

Table 10d: Schedule of events from diagnosis to 96 days after challenge for volunteers in VAC069D *(timeline/window are in relation to the day of challenge or treatment).* ***D*** *= diagnosis;* ***C*** *= challenge;* ***T*** *= treatment;* ***(X)*** *= If considered necessary.*Physical observations include blood pressure, pulse and temperature. **Biochemistry comprises sodium, potassium, urea, creatinine, albumin, liver function tests. ^EBV and CMV serology will only be repeated at the C+96 timepoint if they were negative at screening. [a]Bloods to be taken immediately before drug treatment, if diagnostic criteria not met, treatment will be started at C+21 morning visit [b] If diagnosis is made at C+14.5 visit, 73ml of immunology bleed will not be taken, since a large volume bleed is taken for immunological analysis at morning C+14 visit. [c] Blood draws for qPCR will continue until the volunteer has two consecutive qPCR readings with substantial reduction in genome copies per mL. @DFA is optional. Blood volumes as given in the table are maximal volumes and smaller blood volumes may be taken at any visit, at the discretion of the investigator, with the exception of additional blood volume that may be required for any additional tests that may be performed if deemed clinically necessary.*

Day	D/C+21 if undiagnosed[a]	T+1	T+3	T+6	T+10	C+56	C+96
**Attendance number**	**33**	**34**	**35**	**36**	**37**	**38**	**39**
**Timeline (days) (window)**	**-**	**-**	**(±0.5)**	**(±1)**	**(±2)**	**56 (±14)**	**96 (±14)**
Physical observations*	X	X	X	X	X	X	X
Medical history/examination	(X)	(X)	(X)	(X)	(X)	(X)	(X)
Review AEs & medications	X	X	X	X	X	X	X
Review contraindications	X						
Diary card collected				X			
Antimalarials Initiated	X						
Urine β-hCG (females only)	X						
Serum βhCG (mL)	5[b]						
Haematology (mL)	2[b]	2	2	2	2	2	6[d]
Biochemistry (mL)**	3[b]	3	3	3	3	3	3
Serum storage							4
HIV, HBV, HCV, EBV, CMV serology (mL)^							5
Exploratory Assays	72	12	12	12	12	72	42
qPCR & gametocyte PCR (mL)[C]	2	2	2				
**Visit Total Blood Volume (mL)**	**84**	**19**	**19**	**17**	**17**	**77**	**60**
**Cumulative blood volume (mL)**	**387**	**406**	**425**	**442**	**459**	**519**	**579**

Table 10e: Schedule of events for volunteers in VAC069E *(timeline/window are in relation to the day of challenge or treatment).* ***D*** *= diagnosis;* ***C*** *= challenge;* ***T*** *= treatment;* ***(X)*** *= If considered necessary.*Physical observations include blood pressure, pulse and temperature. **Biochemistry comprises sodium, potassium, urea, creatinine, liver function tests. ^EBV and CMV serology will only be repeated at the C+96 timepoint if they were negative at screening.* *[a]Bloods to be taken immediately before drug treatment, if diagnostic criteria not met, treatment will be started at C+21 morning visit. [b] If diagnosis is made at C+14.5 visit, haematology and biochemistry blood tests will not be repeated as already taken at morning C+14 visit. [c] Blood draws for qPCR will continue until the volunteer has two consecutive qPCR readings with substantial reduction in genome copies per mL. [d] 4ml EDTA will be sent for alloantibody screening at C+96. Blood volumes as given in the table are maximal volumes and smaller blood volumes may be taken at any visit, at the discretion of the investigator, with the exception of additional blood volume that may be required for any additional tests that may be performed if deemed clinically necessary.*

10 ASSESSMENT OF SCIENTIFIC OBJECTIVES

Statistical analysis will reference the following endpoints:

10.1 Primary endpoints

Assessment of:

- Safety and feasibility of primary *P. vivax* blood-stage CHMI, as measured by (S)AE occurrences and successful infection (development of detectable persistent parasitaemia by qPCR +/- clinical symptoms).
- Choosing the optimal inoculation dose to take forward to future *P. vivax* CHMI studies will be decided based on the following algorithm:
  Ideal choice = the first group (2/2 volunteers) to reach diagnosis criteria (within 21 days).
  N.B. If both volunteers in Group 1 develop infection AND both volunteers in Group 2 (or 3) reliably develop infection within 5 days of Group 1 (and within the 21-day window) then the lowest dose group should be chosen.

10.2 Secondary endpoints

- Safety and feasibility of secondary and tertiary *P. vivax* controlled blood-stage CHMI, as measured by (S)AE occurrences and successful infection (development of detectable persistent parasitaemia by qPCR +/- clinical symptoms)
- Immune response to primary, secondary and tertiary *P. vivax* and heterologous *P. falciparum* pre-treatment, as measured by antibody, B cell and T cell responses.
- Gametocytaemia, as measured by qPCR in primary, secondary and tertiary *P. vivax* blood-stage CHMI.
- Transmissibility of gametocytes from the infected volunteer to *Anopheline* mosquito vector

11 ASSESSMENT OF SAFETY

Safety of the CHMI will be assessed by analysing the frequency, incidence and nature of adverse events and serious adverse events arising during the study.

11.1 Definitions

Adverse Event (AE): An AE is any untoward medical occurrence in a volunteer, including a dosing error, which may occur during or after administration of a study intervention (in this case, CHMI) and does not necessarily have a causal relationship with the intervention. An AE can therefore be any unfavourable and unintended sign (including an abnormal laboratory finding), symptom or disease temporally associated with the study intervention, whether or not considered related to the study intervention.

Adverse Reaction (AR): An AR is any untoward or unintended response to a study intervention. This means that a causal relationship between the intervention and an AE is at least a reasonable possibility, i.e. the relationship cannot be ruled out. All cases judged by either the reporting medical Investigator or the Sponsor as having a reasonable suspected causal relationship to the intervention (i.e. possibly, probably or definitely related to an intervention) will qualify as adverse reactions.

Unexpected Adverse Reaction: An adverse reaction, the nature or severity of which is not consistent with CHMI, blood donation or antimalarial treatment (applicable product information) is considered as an unexpected adverse reaction.

Serious Adverse Event (SAE): An SAE is an AE that results in any of the following outcomes, whether or not considered related to the study intervention.

- Death (i.e. results in death from any cause at any time).
- Life-threatening event (i.e. the volunteer was, in the view of the Investigator, at immediate risk of death from the event that occurred). This does not include an AE that, if it occurred in a more serious form, might have caused death.
- Persistent or significant disability or incapacity (i.e. substantial disruption of one’s ability to carry out normal life functions).
- Transfer of inpatient care to the NHS staff if the Investigators assess that a higher level of intervention and intense monitoring is required to manage symptoms following CHMI, blood donation, or drugs (over and above what can be provided by the Investigators in the research bay). Hospitalisation (including inpatient or outpatient hospitalisation for an elective procedure) for a pre-existing condition that has not worsened unexpectedly does not constitute a SAE.
- An important medical event (that may not cause death, be life threatening, or require hospitalisation) that may, based upon appropriate medical judgment, jeopardise the volunteer and/or require medical or surgical intervention to prevent one of the outcomes listed above.
- Congenital anomaly or birth defect.

Serious Adverse Reaction (SAR): An adverse event (expected or unexpected) that is both serious and, in the opinion of the reporting Investigator or Sponsor, believed to be possibly, probably or definitely due to a study intervention or treatment, based on the information provided.

Suspected Unexpected Serious Adverse Reactions (SUSARs): A SUSAR is a SAE that is unexpected and thought to be possibly, probably or definitely related to a study intervention.

11.2 Causality assessment

For every unsolicited AE, an assessment of the relationship of the AE to the study intervention(s) (CHMI) will be undertaken by the CI-delegated clinician. The relationship of the adverse event with the study procedures will be categorised as unrelated, unlikely to be related, possibly related, probably related or definitely related (Table 11). An intervention-related AE refers to an AE for which there is a possible, probable or definite relationship to the study intervention. The delegated clinician will use clinical judgment to determine the relationship. Alternative causes of the AE, such as the natural history of pre-existing medical conditions, concomitant therapy, other risk factors and the temporal relationship of the event to CHMI will be considered and investigated. Causality assessment will take place during planned safety reviews, interim analyses and at the final safety analysis, except for SAEs, which should be assigned by the reporting Investigator.

0	No Relationship	No temporal relationship to study intervention ***and***<br>Alternate aetiology (clinical state, environmental or other interventions); ***and***<br>Does not follow known pattern of response to CHMI, blood donation or drug.
1	Unlikely	Unlikely temporal relationship to study intervention ***and***<br>Alternate aetiology likely (clinical state, environmental or other interventions) ***and***<br>Does not follow known typical or plausible pattern of response to CHMI, blood donation or drug.
2	Possible	Reasonable temporal relationship to study intervention; ***or***<br>Event not readily produced by clinical state, environmental or other interventions; ***or***<br>Similar pattern of response to that seen with previous CHMI, blood donation or similar drug.
3	Probable	Reasonable temporal relationship to study intervention; ***and***<br>Event not readily produced by clinical state, environment, or other interventions ***or***<br>Known pattern of response seen with previous CHMI, blood donation or drug.
4	Definite	Reasonable temporal relationship to study intervention; ***and***<br>Event not readily produced by clinical state, environment, or other interventions; ***and***<br>Known pattern of response seen with previous CHMI, blood donation or drug.

Table 11: Guidelines for assessing the relationship of study intervention to an AE

11.3 Reporting procedures for all AEs (see SOP VC027)

All AEs occurring post-CHMI, until 6 days after completion of antimalarial treatment (i.e. 9 days after DoD) will be recorded. We will NOT record bruising due to blood-taking or cannulation. Recording and reporting of all AEs will take place as detailed in SOP VC027. All AEs that result in a volunteer's withdrawal from the study will be followed up until a satisfactory resolution occurs, the event is considered stable, or until a non-study related causality is assigned (if the volunteer consents to this). Serious adverse events (SAEs) will be collected throughout the entire trial period.

Each adverse event will be graded by the participant according to the table for grading severity of adverse events (see Table 12). Severity gradings may be reviewed and discussed with the participants at the clinic visits. Volunteers will be instructed on how to self-assess the severity of these AEs. There will also be space on the diary card to self-document unsolicited AEs, and whether medication was taken to relieve the symptoms.

The severity of clinical adverse events will be assessed by the Investigators according to the scales in Tables 12-13.

GRADE 0	None
GRADE 1	Mild: Transient or mild discomfort (< 48 hours); no medical intervention/therapy required
GRADE 2	Moderate: Mild to moderate limitation in activity – some assistance may be needed; no or minimal medical intervention/therapy required
GRADE 3	Severe: Marked limitation in activity, some assistance usually required; may require medical intervention/therapy

Table 12: Severity grading criteria for AEs.

Physical Observations	Grade 1	Grade 2	Grade 3
Tachycardia – beats per min*	101-115	116-130	>130
Hypotension (systolic) mm Hg	85-89	80-84	<80
Hypertension (systolic) mm Hg**	141-159	160-179	>180
Hypertension (diastolic) mm Hg**	91-99	100-109	>110
Fever °C	37.6 – 38.0	>38.0	>39.0

Table 13: Severity grading criteria for clinically significant abnormal physical observations. All observations should be measured at rest. *Only applies when resting heart rate is between 60

and 100 beats per minute. Use clinical judgement when characterising bradycardia in some healthy subject populations (e.g. conditioned athletes). **Systolic or diastolic hypertension may only be confirmed as clinically significant (and therefore an AE) if persistently present when observations are repeated (i.e. isolated measurements of hypertension are not clinically significant).

11.3.1 Severity grading for laboratory AEs

Severity grading for laboratory AEs are dependent on the OUH laboratory's reference and will be graded according to the scales in the site-specific table that can be found in the Investigator file. These ranges will be based on FDA guidance relative to local laboratory reference ranges [82].

11.3.2 Reporting procedures for serious AEs

In order to comply with current regulations on serious adverse event reporting to regulatory authorities, the event will be documented accurately and notification deadlines respected. SAEs will be reported to an internal safety group (members of the study team) immediately (within 24 hours) of the Investigators being aware of their occurrence, as described in the SOP OVC005. This safety group includes the Chief Investigator, who acts on behalf of the Sponsor for notification of SAEs. The Sponsor and Local Safety Monitor (LSM) will be notified of SAEs deemed possibly, probably or definitely related to study interventions; the LSM will be notified immediately (within 24 hours) of the Investigators' being aware of their occurrence. SAEs will not normally be reported to the ethical committee(s) unless there is a clinically important increase in occurrence rate, an unexpected outcome, or a new event that is likely to affect safety of trial volunteers, at the discretion of the Chief Investigator and/or LSM.

11.3.4 Reporting procedures for SUSARs

Unexpected serious reactions would be reported to the Local Safety Monitor, ethical committees, Sponsor and other interested parties. For fatal and life-threatening SUSARS, this will be done within 7 calendar days after the Sponsor or delegate is first aware of the reaction. Any additional relevant information will be reported within 8 calendar days of the initial report. All other SUSARs will be reported within 15 calendar days. The Chief Investigator will also inform all investigators concerned of relevant information about SUSARs that could adversely affect the safety of participants.

All SUSARs and deaths occurring during the study will be reported to the Sponsor. For all deaths, available autopsy reports and relevant medical reports will be made available for reporting to the relevant authorities.

11.3.5 Procedures to be followed in the event of abnormal findings

Abnormal clinical findings from medical history, examination or blood tests, will be assessed as to their clinical significance using the site-specific laboratory adverse event grading table kept in the trial master file. If a test is deemed clinically significant, it may be repeated, to ensure it is not a single occurrence. If a test remains clinically significant, the volunteer will be informed and appropriate medical care arranged as appropriate with the permission of the volunteer. Decisions to exclude the volunteer from enrolling in the trial or to withdraw a volunteer from the trial will be at the discretion of the Investigator.

11.4 Foreseeable medical occurrences

The following medical occurrences are foreseeable:

- Clinical *P. vivax* or *P. falciparum* disease resulting in one or more of fever, tachycardia, hypotension, feverishness, chills, rigor, sweats, headache, anorexia, nausea, vomiting, myalgia, arthralgia, low back pain, fatigue, lymphopenia and thrombocytopenia;
- Severe clinical *P. vivax* or *P. falciparum* disease resulting in inpatient transfer of care to the NHS;
- Adverse reactions to Riamet®, Malarone, paracetamol or cyclizine, as detailed in the SmPCs for these medications.

11.5 Safety profile review of CHMI and antimalarial treatment

The safety profile will be assessed on an on-going basis by the Investigators.

11.6 Local safety monitor

An independent Local Safety monitor (LSM) will be appointed to provide real-time safety oversight. The LSM will review SAEs deemed possibly, probably or definitely related to study interventions. The LSM will be notified within 24 hours of the investigators' being aware of their occurrence. The LSM has the power to terminate the study if deemed necessary following a study intervention-related SAE. At the time of writing, the LSM will be Prof Brian Angus, a Clinical Tutor in Medicine, Honorary Consultant Physician and Director, Centre for Tropical Medicine at the University of Oxford. All correspondence between investigator and LSM will be conveyed by the investigator to the trial Sponsor.

The LSM may be contacted for advice and independent review by the investigator or trial Sponsor in the following situations:

- Following any SAE deemed to be possibly, probably, or definitely related to a study intervention.
- Any other situation where the Investigator or trial sponsor feels independent advice or review is important.

12 STATISTICS

12.1 Sample size

This is single site controlled human malaria infection study, aiming to assess the safety and feasibility of blood-stage controlled human *P. vivax* malaria infection using a newly produced cryopreserved inoculum source.

In the first phase of the study, VAC069A, the inoculum will be injected at three different concentrations, to assess the safety and feasibility of each, with the view to use the most appropriate concentration in the subsequent challenges. The number of volunteers undergoing malaria challenge in the first phase will be 6, with 2 volunteers undergoing CHMI at each concentration. The primary objective of the first phase is to assess safety, therefore, this number limits the potential for harm whilst allowing establishment of safety.

Subsequent phases of the study (VAC069B-D) will have larger numbers of volunteers (up to 8) per group, thus increasing the likelihood of detecting statistically significant differences in response to homologous rechallenge.

This is the first study of its kind to be performed in Europe, and if successful, future challenge studies to assess efficacy of candidate vaccines will be powered appropriately.

For VAC069E – 11 volunteers who underwent primary or secondary CHMI in the previous phase of the study (VAC069D) will be invited to continue in the study and undergo heterologous CHMI with *P. falciparum*. A minimum of 6 volunteers are likely to be required to detect significant differences in the clinical and immunological response to heterologous re-challenge.

13 QUALITY CONTROL AND QUALITY ASSURANCE PROCEDURES

13.1 Investigator procedures

Approved site-specific SOPs will be used at all clinical and laboratory sites.

13.2 Monitoring

Monitoring will be performed externally by RGEA using established procedures, according to ICH Good Clinical Practice (GCP). Following written standard operating procedures, the monitors will verify that the clinical trial is conducted and data are generated, documented and reported in compliance with the protocol and GCP. The Investigator sites will provide direct access to all trial related source data/documents and reports for the purpose of auditing by the Sponsor and inspection by local and regulatory authorities.

13.3 Modification to protocol

No amendments to this protocol will be made without consultation with, and agreement of, the Sponsor. Any amendments to the trial that appear necessary during the course of the trial must be discussed by the Investigator and Sponsor concurrently. If agreement is reached concerning the need for an amendment, it will be produced in writing by the Chief Investigator and will be made a formal part of the protocol following ethical approval.

An administrative change to the protocol is one that modifies administrative and logistical aspects of a protocol but does not affect the subjects' safety, the objectives of the trial and its progress. An administrative change does not require UK ethical committee approval; however, the Research Ethics Committee (REC) will be notified in the event of any such change.

The Investigator is responsible for ensuring that changes to an approved trial, during the period for which ethical committee(s) approval has already been given, are not initiated without ethical committee(s)' review and approval except to eliminate apparent immediate hazards to the subject.

13.4 Protocol deviation

Any deviations from the protocol will be documented in a protocol deviation form and filed in the site trial master file.

13.5 Audit & inspection

The QA manager performs system based internal audits to check that trials are being conducted, data recorded, analysed and accurately reported according to study protocols, departmental SOPs and in compliance with ICH GCP. The audit schedule includes laboratory activities. The internal audits will supplement the external monitoring process and will review processes not covered by the external monitor.

The Sponsor, trial sites, ethical committee(s), and authorised individuals may carry out audit to ensure compliance with the protocol, GCP and appropriate regulations. GCP inspections may also be undertaken by the regulatory authority to ensure compliance with protocol and national regulations. The Sponsor will assist in any inspections.

13.6 Serious breaches

A serious breach is defined as "A breach of GCP or the trial protocol which is likely to effect to a significant degree –

(a) the safety or physical or mental integrity of the subjects of the trial; or

(b) the scientific value of the trial.

In the event that a serious breach is suspected the Sponsor will be informed as soon as possible and in turn will notify the REC within 7 days.

13.7 Trial progress

The progress of the trial will be overseen by the Chief Investigator.

13.8 Publication policy

The Investigators will be involved in reviewing drafts of the manuscripts, abstracts, press releases and any other publications arising from the study. Authorship will be determined in accordance with the ICMJE guidelines and other contributors will be acknowledged. Data may also be used for a PhD or MD thesis.

13.9 Intellectual Property

Ownership of intellectual property (IP) generated by employees of the University vests in the University. The protection and exploitation of any new IP is managed by the University's technology transfer office, Oxford University Innovations

14 ETHICS

14.1 Declaration of Helsinki

The Investigator will ensure that this study is conducted according to the principles of the current revision of the Declaration of Helsinki 2008.

14.2 ICH guidelines for good clinical practice

The Investigator will ensure that this study is conducted in full conformity to Medicine for Human use (Clinical Trials) Regulations 2004 (SI 2004 No. 1031) and its amendments and with the ICH guidelines for GCP (CPMP/ICH/135/95) July 1996.

14.3 Approvals

A copy of the protocol, proposed informed consent form, other written volunteer information and the proposed advertising material will be submitted to an appropriate REC and the HRA for written approval. The Investigator will submit and, where necessary, obtain approval from the REC and the HRA for all subsequent amendments to the protocol and associated trial documents. A non-substantial amendment does not require REC approval; however, the REC will be notified in the event of any such change. The Investigator will notify deviations from the protocol or SAEs occurring at the site to the Sponsor and will notify the REC(s) of these if necessary in accordance with procedures.

14.4 Reporting

The CI shall submit once a year throughout the study, or on request, an Annual Progress Report to the REC, HRA (where required), host organisation and Sponsor. In addition, an End of Study notification and final report will be submitted to the REC and Sponsor.

14.5 Volunteer confidentiality

All data will be anonymised; volunteer data will be identified by a unique study number in the CRF and database. Separate confidential files containing identifiable information will be stored in secured locations. Only the Sponsor representative, Investigators, the clinical monitor, the ethical committee(s) and the regulatory authorities will have access to the records. Photographs taken (if required, with the volunteer’s written, informed consent) will not include the volunteer’s face and will be identified by the volunteer’s trial specific identification number only. Once developed, photographs will be stored as confidential records, as above. This material may be shown to other professional staff, used for educational purposes, or included in a scientific publication.

If a participant is diagnosed with COVID-19 on a test carried out through this study, personal details of the participant (name, date of birth, contact details, ethnicity, NHS number) and date of diagnosis and symptom onset will be legally required to be passed onto UKHSA as COVID-19 is a notifiable disease.

15 DATA HANDLING AND RECORD KEEPING

15.1 Data handling

The Chief Investigator will be the data manager with responsibility for delegating the receiving, entering, cleaning, querying, analysing and storing all data that accrues from the study. Data will be entered into the volunteers' CRFs in a paper and/or electronic format (using the OpenClinica™ database). Electronic data will be stored on secure servers which are outsourced by OpenClinica™. OpenClinica™ meets FDA part 11B standards. This includes safety data, laboratory data and outcome data. Data are entered in a web browser on PCs and then transferred to the OpenClinica Database by encrypted (https) transfer. Safety data will also be collected through an electronic diary, which is stored on a secure server.

15.2 Record keeping

The Investigators will maintain and retain appropriate medical and research records and essential documents for this trial in compliance with ICH E6 GCP and regulatory and institutional requirements for the protection of confidentiality of volunteers. The Chief Investigator, co-investigators, clinical research nurses and authorised personnel will have access to records. The investigators will permit authorised representatives of the Sponsor, ethical committee(s), regulatory agencies, and the monitors to examine (and when required by applicable law, to copy) clinical records for the purposes of quality assurance reviews, audits and evaluation of the study safety and progress.

15.3 Source data and electronic case report forms (eCRFs)

All protocol-required information will be collected in the electronic diaries and eCRFs designed by the Investigator. All source documents will be filed in the participants' notes. Source documents are original documents, data, and records from which the volunteer's eCRF data are obtained. For this study these will include, but are not limited to; volunteer consent form, blood results, GP response letters, laboratory records and correspondence. In the majority of cases, electronic diaries and eCRF entries will be considered source data as these are the site of the original recording (i.e. there is no other written or electronic record of data). In this study this will include, but is not limited to medical history, medication records, vital signs, physical examination records, urine assessments, blood results, adverse event data and details of study interventions. All source data and volunteer CRFs will be stored securely.

15.4 Data protection

The study protocol, documentation, data and all other information generated will be held in strict confidence. No information concerning the study or the data will be released to any unauthorised third party, without prior written approval of the Sponsor.

In the event of a participant testing positive for COVID-19 on a test carried out through this study, details of the participant (name, date of birth, contact details, ethnicity, NHS number) and date of diagnosis and symptom onset will be legally required to be passed onto UKHSA as COVID-19 is a notifiable disease.

16 FINANCING AND INSURANCE

16.1 Financing

The study is funded through a grant from The Wellcome Trust and MultiViVax, a European Commission Horizon 2020 funded project which intends to develop a highly effective multi-stage malaria vaccine to the point of proof-of-concept Phase II testing in Europe, prior to clinical trials in malaria-endemic areas.

16.2 Insurance

The University has a specialist insurance policy in place which would operate in the event of any participant suffering harm as a result of their involvement in the research (Newline Underwriting Management Ltd, at Lloyd's of London). NHS indemnity operates in respect of the clinical treatment that is provided.

16.3 Compensation

Volunteers will be compensated for their time and for the inconvenience caused by procedures as below (see Table 17).

- Screening visit	£25
Subsequent visits:	
- Travel expenses	£15 per visit
- Inconvenience of blood tests:	£10 per blood donation
- Time required for visit:	£20 per hour
- Admission (Illness) Compensation	£480 total
- Direct mosquito feeding (optional)	£80 per visit

The backup volunteers who are not enrolled in the study will be compensated £200. This is in addition to compensation for visits they may have attended. Volunteers partaking in direct mosquito bite feeding will also be compensated an additional £80 per visit.

	Time in Trial (approx.)	Maximum No. of Visits	Maximum Volume of Blood Taken (mL)	Estimated Compensation
**Vols enrolled in VAC069A (3 Challenges)**	30 months	148	2971	£6500
**Vols enrolled in VAC069B (3 Challenges)**	30 months	148	2868	£6715
**Vols enrolled in VAC069C (3 Challenges)**	18 months	147	2352	£6375
**Vols enrolled in VAC069D (3 Challenges)**	18 months	105	2199	£6240
**Vols enrolled in VAC069E (1 Challenge)**	3 months	39	579	£1970

Table 14: Estimated compensation amounts. Figures as given in table do not include compensation for direct mosquito bite feeding; any volunteer taking part in direct mosquito bite feeding will be compensated an additional £80 per visit.

17 APPENDICES

Appendix A: laboratory values for exclusion

Laboratory parameters for inclusion/exclusion in the trial will be considered on an individual basis, with Investigator discretion for interpretation of results and the need for repeated or further tests. In general, volunteers will be excluded if a result at screening constitutes what would qualify as a grade 1 (or higher) laboratory adverse event, according to the laboratory adverse event tables (filed in the TMF), on repeat of an abnormal test result. Urinalysis at screening will be assessed as per the table below:

URINE ANALYSIS (using MULTISTIX)	
Protein*	2+ or Protein creatinine ratio of ≥ 50 mg/mmol
Blood[£]	2+ on two dipstick tests
Glucose	1+

Table 15: Urinalysis assessment.

*In the event of the dipstick testing positive for protein with ≥1+ protein urine should be sent for a protein creatinine ratio.

[£] In the event of urine dipstick testing positive for ≥1+ blood with, or without, protein, a repeat dipstick test will be carried out to confirm haematuria. In female volunteers, a menstrual history will be taken to elicit whether the subject is currently menstruating and if they are, urine dipstick will be repeated after 1 - 2 weeks. If blood and/or proteinuria persist in any volunteer, an interpretation of the results will be undertaken by the Investigator on an individual basis to determine if they will be excluded from the trial, and the appropriate follow-up arranged.

18 REFERENCES

1. Baird, J.K., *Evidence and implications of mortality associated with acute Plasmodium vivax malaria.* Clin Microbiol Rev, 2013. **26**(1): p. 36-57.
2. Organisation, W.H. *World malaria report 2017*. 2017.
3. Rahimi, B.A., et al., *Severe vivax malaria: a systematic review and meta-analysis of clinical studies since 1900.* Malar J, 2014. **13**: p. 481.
4. Rogerson, S.J., et al., *Burden, pathology, and costs of malaria in pregnancy: new developments for an old problem.* Lancet Infect Dis, 2018. **18**(4): p. e107-e118.
5. Price, R.N., et al., *Vivax malaria: neglected and not benign.* Am J Trop Med Hyg, 2007. **77**(6 Suppl): p. 79-87.
6. Carlton, J.M., B.J. Sina, and J.H. Adams, *Why is Plasmodium vivax a neglected tropical disease?* PLoS Negl Trop Dis. **5**(6): p. e1160.
7. Watson, J., et al., *Implications of current therapeutic restrictions for primaquine and tafenoquine in the radical cure of vivax malaria.* PLoS Negl Trop Dis, 2018. **12**(4): p. e0006440.
8. Pybus, B.S., et al., *The metabolism of primaquine to its active metabolite is dependent on CYP 2D6.* Malar J, 2013. **12**: p. 212.
9. Bennett, J.W., et al., *Primaquine failure and cytochrome P-450 2D6 in Plasmodium vivax malaria.* N Engl J Med, 2013. **369**(14): p. 1381-2.
10. Sattabongkot, J., et al., *Plasmodium vivax transmission: chances for control?* Trends Parasitol, 2004. **20**(4): p. 192-8.
11. Grabowsky, M., *The billion-dollar malaria moment.* Nature, 2008. **451**(7182): p. 1051-2.
12. Moorthy, V.S., R.D. Newman, and J.M. Okwo-Bele, *Malaria vaccine technology roadmap.* Lancet, 2013. **382**(9906): p. 1700-1.
13. Mueller, I., A.R. Shakri, and C.E. Chitnis, *Development of vaccines for Plasmodium vivax malaria.* Vaccine, 2015. **33**(52): p. 7489-95.
14. Payne, R.O., et al., *Plasmodium vivax Controlled Human Malaria Infection - Progress and Prospects.* Trends Parasitol, 2016.
15. Novy, R.E., et al., *In vitro functional characterization of bacterially expressed human fibroblast tropomyosin isoforms and their chimeric mutants.* Cell Motil Cytoskeleton, 1993. **26**(3): p. 248-61.
16. Russell, B., et al., *A reliable ex vivo invasion assay of human reticulocytes by Plasmodium vivax.* Blood, 2011. **118**(13): p. e74-81.
17. March, S., et al., *A microscale human liver platform that supports the hepatic stages of Plasmodium falciparum and vivax.* Cell Host Microbe, 2013. **14**(1): p. 104-15.
18. Dembele, L., et al., *Persistence and activation of malaria hypnozoites in long-term primary hepatocyte cultures.* Nat Med, 2014. **20**(3): p. 307-12.
19. Mikolajczak, S.A., et al., *Plasmodium vivax liver stage development and hypnozoite persistence in human liver-chimeric mice.* Cell Host Microbe, 2015. **17**(4): p. 526-35.
20. Herrera, S., et al., *Successful sporozoite challenge model in human volunteers with Plasmodium vivax strain derived from human donors.* Am J Trop Med Hyg, 2009. **81**(5): p. 740-6.
21. Herrera, S., et al., *Consistent safety and infectivity in sporozoite challenge model of Plasmodium vivax in malaria-naive human volunteers.* Am J Trop Med Hyg, 2011. **84**(2 Suppl): p. 4-11.

22. Payne, R.O., et al., *Demonstration of the Blood-Stage Plasmodium falciparum Controlled Human Malaria Infection Model to Assess Efficacy of the P. falciparum Apical Membrane Antigen 1 Vaccine, FMP2.1/AS01.* J Infect Dis, 2016. **213**(11): p. 1743-51.

23. Lin, E., et al., *Differential patterns of infection and disease with P. falciparum and P. vivax in young Papua New Guinean children.* PLoS One, 2010. **5**(2): p. e9047.

24. Michon, P., et al., *The risk of malarial infections and disease in Papua New Guinean children.* Am J Trop Med Hyg, 2007. **76**(6): p. 997-1008.

25. Collins, W.E., G.M. Jeffery, and J.M. Roberts, *A retrospective examination of reinfection of humans with Plasmodium vivax.* Am J Trop Med Hyg, 2004. **70**(6): p. 642-4.

26. Pombo, D.J., et al., *Immunity to malaria after administration of ultra-low doses of red cells infected with Plasmodium falciparum.* Lancet, 2002. **360**(9333): p. 610-7.

27. Moxon, R., *Microbial Challenge Studies of Human Volunteers.* The Academy of Medical Sciences, 2005.

28. Pollard, A., Zambon, Maria, *Controlled Human Infection Model Studies.* The Academy of Medical Sciences, 2018.

29. Sauerwein, R.W., M. Roestenberg, and V.S. Moorthy, *Experimental human challenge infections can accelerate clinical malaria vaccine development.* Nat Rev Immunol, 2011. **11**(1): p. 57-64.

30. Trager, W. and J.B. Jensen, *Human malaria parasites in continuous culture.* Science, 1976. **193**(4254): p. 673-5.

31. Ifediba, T. and J.P. Vanderberg, *Complete in vitro maturation of Plasmodium falciparum gametocytes.* Nature, 1981. **294**(5839): p. 364-366.

32. Campbell, C.C., et al., *Plasmodium falciparum gametocytes from culture in vitro develop to sporozoites that are infectious to primates.* Science, 1982. **217**(4564): p. 1048-1050.

33. Chulay, J.D., et al., *Malaria transmitted to humans by mosquitoes infected from cultured Plasmodium falciparum.* Am J Trop Med Hyg, 1986. **35**(1): p. 66-8.

34. Herrington, D.A., et al., *Safety and immunogenicity in man of a synthetic peptide malaria vaccine against Plasmodium falciparum sporozoites.* Nature, 1987. **328**(6127): p. 257-9.

35. Ballou, W.R., et al., *Safety and efficacy of a recombinant DNA Plasmodium falciparum sporozoite vaccine.* Lancet, 1987. **1**(8545): p. 1277-81.

36. Moorthy, V., *Standardization of Design and Conduct of P. falciparum Sporozoite Challenge Trials.* 2011.

37. Payne, R.O., et al., *Plasmodium vivax Controlled Human Malaria Infection - Progress and Prospects.* Trends Parasitol, 2017. **33**(2): p. 141-150.

38. Whitrow, M., *Wagner-Jauregg and fever therapy.* Medical History, 1990. **34**: p. 294-310.

39. Snounou, G. and J.L. Perignon, *Malariotherapy--insanity at the service of malariology.* Adv Parasitol, 2013. **81**: p. 223-55.

40. Alving, A.S., et al., *PROCEDURES USED AT STATEVILLE PENITENTIARY FOR THE TESTING OF POTENTIAL ANTIMALARIAL AGENTS.* Journal of Clinical Investigation, 1948. **27**(3 Pt 2): p. 2-5.

41. Miller, L.H., et al., *The resistance factor to Plasmodium vivax in blacks. The Duffy-blood-group genotype, FyFy.* N Engl J Med, 1976. **295**(6): p. 302-4.

42. Rieckmann, K.H., et al., *Current considerations in vaccination of humans against malaria: Use of attenuated sporozoites in the immunization of human volunteers against falciparum malaria.* Bulletin of the World Health Organization, 1979. **57**(Suppl): p. 261-265.

43. Arevalo-Herrera, M., et al., *Plasmodium vivax sporozoite challenge in malaria-naive and semi-immune Colombian volunteers.* PLoS One, 2014. **9**(6): p. e99754.

44. Arevalo-Herrera, M., et al., *Protective Efficacy of Plasmodium vivax Radiation-Attenuated Sporozoites in Colombian Volunteers: A Randomized Controlled Trial.* PLoS Negl Trop Dis, 2016. **10**(10): p. e0005070.

45. Bennett, J.W., et al., *Phase 1/2a Trial of Plasmodium vivax Malaria Vaccine Candidate VMP001/AS01B in Malaria-Naive Adults: Safety, Immunogenicity, and Efficacy.* PLoS Negl Trop Dis, 2016. **10**(2): p. e0004423.

46. Vanloubbeeck, Y., et al., *Comparison of the immune responses induced by soluble and particulate Plasmodium vivax circumsporozoite vaccine candidates formulated in AS01 in rhesus macaques.* Vaccine, 2013. **31**(52): p. 6216-24.

47. McCarthy, J.S., et al., *Experimentally induced blood-stage Plasmodium vivax infection in healthy volunteers.* J Infect Dis, 2013. **208**(10): p. 1688-94.

48. Griffin, P., et al., *Safety and Reproducibility of a Clinical Trial System Using Induced Blood Stage Plasmodium vivax Infection and Its Potential as a Model to Evaluate Malaria Transmission.* PLoS Negl Trop Dis, 2016. **10**(12): p. e0005139.

49. Dunachie, S.J., et al., *A clinical trial of prime-boost immunisation with the candidate malaria vaccines RTS,S/AS02A and MVA-CS.* Vaccine, 2006. **24**(15): p. 2850-9.

50. Rampling, T., et al., *Safety and High Level Efficacy of the Combination Malaria Vaccine Regimen of RTS,S/AS01B with ChAd-MVA Vectored Vaccines Expressing ME-TRAP.* J Infect Dis, 2016.

51. Ewer, K.J., et al., *Protective CD8(+) T-cell immunity to human malaria induced by chimpanzee adenovirus-MVA immunisation.* Nat Commun, 2013. **4**: p. 2836.

52. Roestenberg, M., et al., *Long-term protection against malaria after experimental sporozoite inoculation: an open-label follow-up study.* Lancet, 2011. **377**(9779): p. 1770-6.

53. Cervia, J.S., B. Wenz, and G.A. Ortolano, *Leukocyte reduction's role in the attenuation of infection risks among transfusion recipients.* Clin Infect Dis, 2007. **45**(8): p. 1008-13.

54. Cheng, Q., et al., *Measurement of Plasmodium falciparum growth rates in vivo: a test of malaria vaccines.* Am J Trop Med Hyg, 1997. **57**(9347970): p. 495-500.

55. Engwerda, C.R., et al., *Experimentally induced blood stage malaria infection as a tool for clinical research.* Trends Parasitol, 2012. **28**(11): p. 515-21.

56. Bijker, E.M., et al., *Protection against malaria after immunization by chloroquine prophylaxis and sporozoites is mediated by preerythrocytic immunity.* Proc Natl Acad Sci U S A, 2013. **110**(19): p. 7862-7.

57. Sanderson, F., et al., *Blood-stage challenge for malaria vaccine efficacy trials: a pilot study with discussion of safety and potential value.* Am J Trop Med Hyg, 2008. **78**(6): p. 878-83.

58. Duncan, C.J., et al., *Impact on malaria parasite multiplication rates in infected volunteers of the protein-in-adjuvant vaccine AMA1-C1/Alhydrogel+CPG 7909.* PLoS One, 2011. **6**(7): p. e22271.

59. Minassian, A.M., et al., *Reduced blood-stage malaria growth and immune correlates in humans following RH5 vaccination.* Med (N Y), 2021. **2**(6): p. 701-719.e19.

60. Epstein, J.E., et al., *Safety and clinical outcome of experimental challenge of human volunteers with Plasmodium falciparum-infected mosquitoes: an update.* J Infect Dis, 2007. **196**(1): p. 145-54.

61. Verhage, D.F., et al., *Clinical outcome of experimental human malaria induced by Plasmodium falciparum-infected mosquitoes.* Neth J Med, 2005. **63**(2): p. 52-8.

62. Church, L.W., et al., *Clinical manifestations of Plasmodium falciparum malaria experimentally induced by mosquito challenge.* J Infect Dis, 1997. **175**(4): p. 915-20.

63. Nieman, A.E., et al., *Cardiac complication after experimental human malaria infection: a case report.* Malar J, 2009. **8**: p. 277.

64. van Meer, M.P., et al., *Idiopathic acute myocarditis during treatment for controlled human malaria infection: a case report.* Malar J, 2014. **13**: p. 38.

65. Roestenberg, M., et al., *Comparison of clinical and parasitological data from controlled human malaria infection trials.* PLoS One, 2012. **7**(6): p. e38434.

66. Sheehy, S.H., et al., *ChAd63-MVA-vectored blood-stage malaria vaccines targeting MSP1 and AMA1: assessment of efficacy against mosquito bite challenge in humans.* Mol Ther, 2012. **20**(12): p. 2355-68.

67. Statistics, O.o.N. *Coronavirus (COVID-19) Infection Survey pilot: England, 17 July 2020*. 2020 [cited 2020 23/07/2020]; Available from: https://www.ons.gov.uk/peoplepopulationandcommunity/healthandsocialcare/conditionsanddiseases/bulletins/coronaviruscovid19infectionsurveypilot/latest#incidence-rate.

68. Lam, S., A. Lombardi, and A. Ouanounou, *COVID-19: A review of the proposed pharmacological treatments.* Eur J Pharmacol, 2020: p. 173451.

69. Singh, K., et al., *Malaria vaccine candidate based on Duffy-binding protein elicits strain transcending functional antibodies in a Phase I trial.* NPJ Vaccines, 2018. **3**: p. 48.

70. Venkatraman, N., et al., *Safety and immunogenicity of heterologous prime-boost immunization with viral-vectored malaria vaccines adjuvanted with Matrix-M.* Vaccine, 2017. **35**(45): p. 6208-6217.

71. Di Angelantonio, E., et al., *Efficiency and safety of varying the frequency of whole blood donation (INTERVAL): a randomised trial of 45 000 donors.* Lancet, 2017. **390**(10110): p. 2360-2371.

72. *Position Statement, Creutzfeldt-Jakob Disease.* vCJD Working Party of the Standing Advisory Committee on Transfusion Transmitted Infections, UK Blood Services Joint Professional Advisory Group, 2015.

73. Institute, Q.B.M.R., *Investigator's Brochure for Blood stage Plasmodium falciparum challenge agent P. falciparum 3D7 (BSPC)*. 2021.

74. Roestenberg, M., et al., *Efficacy of preerythrocytic and blood-stage malaria vaccines can be assessed in small sporozoite challenge trials in human volunteers.* J Infect Dis, 2012. **206**(3): p. 319-23.

75. van Agtmael, M.A., et al., *The effect of grapefruit juice on the time-dependent decline of artemether plasma levels in healthy subjects.* Clin Pharmacol Ther, 1999. **66**(4): p. 408-14.

76. Robinson, S., et al., *The administration of blood components: a British Society for Haematology Guideline.* Transfus Med, 2018. **28**(1): p. 3-21.

77. Murphy, S.C., et al., *External quality assurance of malaria nucleic acid testing for clinical trials and eradication surveillance.* PLoS One, 2014. **9**(5): p. e97398.

78. Kamau, E., et al., *Measurement of parasitological data by quantitative real-time PCR from controlled human malaria infection trials at the Walter Reed Army Institute of Research.* Malar J, 2014. **13**: p. 288.

79. Friedman-Klabanoff, D.J., et al., *The Controlled Human Malaria Infection Experience at the University of Maryland.* Am J Trop Med Hyg, 2019. **100**(3): p. 556-565.

80. Seilie, A.M., et al., *Beyond Blood Smears: Qualification of Plasmodium 18S rRNA as a Biomarker for Controlled Human Malaria Infections.* Am J Trop Med Hyg, 2019. **100**(6): p. 1466-1476.

81. Ishizuka, A.S., et al., *Protection against malaria at 1 year and immune correlates following PfSPZ vaccination.* Nat Med, 2016. **22**(6): p. 614-23.

82. FDA, *Toxicity Grading Scale for Healthy Adult & Adolescent Volunteers enrolled in Preventative Vaccine Clinical Trials.* 2007.

Statistical Analysis Plan

VAC069

Trial title: A clinical study to assess the safety and feasibility of controlled blood-stage *Plasmodium vivax* human malaria infection through experimental inoculation of cryopreserved infected erythrocytes in healthy malaria-naïve UK adults

Trial registration number: NCT03797989

Protocol Version: 8.0

Protocol Date: 23/06/2022

Chief Investigator: Dr Angela M Minassian

Introduction

Background and rationale

Currently available methods for prevention and treatment of *Plasmodium vivax* malaria are inadequate. Development of a blood-stage controlled human malaria infection (CHMI) model would allow the study of the biology, pathogenesis and transmission of *P. vivax* and could be used to test the efficacy of new candidate vaccines. The primary aim of the study is to establish and assess the safety of the blood-stage *P. vivax* CHMI with a new *P. vivax* infected blood bank, which can then be used in future trials. The secondary aim is to study immunity and parasite growth dynamics during repeat homologous *P. vivax* CHMI as well as during repeat heterologous CHMI with *P. falciparum*.

Objectives

Primary Objectives

- To assess the safety and feasibility of controlled blood-stage *P. vivax* human malaria infection in healthy adult volunteers, through experimental injection of cryopreserved *P. vivax* infected erythrocytes
- To choose, based on the first phase of the study (VAC069A), the optimal inoculation dose of *P. vivax* for use in blood-stage CHMI studies.

Secondary Objectives

- To assess safety and feasibility of secondary and tertiary homologous blood-stage controlled human *P. vivax* malaria infection.
- To assess the immune response to primary, secondary and tertiary homologous and heterologous Plasmodium infection, through experimental injection of *P. vivax* and *P. falciparum*-infected erythrocytes.
- To assess gametocytaemia following primary, secondary and tertiary *P. vivax* infection through experimental injection of *P. vivax* infected erythrocytes.
- To assess transmission of *P. vivax* gametocytes from infected volunteers to mosquito vectors

Study Methods

Trial design

This is a CHMI study, consisting of 5 parts VAC069A to E, with each part corresponding to a CHMI when participants undergo primary and repeat CHMI in parallel. Participants undergo up to three blood-stage CHMI with *P. vivax* over subsequent parts of the study. In the final part of the study, heterologous repeat CHMI will be undertaken with *P. falciparum*.

Sample size

A total of up to 24 participants will undergo primary CHMI. A total of 6 participants will undergo primary CHMI during VAC069A to determine the optimal dose of inoculation to use for subsequent CHMIs. In VAC069B to D, between 2 to 8 participants will undergo primary CHMI. Participants who completed primary CHMI will undergo secondary and tertiary CHMI.

Timing of analyses

Interim immunogenicity and parasite growth analyses will be conducted at the end of each CHMI. For the final analysis, pooled data for participants completing primary CHMI will be compared to secondary and tertiary CHMI. Outcomes are measured to 3 months after each CHMI.

Confidence intervals and P values

A p value of <0.05 will be considered statistically significant. If analysis groups are of a small size (n < 6), formal statistical comparisons will not be conducted and descriptive analyses only will be presented. Group medians and interquartile ranges will be reported for numerical variables.

Missing data

No imputation for missing data will be conducted. Missing data will be treated as random.

Trial population

Malaria naïve, healthy, Duffy blood group positive UK adults aged 18-50 years will be enrolled. Participants will be analysed the number and type (*P. vivax* or *P. falciparum*) CHMI that they underwent. Data from participants who withdraw from the study will be included up to the date that they withdraw from the study. Baseline characteristics collected at screening comprise of age, gender, ethnicity, weight, height and Duffy phenotype.

Analysis

Analysis of Primary Objectives

To assess the safety and feasibility of controlled blood-stage *P. vivax* human malaria infection in healthy adult volunteers, through experimental injection of cryopreserved *P. vivax* infected erythrocytes

Safety will be assessed through analysis of solicited and unsolicited symptoms and signs, serious adverse events and laboratory blood abnormalities following each CHMI. Safety data for all participants undergoing primary CHMI will be pooled. Solicited and unsolicited adverse events will be graded for severity (grade 0 no event, grade 1 mild, grade 2 moderate, grade 3 severe). Unsolicited adverse events will be assessed for causality as unrelated, unlikely, possibly, probably or definitely related to CHMI. Laboratory (haematological and biochemical) abnormalities will be graded for severity (grade 1-3). The count and percentage of adverse events will be presented using GraphPad Prism. Feasibility of CHMI will be determined as successful infection of participants as determined by development of detectable parasitaemia.

To choose, based on the first phase of the study (VAC069A), the optimal inoculation dose of *P. vivax* for use in blood-stage CHMI studies.

VAC069A will enrol 6 participants, 2 will receive one vial's worth of inoculum each, 2 will receive one fifth and 2 will receive one twentieth of the dose. The lowest concentration of inoculum that reliably infects both participants will be chosen to be used in subsequent parts of the study (VAC069B to D).

Analysis of Secondary Objectives

To assess safety and feasibility of secondary and tertiary homologous blood-stage controlled human *P. vivax* malaria infection.

Safety during secondary and tertiary homologous CHMI will be assessed from the same outcomes as for primary CHMI but using pooled data from all participants undergoing secondary CHMI and pooled data from tertiary CHMI. Count and percentage of adverse events will be presented. For solicited and unsolicited adverse events no formal hypothesis testing or comparisons between

primary and secondary or tertiary CHMI will be performed. Pairwise comparisons of temperature and haematological and biochemical laboratory measures between groups will be made using Wilcoxon signed rank test. Comparisons between two groups of laboratory measures over multiple timepoints will be performed using linear regression to fit mixed effects models that include timepoint and CHMI number as categorical fixed effects and participant as a random effect using R. Linear hypothesis testing will be performed using multcomp's glht function with Benjamini-Hochberg correction for multiple testing.

Feasibility of repeat CHMI will be determined as successful infection of participants as determined by development of detectable parasitaemia.

To assess the immune response to primary, secondary and tertiary homologous and heterologous Plasmodium infection, through experimental injection of *P. vivax* and *P. falciparum*-infected erythrocytes.

Antibody, B cell and/or T cell responses to blood-stage *P. vivax* antigens will be measured at baseline (pre-CHMI), during CHMI and up to 3 months after each CHMI. Data from the following participants will be pooled for assessment: all primary *P. vivax* CHMI, all secondary *P. vivax* CHMI, all tertiary *P. vivax* CHMI and all *P. falciparum* heterologous CHMI. The effect of any anti-parasite immunity on parasite growth dynamics will be assessed by measurement of qPCR during CHMI. Descriptive graphs showing the qPCR over time for each participant and the medians for primary, secondary and tertiary CHMI will be presented. Parasite multiplication rate may be calculated for each individual using a linear model fitted to $\log_{10}$ transformed qPCR data. Formal comparisons between groups may not be performed if the group sizes are small. If performed, comparisons between groups will be made using non-parametric tests such as Wilcoxon signed rank test.

To assess gametocytaemia following primary, secondary and tertiary *P. vivax* infection through experimental injection of *P. vivax* infected erythrocytes.

Gametocytaemia will be measured by qPCR following each CHMI until commencement of antimalarial treatment. Comparison of gametocytaemia will be made between pooled data from participants undergoing primary CHMI and pooled data from secondary CHMI using Wilcoxon signed rank test.

To assess transmission of *P. vivax* gametocytes from infected volunteers to mosquito vectors

The transmissibility of gametocytes from infected volunteers to Anopheline mosquitos will be assessed by direct membrane feeding assay. The proportion of mosquitos infected will be reported.
