# Supplementary material for: Development of clinical immunity to Plasmodium vivax following repeat controlled human malaria infection
Source: Nat Commun. 2025 Sep 25;16:8385. doi: 10.1038/s41467-025-63104-y (PMC12462441; doi:10.1038/s41467-025-63104-y)
Supplement: Supplementary file 1 [file 41467_2025_63104_MOESM3_ESM.pdf]

nature portfolio

Corresponding author(s): Angela Minassian, Philip Spence

Last updated by author(s): Jul 23, 2025

Reporting Summary

Nature Portfolio wishes to improve the reproducibility of the work that we publish. This form provides structure for consistency and transparency in reporting. For further information on Nature Portfolio policies, see our Editorial Policies and the Editorial Policy Checklist.

Statistics

For all statistical analyses, confirm that the following items are present in the figure legend, table legend, main text, or Methods section.

n/a	Confirmed	
☐	☒	The exact sample size (*n*) for each experimental group/condition, given as a discrete number and unit of measurement
☐	☒	A statement on whether measurements were taken from distinct samples or whether the same sample was measured repeatedly
☐	☒	The statistical test(s) used AND whether they are one- or two-sided<br>*Only common tests should be described solely by name; describe more complex techniques in the Methods section.*
☐	☒	A description of all covariates tested
☐	☒	A description of any assumptions or corrections, such as tests of normality and adjustment for multiple comparisons
☐	☒	A full description of the statistical parameters including central tendency (e.g. means) or other basic estimates (e.g. regression coefficient) AND variation (e.g. standard deviation) or associated estimates of uncertainty (e.g. confidence intervals)
☐	☒	For null hypothesis testing, the test statistic (e.g. *F*, *t*, *r*) with confidence intervals, effect sizes, degrees of freedom and *P* value noted<br>*Give P values as exact values whenever suitable.*
☒	☐	For Bayesian analysis, information on the choice of priors and Markov chain Monte Carlo settings
☒	☐	For hierarchical and complex designs, identification of the appropriate level for tests and full reporting of outcomes
☒	☐	Estimates of effect sizes (e.g. Cohen's *d*, Pearson's *r*), indicating how they were calculated

Our web collection on statistics for biologists contains articles on many of the points above.

Software and code

Policy information about availability of computer code

Data collection	Clinical data collection with OpenClinica 3.
Data analysis	GraphPad Prism (v9.5.0). R (v4.4.2), using lme4 and stats packages.

For manuscripts utilizing custom algorithms or software that are central to the research but not yet described in published literature, software must be made available to editors and reviewers. We strongly encourage code deposition in a community repository (e.g. GitHub). See the Nature Portfolio guidelines for submitting code & software for further information.

Data

Policy information about availability of data

All manuscripts must include a data availability statement. This statement should provide the following information, where applicable:

- Accession codes, unique identifiers, or web links for publicly available datasets
- A description of any restrictions on data availability
- For clinical datasets or third party data, please ensure that the statement adheres to our policy

Individual de-identified participant information of all data presented in the main manuscript is now openly accessible (doi.org/10.5281/zenodo.15927920).

Research involving human participants, their data, or biological material

Policy information about studies with human participants or human data. See also policy information about sex, gender (identity/presentation), and sexual orientation and race, ethnicity and racism.

Reporting on sex and gender	Self-reported gender was collected at study screening visits. Both genders were enrolled in the study and study findings apply to both genders. Baseline characteristics including gender of participants is reported. No analysis by gender was conducted due to small sample sizes.
Reporting on race, ethnicity, or other socially relevant groupings	Self-reported ethnicity was collected at study screening visits. Categories where: White British, White other, Arab, Asian or Asian British and Mixed. The main reason for collection of ethnicity data was to determine if participants recruitment was representative of the local population. Ethnicity is reported in the table of participant baseline characteristics. Parasite growth grouped by self-reported ethnicity is presented in Fig.S2.
Population characteristics	Age was collected at study screening visits. No analysis by age was conducted.
Recruitment	Recruitment was through ethically approved advertisement distributed in public places, via clinical trials group website, email, social media accounts of the clinical trials group, at exhibition or fairs. Only participants who were geographically within a certain distance from the study site were recruited.
Ethics oversight	UK National Health Service Research Ethics Services, Hampshire A Research Ethics Committee, Ref 18/SC/0577 for VAC069 study. (Oxford A Research Ethics Committee, Ref 19/SC/0330 for VAC079 study.)

Note that full information on the approval of the study protocol must also be provided in the manuscript.

Field-specific reporting

Please select the one below that is the best fit for your research. If you are not sure, read the appropriate sections before making your selection.

☒ Life sciences ☐ Behavioural & social sciences ☐ Ecological, evolutionary & environmental sciences

For a reference copy of the document with all sections, see nature.com/documents/nr-reporting-summary-flat.pdf

Life sciences study design

All studies must disclose on these points even when the disclosure is negative.

Sample size	Sample size for the first part of the study was chosen to assess the feasibility and safety of CHMI whilst minimising harm. Subsequent parts of the study enrolled up to 8 participants per group. This sample size was chosen based on balancing larger sample size to detect a difference between primary and repeat CHMI and the limit on the number of participants able to undertake CHMI in parallel at the study centre at one time.
Data exclusions	No data were excluded.
Replication	The study was conducted in 5 parts with a CHMI conducted in each part. Reproducibility of findings was confirmed at each CHMI.
Randomization	No randomisation was performed. Participants were enrolled to undergo primary CHMI and subsequently underwent repeat CHMI. No correction for confounding variables was undertaken because of the small sample size.
Blinding	All participants underwent CHMI and previous number of CHMIs completed by each participant was known. Blinding to group allocation was not possible.

Reporting for specific materials, systems and methods

We require information from authors about some types of materials, experimental systems and methods used in many studies. Here, indicate whether each material, system or method listed is relevant to your study. If you are not sure if a list item applies to your research, read the appropriate section before selecting a response.

Materials & experimental systems

n/a	Involved in the study
☐	☒ Antibodies
☒	☐ Eukaryotic cell lines
☒	☐ Palaeontology and archaeology
☒	☐ Animals and other organisms
☐	☒ Clinical data
☒	☐ Dual use research of concern
☒	☐ Plants

Methods

n/a	Involved in the study
☒	☐ ChIP-seq
☒	☐ Flow cytometry
☒	☐ MRI-based neuroimaging

Antibodies

Antibodies used	PE-conjugated Anti-His Ab (R&D system, IC050P). Goat anti-human IgG secondary antibody (Thermo, PA1-86078). Goat anti-human IgG-alkaline phosphatase secondary antibody (Merck, A9544)
Validation	Commercial antibodies were validated by suppliers.

Clinical data

Policy information about clinical studies
All manuscripts should comply with the ICMJEguidelines for publication of clinical research and a completedCONSORT checklist must be included with all submissions.

Clinical trial registration: VAC069 NCT03797989. (VAC079 NCT04201431)

Study protocol: Protocols are available at clinicaltrials.gov

Data collection: The study was conducted at CCVTM, University of Oxford, UK. Recruitment commenced in November 2018. The last study visit occurred in December 2022.

Outcomes:

Primary objectives:
To assess the safety and feasibility of controlled blood-stage P. vivax human malaria infection in healthy adult volunteers, through experimental injection of cryopreserved P. vivax infected erythrocytes.
Safety was assessed through analysis of solicited and unsolicited symptoms and signs, serious adverse events and laboratory blood abnormalities following each CHMI. Safety data for all participants undergoing primary CHMI were pooled. Adverse events were graded for severity. The count and percentage of adverse events were presented using GraphPad Prism. Feasibility of CHMI was determined as successful infection of participants as determined by development of detectable parasitaemia.
To choose, based on the first phase of the study (VAC069A), the optimal inoculation dose of P. vivax for use in blood-stage CHMI studies.
This part of the study and associated objectives and outcomes has been published previously (PMID: 34609964).
Secondary objectives:
To assess safety and feasibility of secondary and tertiary homologous blood-stage controlled human P. vivax malaria infection.
Safety during secondary and tertiary homologous CHMI was assessed from the same outcomes as for primary CHMI but using pooled data from all participants undergoing secondary CHMI and pooled data from tertiary CHMI. Count and percentage of adverse events were presented. For solicited and unsolicited adverse events no formal hypothesis testing or comparisons between primary and secondary or tertiary CHMI were performed. Pairwise comparisons of temperature and haematological and biochemical laboratory measures between groups were made using Wilcoxon signed rank test. Comparisons between two groups of laboratory measures over multiple timepoints were performed using linear regression to fit mixed effects models that include timepoint and CHMI number as categorical fixed effects and participant as a random effect using R. Linear hypothesis testing was performed using multcomp's glht function with Benjamini-Hochberg correction for multiple testing. Feasibility of repeat CHMI was determined as successful infection of participants as determined by development of detectable parasitaemia.
To assess the immune response to primary, secondary and tertiary homologous and heterologous Plasmodium infection, through experimental injection of P. vivax and P. falciparum-infected erythrocytes.
Antibody responses to blood-stage P. vivax antigens were measured. Data from the following participants were pooled for assessment: all primary P. vivax CHMI, all secondary P. vivax CHMI, all tertiary P. vivax CHMI and all P. falciparum heterologous CHMI.
The effect of any anti-parasite immunity on parasite growth dynamics was assessed by measurement of qPCR during CHMI. Descriptive graphs showing the qPCR over time for each participant and the medians for primary, secondary and tertiary CHMI were presented. Parasite multiplication rate was calculated for each individual using a linear model fitted to log10 transformed qPCR data. Comparisons between two groups were made using Wilcoxon signed rank test.
To assess gametocytaemia following primary, secondary and tertiary P. vivax infection through experimental injection of P. vivax infected erythrocytes.
To assess transmission of P. vivax gametocytes from infected volunteers to mosquito vectors
These last two secondary objectives are not reported here and will be reported in a separate publication.

VAC079 was a vaccine efficacy study and the primary and secondary objectives of this study were published previously (PMID 37437014). Only descriptive clinical data following CHMI in VAC079 are included this manuscript to support the findings from the VAC069 study.

Plants

Seed stocks	*Report on the source of all seed stocks or other plant material used. If applicable, state the seed stock centre and catalogue number. If plant specimens were collected from the field, describe the collection location, date and sampling procedures.*
Novel plant genotypes	*Describe the methods by which all novel plant genotypes were produced. This includes those generated by transgenic approaches, gene editing, chemical/radiation-based mutagenesis and hybridization. For transgenic lines, describe the transformation method, the number of independent lines analyzed and the generation upon which experiments were performed. For gene-edited lines, describe the editor used, the endogenous sequence targeted for editing, the targeting guide RNA sequence (if applicable) and how the editor was applied.*
Authentication	*Describe any authentication procedures for each seed stock used or novel genotype generated. Describe any experiments used to assess the effect of a mutation and, where applicable, how potential secondary effects (e.g. second site T-DNA insertions, mosiacism, off-target gene editing) were examined.*
